# Somatosensory cortex shapes perceptual decision bias via the superior colliculus

**DOI:** 10.1038/s41467-026-78331-0

**Published:** 2026-10-10

**Authors:** Alice Y. Nam, Jiwook Shin, Morgan Tenney, Baihe Zhang, Y. Kate Hong

**Affiliations:** 1 Department of Biological Sciences, Carnegie Mellon University, Pittsburgh, PA USA; 2 Neuroscience Institute, Carnegie Mellon University, Pittsburgh, PA USA; 3 Department of Neuroscience, University of Pittsburgh, Pittsburgh, PA USA

**Keywords:** Whisker system, Decision

## Abstract

Sensory inputs reach cortical and subcortical areas in parallel, but how they coordinate to guide decisions remains unknown. We examined the roles of the primary somatosensory cortex (S1) and the superior colliculus (SC) in mice performing whisker-mediated tactile detection. While inactivating S1 contralateral to the stimulus increased detection threshold, inactivating ipsilateral S1 decreased it. These opposing effects reflected shifts in choice bias rather than sensitivity. Unilateral SC inactivation produced similar directional effects, whereas bilateral inactivation of either S1 or SC neutralized bias. Furthermore, SC lesions impaired detection performance, and combined S1 inactivation caused no further impairment, indicating that S1-mediated shifts in bias were SC-dependent. S1 inactivation also decreased sensory-evoked neural activity in SC, and choice decoding revealed that behavioral impairment was more closely associated with changes in SC population activity than with loss of sensory encoding in S1. Thus, S1 shapes perceptual bias by modulating SC-dependent circuits that transform sensory evidence into perceptual decisions.

## Abstract (web summary)

The authors show that, when detecting the presence of tactile stimuli, mouse primary somatosensory cortex shapes perceptual decisions through the midbrain superior colliculus, primarily by shifting decision bias rather than the ability to detect the stimulus.

## Introduction

Perceptual decisions depend not only on the fidelity of sensory encoding, but also on how sensory evidence is weighed against internal decision criteria. Causal perturbations are commonly used to infer the functional necessity of a brain region. However, behavioral deficits that accompany such perturbations can be difficult to interpret. For example, inactivation of a cortical area may impair behavioral performance by reducing perceptual sensitivity, but could also alter choice bias, defined as the systematic tendency to favor one behavioral response over another independent of sensory evidence. Distinguishing between these possibilities is critical for understanding how sensory areas contribute to perception.

The primary somatosensory cortex (S1) is the main cortical target for tactile input. Across primates and rodents, S1 perturbations severely impair discrimination of fine tactile features such as texture, shape, and object location^1–8^, whereas the effects on simple tactile detection are less clear^6,8,9^. In the rodent whisker system, acute S1 inactivation only partially impairs tactile detection^9–12^, and chronic whisker S1 lesions lead to rapid recovery^3,6,8,9,13,14^, suggesting that alternative pathways can support detection behavior^15^.

The superior colliculus (SC) is well-positioned to mediate tactile detection behavior. Although mostly studied for its roles in vision, eye movements, and head-orienting, the SC is an evolutionarily conserved midbrain structure that mediates rapid detection of multimodal stimuli to coordinate behavioral responses. Beyond its role in sensorimotor transformation, recent work highlights SC’s expanded role in more cognitive processes, including attention and perceptual decision-making^16–23^. In addition, relative differences in activity between two SC hemispheres have been proposed to account for perceptual decisions during a visual attention task, suggesting that interhemispheric SC balance may contribute to choice bias^24^.

SC neurons in the intermediate and deep layers respond to somatosensory stimuli^25–27^ and provide a subcortical pathway that could mediate tactile detection in the absence of S1^15^. Primary sensory cortices, including visual and whisker S1, directly and indirectly project to SC, where they have been shown to modulate sensory gain or drive SC-mediated behavior^28–32^. However, how primary sensory cortices and SC coordinate activity to guide behavior remains poorly understood.

Here, we examined how S1-SC interactions contribute to shaping perceptual choices. In mice performing a whisker-mediated tactile detection task, contralateral S1 inactivation impaired behavioral performance, not simply by decreasing sensitivity, but by shifting choice bias. Neural recordings revealed that S1 inactivation reduced tactile stimulus encoding in SC and altered the balance of activity across SC hemispheres. Moreover, the stimulus-evoked SC activity remained strongly predictive of trial-by-trial choice, even when S1 was silenced, and disrupting SC abolished the behavioral effect of S1 inactivation. Together, these results are consistent with a model in which S1 contributes to tactile detection not only by encoding sensory information, but by modulating SC-dependent circuits that determine how tactile evidence is converted into perceptual decisions.

## Results

### Contralateral S1 inactivation shifts decision bias but not sensitivity

**Fig. 1:**
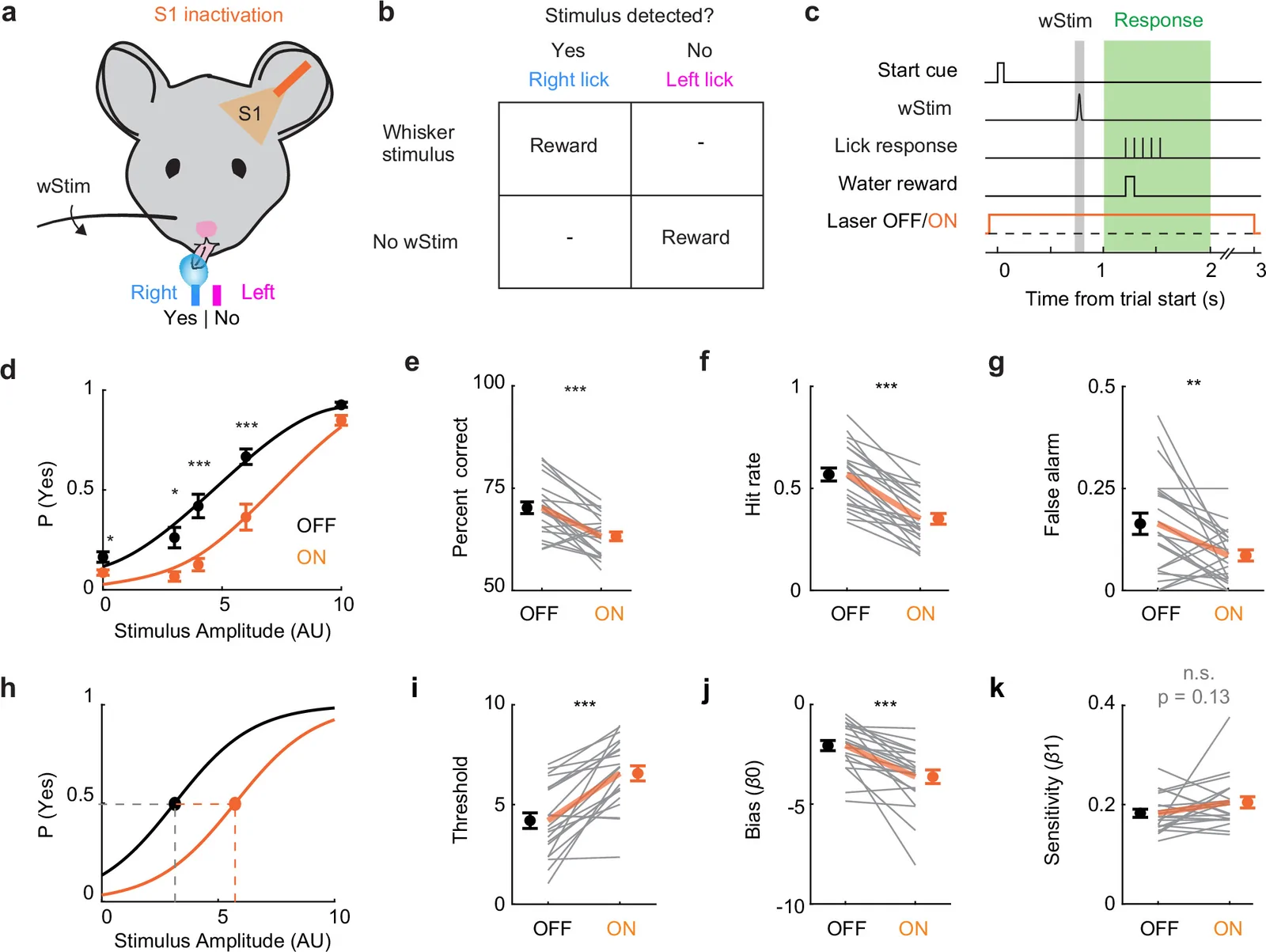
S1 inactivation impairs detection due to a shift in decision bias. **a** Head-fixed mice were trained to report the detection of a tactile stimulus delivered to a single (right, C2) whisker (wStim). Mice respond by licking right (ipsilateral to stimulus, cyan) to report stimulus detection (Yes) or left (contralateral to stimulus, magenta) to report the absence of a stimulus (No). **b** Subjects received a water reward for correct responses, and no reward for incorrect responses. **c** Trial structure: an auditory start cue was followed by a whisker stimulus of amplitude 0–10 arbitrary units (AU, 0–40^o^ downward deflection, ~ 50 ms pulse). After a 300 ms delay, the response window opened (2 s, green shaded area), followed by a 2-3 s inter-trial interval. Contralateral S1 was optogenetically inactivated (ON) on randomly interleaved trials for the full trial duration. **d** Probability of Yes-response (P(Yes)) across stimulus amplitudes (*n* = 22 sessions from 6 mice; two-sided Wilcoxon signed-rank test for OFF vs. ON at each amplitude with Bonferroni correction: *p* = 0.026, 0.018, 0.00044, 0.00088, 0.059, for stimulus amps 0, 3, 4, 6, and 10, respectively). **e** Percent correct (*p* = 2.94 × 10^−4^). **f** Hit rate (4.01 × 10^−5^). **g** False alarm rate (*p* = 5.14 × 10^−3^). **h** Example psychometric curves from one session with detection threshold indicated by dashed lines. **i** Detection threshold (*p* = 9.22 × 10^−5^). **j** Bias (*β*₀, *p* = 6.90 × 10^−5^). **k** Sensitivity (*β*1, *p* = 0.13). Laser-OFF (black); laser-ON (optogenetic inactivation, orange). **e**–**g**, **i**–**k** gray lines represent individual sessions; two-sided Wilcoxon signed-rank tests. **d**–**g** *n *= 22 sessions from 6 mice, (**i**–**k**): *n* = 21 sessions from 6 mice. * *p* < 0.05, ** *p* < 0.01, *** *p* < 0.001, n.s., not significant (*p* ≥ 0.05). Error bars are shown as mean ± s.e.m.; mixed-effects analyses accounting for repeated sessions within mouse yielded the same conclusions. Source data are provided as a Source Data file.

To determine the role of S1 in perceptual decision-making, we trained animals on a head-fixed, whisker-mediated tactile detection task. To minimize confounds from variability in whisking, we used passive stimulation of the ‘C2’ whisker on the animal’s right side with a single downward deflection, sampling a broad range of stimulus amplitudes spanning high- and low-salience stimuli to ensure comprehensive coverage of the perceptual range (0–10 arbitrary units (AU), calibrated to a maximum 40^o^ whisker angle, or ~ 2100^o^ /s) (Supplementary Fig. 1c, d). We implemented a forced-choice (Yes/No) paradigm, in which animals indicated their choice by licking one of two lick ports: right for stimulus detection (Yes) or left to indicate stimulus absence (No). Correct responses were rewarded with a drop of water (Fig. 1a, b). This forced-choice paradigm requires an overt response on every trial, allowing changes in detection behavior to be distinguished from failures to respond.

Tactile inputs from one side of the body cross the midline at the level of the brainstem to reach the contralateral thalamus and S1^33,34^. Here, we refer to contralateral and ipsilateral sides relative to the tactile stimulus, which was always delivered to the right side of the animal. We optogenetically silenced contralateral S1 (S1_contra_) in Emx1-Halo mice, which express inhibitory halorhodopsin (eNpHR3.0) in ~80% of excitatory cortical neurons^9,35^. Consistent with numerous studies^4–6,9–11,14^, acute S1_contra_ suppression (laser-ON) decreased overall performance (Fig. 1e) driven by an overall decrease in Yes responses across stimulus amplitudes (Fig. 1d), including no-stimulus trials (Fig. 1g, false alarms). Because the forced-choice task design requires animals to always report their decision, the global decrease in Yes responses was not simply due to disengagement or impaired ability to respond during laser-ON trials. In laser control experiments, in which the laser illumination was visible but did not alter S1 activity, no changes in whisker deflection amplitude or behavior were observed (Supplementary Fig. 1c–i).

To further dissect the observed changes in threshold, we generated psychometric curves by fitting logistic regression models to trial-by-trial responses as a function of stimulus amplitude for each session (Fig. 1h). As expected^9,11,13^, S1_contra_ inactivation significantly increased the detection threshold, equivalent to the stimulus amplitude needed to respond Yes 50% of the time (Fig. 1h, i). To further dissect how S1 inactivation altered psychometric performance, we considered two logistic regression parameters: decision bias (*β*_0_, intercept) and sensitivity (*β*_1_, slope), which together determine the detection threshold (illustrated in Supplementary Fig. 2, Eq. (1)). Decision bias reflects the subject’s overall tendency to favor one choice over another (e.g., Yes vs. No), independent of stimulus strength, and is reflected by a lateral shift in the psychometric curve; sensitivity captures how reliably choices scale with changes in stimulus amplitude, and is reflected by the slope of the psychometric curve.

S1_contra_ inactivation induced a significant decrease in decision bias (Fig. 1j; median ON-OFF difference = − 1.14, 95% bootstrap CI [− 1.69, − 0.787]; two-sided Wilcoxon signed-rank test, W = 1, *p* = 6.90 × 10−^5^, rank-biserial *r* = − 0.991). This is consistent with a conservative bias shift, in which subjects are less likely to respond Yes, regardless of the stimulus amplitude. In contrast, average sensitivity did not differ, with S1 inactivation leading to small or inconsistent changes across sessions (Fig. 1k; median ON-OFF = 0.036, 95% bootstrap CI [− 0.0170, 0.0829]; two-sided Wilcoxon signed-rank test, *W* = 159, *p* = 0.131, rank-biserial *r* = 0.377; *n* = 21 sessions from 6 mice). Together, these results indicate that the primary effect of S1_contra_ inactivation was to increase detection threshold due to a systematic shift in decision bias, rather than a reliable change in sensitivity.

### SC_contra_ inactivation shifts choice bias, similar to S1_contra_ inactivation

**Fig. 2:**
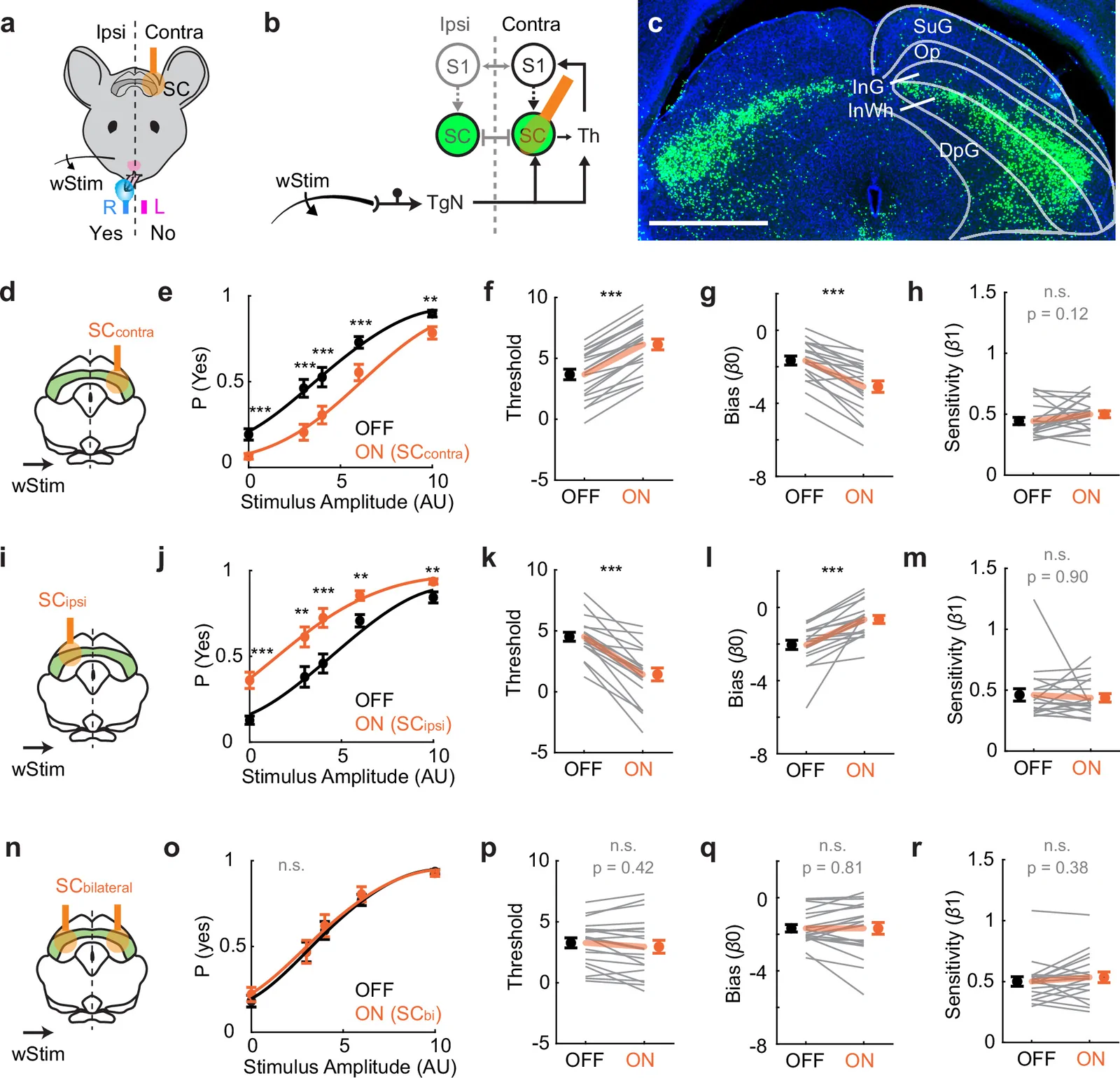
Inactivation of the superior colliculus (SC) directionally shifts action selection. **a** Schematic of Yes-No detection task in Pitx2-Halo mice with SC inactivation. Right (R, cyan) and Left (L, magenta) lick ports. **b** Simplified circuit diagram highlighting major somatosensory pathways to SC. Whisker stimuli (wStim) are transduced via the trigeminal ganglia and relayed through the trigeminal brainstem nuclei (TgN), which project to SC, and indirectly to S1 via the thalamus (Th). S1 projects both directly and indirectly to SC in the same hemisphere; SC, in turn, projects to the secondary somatosensory thalamus. **c** Pitx2-Halo mice were used to silence excitatory neurons in the intermediate SC, where whisker-responsive and premotor neurons are located (image from Pitx2-GFP showing targeted cells). **d**–**h** Effects of SC_contra_ inactivation (*n* = 21 sessions, 6 mice). **e** Logistic fits of average probability of Yes-response (P(Yes)); *p* = 8.14 × 10^−4^, 9.10 × 10^−4^, 2.94 × 10^−4^, 3.45 × 10^−4^, 1.61 × 10^−3^, for stimulus amps 0, 3, 4, 6, and 10, respectively. **f** Detection threshold, *p* = 5.96 × 10^−5^; **g** decision bias (*β*₀), *p* = 7.98 × 10^−5^; and **h** sensitivity (*β*1), *p* = 0.12. **i**–**m** SC_ipsi_ inactivation (*n* = 19 sessions, 6 mice). **j** P(Yes) by stimulus amplitude *p* = 6.59 × 10^−4^, 3.62 × 10^−3^, 9.11 × 10^−4^, 1.99 × 10^−3^, 4.23 × 10^−3^, for amps 0, 3, 4, 6, and 10, respectively. **k** Detection threshold, *p* = 1.32 × 10^−4^; (**l**) decision bias (*β*₀), *p* = 1.32 × 10^−4^; and (**m**) sensitivity (*β*1), *p* = 0.90. **n**–**r**: bilateral SC inactivation. **o** P(Yes) by amplitude; (**p**) Detection threshold; (**q**) decision bias (*β*₀), and (**r**), sensitivity (*β*1); (**o**), *n* = 20 sessions; (**p**–**r**), *n* = 19 sessions from 6 mice. For all plots, error bars represent mean ± s.e.m.; two-sided Wilcoxon signed-rank test. Bonferroni correction was applied in (**e**, **j**, and **o**); ** *p* < 0.01, *** *p* < 0.001, n.s., not significant. Mixed-effects analyses yielded the same conclusions (see Source Data file). Abbreviations: superficial gray (SuG), optic layer (Op). Schematics and contours in (**c**, **d**, **i**, **n**), adapted from Allen Reference Atlas – Mouse Brain, atlas.brain-map.org^72^. Source data are provided as a Source Data file.

The intermediate layers of the SC (Fig. 2b, c) receive direct ascending input from the contralateral trigeminal brainstem nuclei, providing a direct pathway for bottom-up tactile information, independent of S1^31,32^ (Fig. 2b). Previous work has shown that SC can partially mediate tactile behaviors in a conditioned foot-shock avoidance task^15,36^, consistent with SC’s general role in mediating stimulus-induced escape behaviors^16,36,37^. This raises the possibility that SC may also contribute directly to perceptual decisions during operant tasks.

To determine the role of SC during tactile detection, we optogenetically inactivated the intermediate layers of central SC, contralateral to the whisker stimulus (SC_contra_), where S1 neurons directly project, and C2 whisker-responsive neurons reside (Supplementary Fig. 3c and Supplementary Fig. 7b, d). We used Pitx2-Cre knock-in mice to drive stable expression of halorhodopsin (Pitx2-Halo), which selectively expresses inhibitory halorhodopsin in around half of excitatory neurons in the intermediate SC^38^ (Fig. 2c). Pitx2-Cre-expressing neurons include a subset of neurons that receive direct input from S1 as well as the trigeminal brainstem nuclei^39^, and have been shown to represent premotor neurons that mediate head-orienting movements^38^. We first validated functional silencing in Pitx2-Halo mice by recording from the C2 whisker-responsive region of SC. Optogenetic inactivation of Pitx2-Halo neurons partially reduced spontaneous firing and sensory-evoked activity, including both raw evoked firing rates and baseline-subtracted response amplitudes (Supplementary Fig. 4a–g). We next determined the effect of SC inactivation on behavioral performance. Despite limited overall suppression of SC activity, photoinhibition of SC_contra_ in Pitx2-Halo mice produced strong and consistent increases in detection threshold and decreased probability of Yes response (ipsilateral / right licks) across all amplitudes, leading to a decrease in bias (Fig. 2d–g), strikingly similar to the effects of S1_contra_ inactivation (Fig. 1j).

Recent studies suggest that SC can influence S1 indirectly via the higher-order somatosensory thalamus (posteromedial thalamus, POm)^40^, which in turn projects to S1 (Fig. 2b). This raised the possibility that Pitx2-Halo-mediated SC inactivation might indirectly suppress S1 activity, accounting for the similar effects of S1_contra_ and SC_contra_ inactivation on behavior. To test this, we recorded across cortical layers of the C2 barrel in S1_contra_ during SC_contra_ inactivation. We did not detect significant changes in sensory-evoked activity in S1_contra_ with SC inactivation (Supplementary Fig. 4k, l, n), suggesting that the behavioral effects of SC_contra_ inactivation in Pitx2-Halo mice were unlikely to be the result of indirect suppression of sensory responses in S1.

### Unilateral SC inactivation bidirectionally shifts lateralized choice bias

Recent studies across primates and rodents have shown that unilateral perturbation of SC can directionally bias perceptual decisions: increasing SC activity biases attention and choices towards the contralateral side, while decreasing SC activity biases decisions towards stimuli presented ipsilaterally^20,23,41,42^. These effects are thought to stem from competitive dynamics between the two SC hemispheres, which may exert mutual inhibition to mediate lateralized choice selection^21,24,43,44^.

To determine whether SC inactivation can bidirectionally modulate choice bias during our tactile detection task, we next inactivated SC ipsilateral to the whisker stimulus (SC_ipsi,_ Fig. 2i). Strikingly, SC_ipsi_ inactivation had the opposite effect from SC_contra_ inactivation, significantly decreasing the detection threshold (Fig. 2k) and increasing bias (Fig. 2l) due to an overall increase in Yes responses (contralateral licks) across all stimulus amplitudes (Fig. 2j). Thus, unilateral SC inactivation shifts choice bias in opposite directions depending on the targeted hemisphere.

The opposing effects of SC_contra_ and SC_ipsi_ inactivation suggested that the relative activity between the two SC hemispheres can bias choice. Consistent with this notion, bilateral SC inactivation had no significant effect on behavioral performance, including overall detection threshold and bias (Fig. 2p: two-sided Wilcoxon signed-rank test, *W* = 75, *p* = 0.421, *n* = 19 sessions; median ON-OFF difference: − 0.165, 95% bootstrap CI [− 0.760, 0.322], rank-biserial *r* = − 0.211; Fig. 2q, bias: two-sided Wilcoxon signed-rank test, *W* = 101, *p* = 0.81, *n* = 19 sessions in 6 mice; median ON-OFF difference: 0.0536, 95% bootstrap CI [− 0.418, 0.561], rank-biserial *r* = 0.0631). Sensitivity was not significantly altered by contra-, ipsi-, or bilateral SC inactivation (Fig. 2h, m, r; all *p* > 0.05, two-sided Wilcoxon signed-rank tests).

SC-driven bias can reflect distinct processes across tasks and subregions. In particular, inactivation of the lateral SC region has been shown to inhibit contralateral licking, while medial SC does not^21^. Given that the C2 whisker-responsive region targeted for inactivation lies proximal to the lateral SC region that has been implicated in premotor control of licking^21,43,45^, we also considered whether the behavioral bias effects we observed during unilateral SC inactivation were due to a general impairment in direction-specific licking. If so, we would expect unilateral SC inactivation to increase contraversive lick latencies broadly across stimulus amplitudes. Unilateral SC inactivation induced stimulus amplitude-dependent changes in lick latency that depended on the side of inactivation. Contralateral (left) SC inactivation increased Yes/right lick latencies only at higher stimulus amplitudes (amp 6 and 10), whereas ipsilateral (right) SC inactivation increased No/left lick latencies on no-stimulus trials (amp 0). Thus, SC inactivation did not uniformly slow licking across stimulus conditions (Supplementary Fig. 5b). Bilateral SC inactivation, despite its minimal impact on behavioral performance (Fig. 2, n-r), similarly increased lick latencies for Yes/right responses at higher stimulus amplitudes (Supplementary Fig. 5b, Bilateral, amps 6 and 10), with no detectable changes at weaker amplitudes or for No/left licks across any amplitude.

Thus, unilateral SC inactivation in Pitx2-Halo mice produced lateralized choice bias, whereas bilateral SC inactivation neutralized this effect. The changes in lick latencies suggest that the choice bias during direct SC-perturbation is at least in part due to a motor bias, consistent with the known role of SC in mediating lateralized licking movements^21^. However, the absence of changes in lick latency across amplitudes argues against global motor impairment. Taken together, these findings are consistent with models^24,44^ in which choice bias is shaped by relative activity across SC hemispheres rather than by absolute activity within either SC hemisphere alone.

### S1 activity shapes the direction of choice bias

**Fig. 3:**
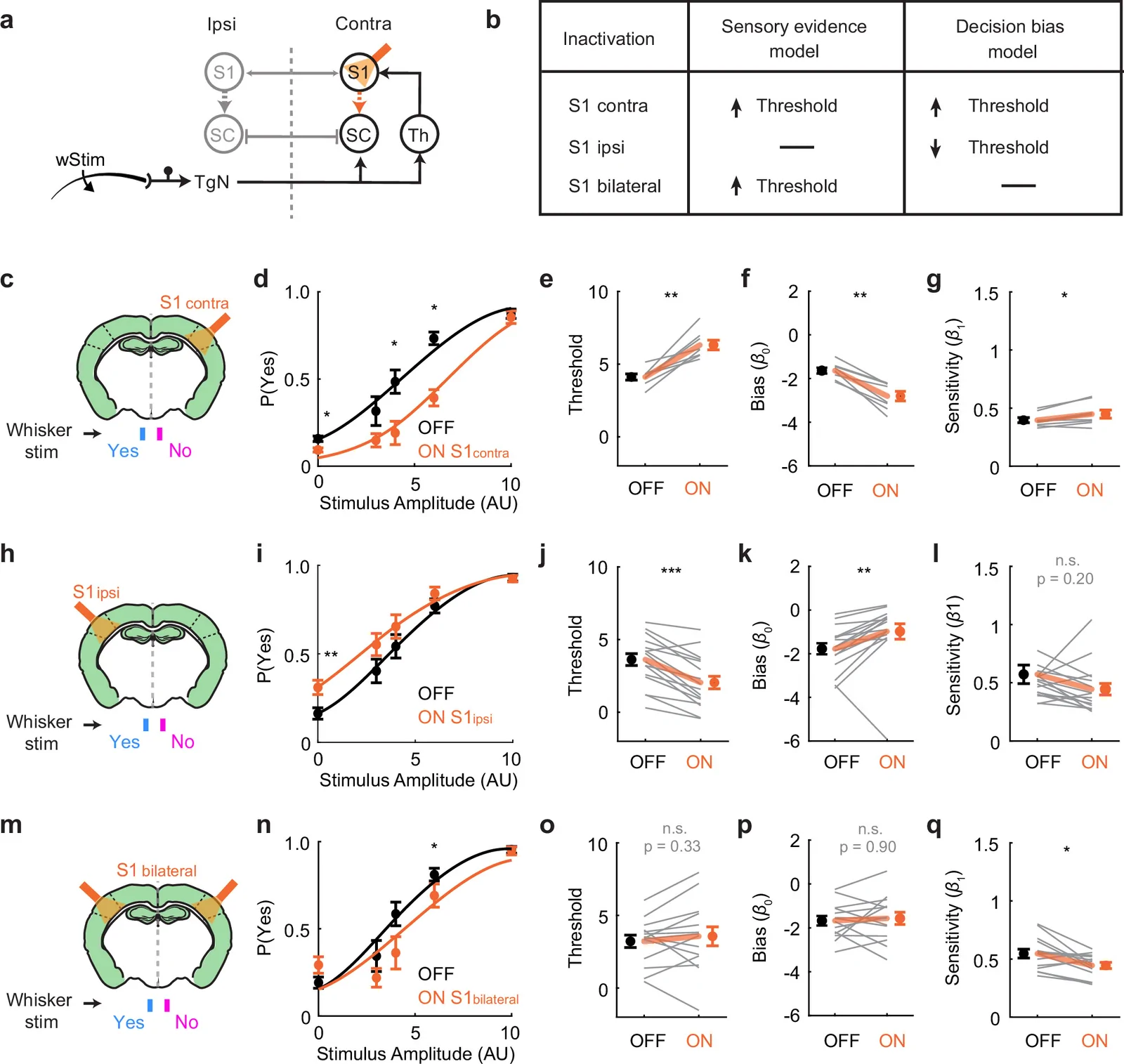
S1 inactivation induces directional shifts in decision bias. **a** Schematic of simplified S1 and SC pathways for tactile detection. A whisker stimulus (wStim) activates the trigeminal brainstem nuclei (TgN), which send ascending inputs to the contralateral S1 via the thalamus (Th). S1 projects directly and indirectly to SC in the same hemisphere (dashed orange arrow). **b** Conceptual models for S1’s primary role in detection behavior. Sensory evidence model**:** S1 encodes critical sensory information needed for detection. Decision bias model**:** S1 modulates downstream SC to bias decisions, rather than acting solely as a sensory relay. Predicted outcomes for the threshold with inactivation are shown. Arrows indicate predicted increase or decrease; dashes suggest minimal change. **c**–**g** S1_contra_ inactivation during detection behavior (*n *= 3 mice, 8 sessions). **d** Probability of Yes response (P(Yes)) by stimulus amplitude: *p* = 0.039, 0.39, 0.039, 0.039, 1.00, for amps 0, 3, 4, 6, and 10, respectively. **e** Detection threshold, *p* = 7.81 × 10^−3^; **f** decision bias (*β*₀), *p* = 7.81 × 10^−3^; and (**g**) sensitivity (*β*1), *p* = 0.023. **h**–**l**: ipsilateral S1 inactivation; **i** (P(Yes)) by stimulus amplitude (*p* = 0.0027, 0.37, 0.056, 0.096, and 1 for amps 0, 3, 4, 6, and 10, respectively; *n* = 17 sessions from 6 mice). **j** Detection threshold, *p* = 6.43 × 10^−4^; (**k**) decision bias (*β*₀), *p* = 7.17 × 10^−3^; and (**l**) sensitivity (*β*1), *p* = 0.20 (**j**–**l**, *n* = 16 sessions from 6 mice). **m**–**q** Bilateral S1 inactivation (*n* = 14 sessions from 6 mice). **n** (P(Yes)) by stimulus amplitude; *p = 0.034 at amp 6. **o** Detection threshold; (**p**) decision bias (*β*₀); and (**q**) sensitivity (*β*1), *p = 0.020. Shaded green areas (**c**, **h**, **m**) indicate the expression pattern of halorhodopsin in Emx1-Halo mice. All error bars represent mean ± s.e.m. All panels used two-sided Wilcoxon signed-rank tests (with Bonferroni correction for **d**, **i**, and **n**). Schematics in **c, h**, and **m** adapted from Allen Reference Atlas – Mouse Brain, atlas.brain-map.org^72^. Source data are provided as a Source Data file.

A common assumption is that inactivating S1 impairs detection behavior primarily by reducing the amount of sensory information in S1. However, S1 projects directly^31^ (Fig. 3a and Supplementary Fig. 3) and indirectly (via other cortical and basal ganglia pathways) to SC, and SC itself is known to robustly respond to tactile stimuli^15,21,46,47^. The striking similarity between behavioral effects of S1_contra_ and SC_contra_ inactivation led us to consider whether S1_contra_ inactivation impairs detection behavior primarily by modulating downstream SC activity to shift choice bias, rather than simply by disrupting sensory encoding within S1.

To test this hypothesis, we considered two competing models of S1’s role in tactile detection: a sensory evidence model, in which S1’s primary role is to encode tactile stimuli during the detection task (Fig. 3b, Sensory evidence model), or a choice bias model, in which S1 primarily shapes choice bias during the task (Fig. 3b, Decision bias model). Ascending whisker inputs to S1 are lateralized, primarily originating from the contralateral whiskers^32,48–51^. Although ipsilateral whisker responses are present in S1_contra_, these have been shown to originate from cross-callosal inputs from the opposite S1 hemisphere rather than uncrossed ascending input^50,51^. Thus, in the sensory evidence model, inactivation of S1 ipsilateral to the whisker stimulus (S1i_psi_) should have minimal behavioral impact. Furthermore, bilateral inactivation should impair detection at least as strongly as S1_contra_ inactivation, due to the loss of sensory responses in both hemispheres. In contrast, under the decision bias model, S1 influences detection behavior primarily through lateralized downstream effects on decision circuits such as SC. This model predicts that the effects of S1_ipsi_ and bilateral S1 inactivation should mirror those seen with equivalent SC manipulations: S1_ipsi_ should decrease detection threshold due to choice bias, and bilateral inactivation should neutralize bias, producing minimal effects on threshold (Fig. 3b, Decision bias model).

To directly test these model predictions, we trained a new cohort of Emx1-Halo mice and inactivated either contra-, ipsi-, or bilateral S1 in each session. For S1_contra_ inactivation (*n* = 8 sessions from 3 mice), detection threshold increased (Fig. 3e, median ON-OFF difference = 2.209, *n* = 8 sessions from 3 mice, 95% bootstrap CI [0.974, 3.067], *p* = 0.00781) and produced a conservative bias (Fig. 3f; median ON-OFF difference = − 1.105, 95% CI [− 1.696, − 0.701], *p* = 0.0078), consistent with our original experiments in Fig. 1. We observed a modest increase in sensitivity at the session level (Fig. 3g; median ON-OFF difference = 0.059, 95% CI [0.0059, 0.0873], *p* = 0.023), but this did not replicate in the larger cohort (Fig. 1k; median ON-OFF difference = 0.036, 95% CI [− 0.017, 0.083], *p* = 0.13, *n* = 21 sessions from 6 mice). In contrast, threshold and bias shifts were reproducible and consistent across cohorts (Figs. 1i, j, 3e, f). All comparisons used two-sided Wilcoxon signed-rank tests.

We next tested the effects of S1_ipsi_ inactivation, which surprisingly led to a consistent decrease in the detection threshold and a liberal bias shift, with animals more likely to say Yes across stimuli—even during no-stimulus trials (Fig. 3h–k). The decreased detection threshold was driven by an increase in bias (Fig. 3k), rather than due to enhanced sensitivity (Fig. 3l).

Bilateral S1 inactivation (*n* = 14 sessions from 6 mice) did not significantly alter detection threshold (Fig. 3o; median ON-OFF difference = 0.621, 95% CI [− 0.50, 1.56], *p* = 0.326, *n* = 14 sessions, two-sided Wilcoxon signed-rank test) or decision bias (Fig. 3p; median ON-OFF difference − 0.0478, 95% CI [− 0.56, 0.48], *p* = 0.903), but induced a modest decrease in detection probability for near-threshold amplitude 6 (Fig. 3n; median ON-OFF difference = − 0.121, 95% CI [− 0.20, − 0.020], *p* = 0.0336, two-sided Wilcoxon signed-rank test with Bonferroni correction). This revealed a small but significant decrease in sensitivity (Fig. 3q; median ON-OFF difference − 0.0766, 95% CI [− 0.19, − 0.010], *p* = 0.0202), suggesting that S1 contributes modestly to enhancing sensory processing near threshold, but this role may have been masked by the stronger effects of bias shifts induced by unilateral S1 inactivation.

Together, these results indicate that behavioral impairments during unilateral S1 inactivation are consistent with modulation of downstream circuits that shift choice bias, rather than loss of sensory information in S1—more closely aligned with the decision bias model than the sensory evidence model (Fig. 3b). Overall, these findings support a model in which S1 primarily shapes decision bias during tactile detection behavior, while making sensory contributions for near-threshold stimuli.

S1 projects to multiple downstream motor-related areas^11,52^ (e.g., primary and secondary motor cortices, SC, and basal ganglia), raising the possibility that the effects of unilateral S1 inactivation reflect a lateralized motor bias, rather than perceptual decision bias. To test this, we trained a new cohort of mice on a reversed-contingency task in which the motor mapping of Yes/No choices was swapped, allowing us to dissociate sensory-driven perceptual bias from lateralized motor bias. In the original paradigm, Yes was mapped to licking ipsilaterally toward the whisker stimulus, and S1_contra_ inactivation led to decreased Yes and ipsilateral lick responses. In the reversed motor contingency paradigm, animals were required to lick contralaterally, away from the same stimulus to respond Yes (Supplementary Fig. 6a, b). S1 inactivation under the reversed contingency still decreased Yes responses despite the opposite lick-direction mapping, consistent with a perceptual bias rather than directional motor bias (Supplementary Fig. 6c–f). Similarly, S1_ipsi_ inactivation during the reversed motor contingency paradigm increased Yes responses and contra-licking, consistent with a perceptual decision (Yes/No) rather than motor (left/right) bias. Moreover, we did not detect significant changes in lick latencies with unilateral or bilateral S1 inactivation, further suggesting that S1 inactivation-induced choice bias was not simply due to a motor bias (Supplementary Fig. 5c).

To further rule out lateralized motor impairments, we tested a new group of animals on a Go/No-go version of the task, in which detection responses were made by licking a single, central lick port, eliminating lateralized motor requirements. Even in this task, S1_contra_ inactivation increased detection threshold driven by a conservative bias shift, mirroring the Yes/No tasks (Supplementary Fig. 6k–n). Together, these results suggest that S1 modulates perceptual decision bias, rather than simply biasing lick direction.

### Stimulus encoding is greater in S1 than SC, but choice prediction is similar across areas

**Fig. 4:**
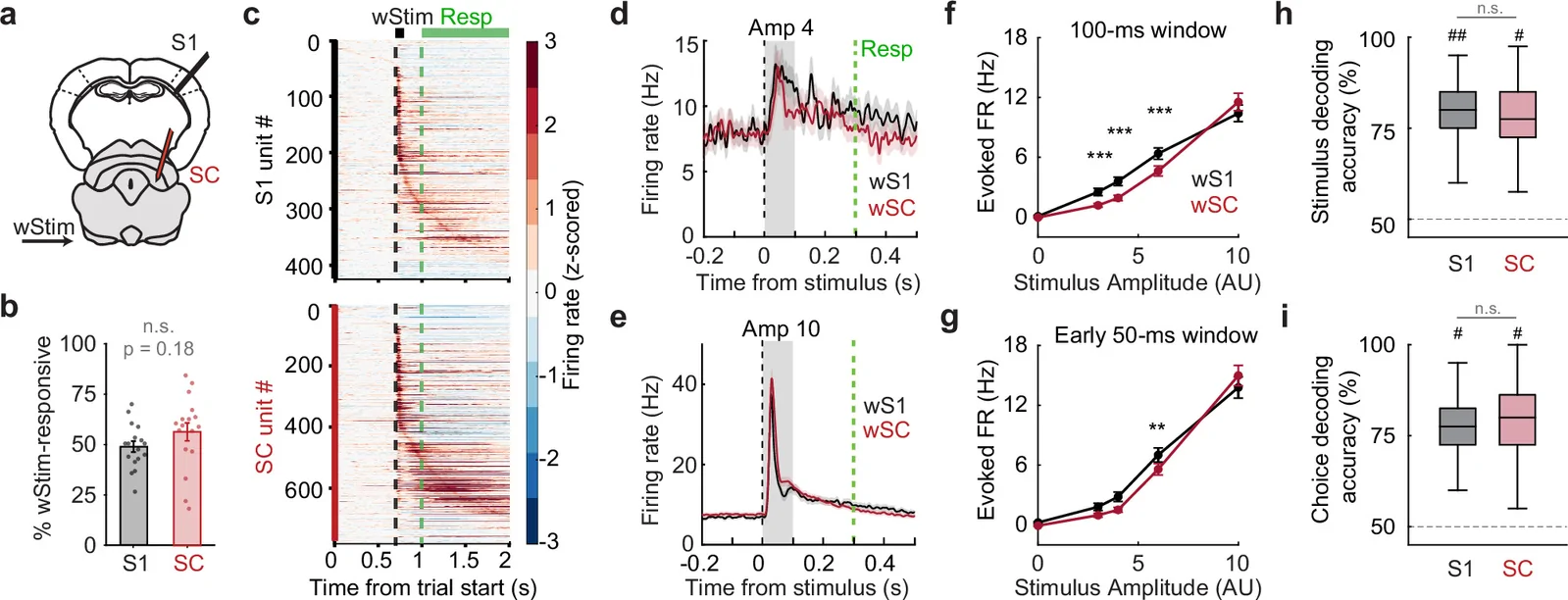
Sensory-evoked responses in S1 vs. SC during tactile detection behavior. **a** Simultaneous array recordings in S1 and SC contralateral to the whisker stimulus (wStim) during detection task performance. **b** Similar proportions of neurons responded during the stimulus window (100 ms from stimulus onset); *p* = 0.18, two-sided paired *t* test; *n* = 17 independent acute recording sessions from paired S1-SC recordings from 6 mice. **c** Z-scored firing rates for each single unit to stimulus (amp 10), sorted by peak firing time. Top: S1 (*n* = 423 units); bottom: SC (*n* = 778 units). **d** Average PSTHs of whisker-responsive neurons in S1 (wS1, black, *n* = 209 units) and SC (red, *n* = 411 units) for stimulus amp 4 and (**e**) amp 10. **f** Average baseline-subtracted firing rates (FR) of whisker stimulus-responsive S1 (wS1, *n* = 209 units) and SC (wSC, *n* = 411 units) neurons during the stimulus window (unit-level analysis; 17 sessions in 6 mice. Two-sided Wilcoxon rank-sum tests with Bonferroni correction across stimuli: amp 0, *p* = 1.00; amp 3, *p* = 5.43 × 10^−5^, amp 4, *p* = 2.44 × 10^−5^; amp 6, *p* = 3.00 x 10^−6^; amp 10, *p* = 1.00. ** *p* < 0.01, *** *p* < 0.001. **g** Same as in (**f**) but for a more restricted (50 ms) early stimulus window: amp 0, *p* = 0.241; amp 3, *p* = 0.434; amp 4, *p* = 0.155; amp 6, *p* = 0.00476; amp 10, *p* = 1.00. **h** Stimulus decoding (amp 0 vs. 6, S1: ## *p* = 0.0080, SC: # *p* = 0.014, shuffle-label null test); (**i**) choice decoding accuracy during the 100 ms stimulus window for near-threshold stimulus amp 6 (S1: # *p* = 0.013, SC: # *p* = 0.018). Data represented as mean ± s.e.m. in (**b**, **d**–**g**, **h**, **i**) show median decoding accuracies across 200 pseudopopulation resampling runs reflecting Monte Carlo variability, and whiskers extend to the most extreme values within 1.5 x the interquartile range. Horizontal dashed lines indicate chance level accuracy; # *p* < 0.05, ## *p* < 0.01: significant difference from 50% chance level, one-sided shuffle-label permutation test. Decoding used 282 units per pseudopopulation draw, matched across areas and choice direction. S1 vs. SC differences were tested using session-block permutation (See methods). Abbreviations: wStim: whisker stimulus, Resp, response window; FR, firing rate; wS1, wSC: whisker-stimulus responsive S1, SC neurons. Schematic in (**a**) adapted from Allen Reference Atlas – Mouse Brain, atlas.brain-map.org^72^. Source data are provided as a Source Data file.

SC receives direct bottom-up tactile inputs from the trigeminal brainstem nuclei, as well as top-down inputs from S1 (Supplementary Fig. 3). The behavioral similarity between S1 and SC inactivation suggested that S1 may influence detection behavior by modulating downstream SC activity. While the neural correlates of tactile detection have been extensively characterized in S1, how SC neurons contribute to this process and, further, how S1 and SC may together coordinate detection behavior, remain poorly understood. As a first step, we characterized how whisker stimuli are represented in S1 and SC during detection behavior. We simultaneously recorded from the C2 barrel in S1_contra_ and the somatotopically aligned, C2-responsive area in SC_contra_ during the Yes/No detection task (Fig. 4a and Supplementary Fig. 7; S1: *n* = 423 neurons; SC: *n* = 778 neurons; from *n* = 17 sessions across 6 mice).

Despite the heterogeneity of neurons in the intermediate SC layers, which include multisensory and premotor neurons, around half of SC neurons responded to C2 whisker deflection during behavior. This was not detectably different from simultaneously recorded neurons in the C2 barrel of S1 (Fig. 4b; S1, 48.5 ± 2.7%; SC, 55.8 ± 4.5% mean ± s.e.m.; mean paired difference, SC-S1 = 7.31 percentage points, 95% CI [− 3.78, 18.40]; two-sided paired *t* test, t(16) = 1.40, *p* = 0.18, Cohen’s d_z_ = 0.34). Activity in both areas tiled the period from stimulus onset (Fig. 4c, black dashed lines) through the response window (green lines, starting 300 ms after stimulus onset). S1 neurons had significantly higher stimulus-evoked responses for weaker stimuli compared with SC (Fig. 4f, *p* = S1, *n* = 209; SC, *n* = 411 whisker stimulus-responsive units). SC neurons have been shown to increase activity during motor action, such as whisking^53^, licking^21,45^, or preparatory motor activity^21,47,54^, which can confound stimulus activity from detection-induced pre-motor responses. We therefore tested a more restricted stimulus window of 50 ms from stimulus onset, which showed similar differences between S1 and SC (Fig. 4g). Thus, average stimulus-evoked firing rates indicate stronger encoding of low-amplitude tactile stimuli in S1 than in SC during detection behavior.

We determined how accurately S1 vs. SC population activity predicted stimulus presence and choice under normal (laser-OFF) conditions. To decode stimulus presence, we trained linear support vector machine (SVM) classifiers to distinguish between no-stimulus (amp 0) and near-threshold stimulus (amp 6) trials during the stimulus window (100 ms from stimulus onset), while balancing trials by lick direction. Both S1 and SC activity robustly predicted stimulus presence above chance (Fig. 4h, S1, *p* = 0.00799; SC, *p* = 0.014). No significant difference was detected between areas (S1 = 78.3%, SC = 80.3%; mean difference = − 2.0 percentage points, *p* = 0.70; two-sided session-block permutation test; *n* = 282 units per pseudopopulation draw from 15 paired sessions in 6 mice).

We next asked how accurately the population activity during the same stimulus window could predict the animal’s subsequent choice on a trial-by-trial basis. Choice was decoded significantly above chance from both S1 and SC (Fig. 4i; S1 mean accuracy = 77.5%, *p* = 0.013; SC mean accuracy = 79.5%, *p* = 0.018; one-sided label-shuffle permutation tests, 200 resampling runs). Choice decoding accuracy did not differ significantly between regions (S1 = 75.6%, SC = 78.1%; mean difference = − 2.5 percentage points, *p* = 0.755, two-sided session-block permutation test; 282 units per pseudopopulation draw from 11 paired sessions). Thus, both S1 and SC population activity carried decodable information about stimulus presence and subsequent choice.

### S1 inactivation decreases spontaneous and stimulus-evoked responses in SC

**Fig. 5:**
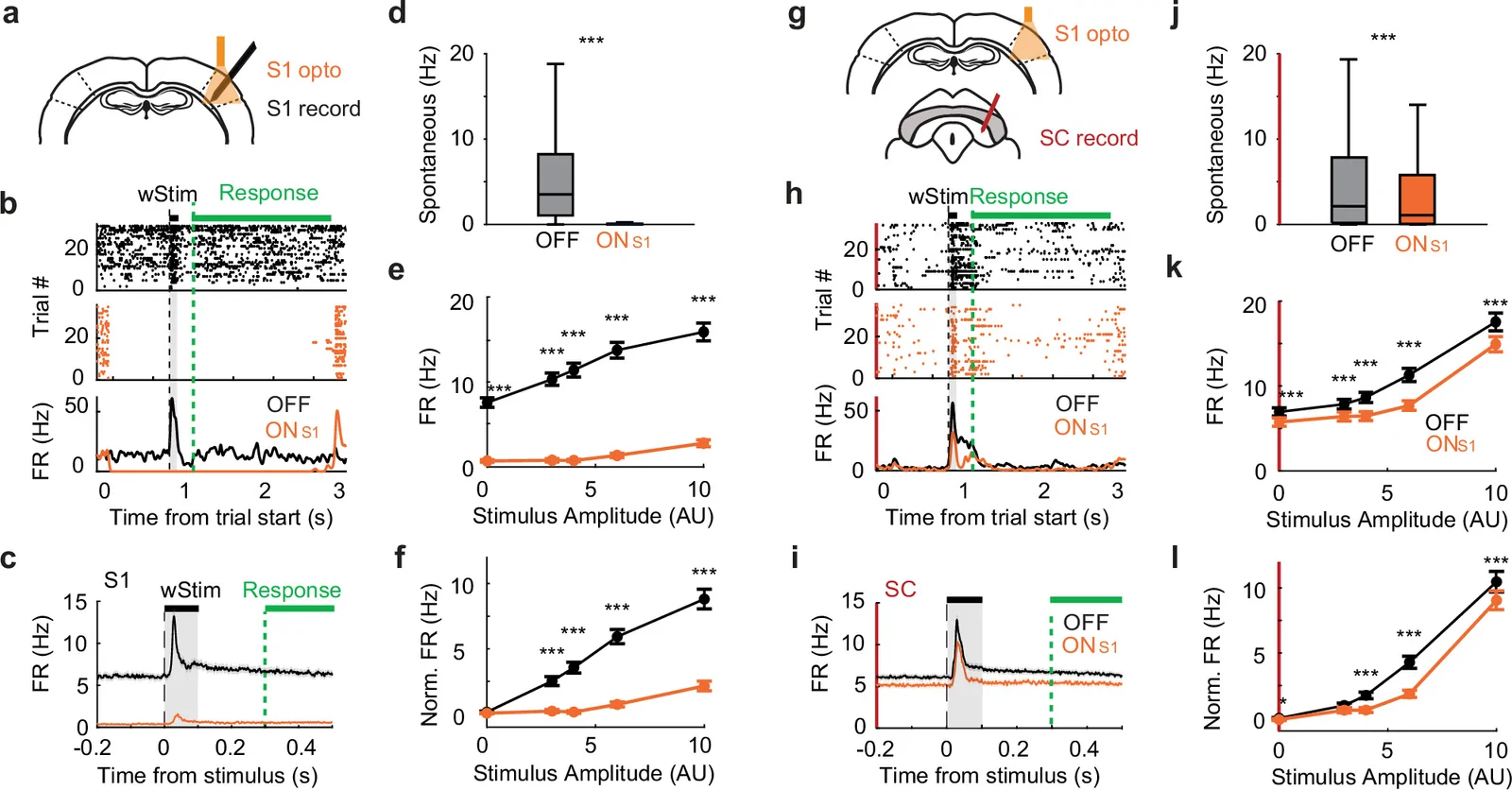
Inactivation of S1 decreases spontaneous and evoked firing rates in SC. **a**–**f:** S1 and (**g**–**l**)**:** SC recordings during S1_contra_ inactivation (6 mice, 17 sessions). **a** Schematic showing S1 recordings during S1 inactivation. **b** Raster plots and PSTHs for an example neuron in S1. **c** Average PSTH of S1 neurons across laser-OFF vs. ON_S1_ trials (*n* = 423 neurons); (**d**) S1_contra_ spontaneous firing rates (*p* = 3.39 × 10^−68^, *n* = 423 S1 units, 100 ms pre-stimulus window). **e** Average sensory-evoked firing rates of whisker-responsive units (*n* = 209 S1 units; *p* = 1.92 × 10^−32^, 1.24 × 10^−30^, 2.77 × 10^−32^, 1.63 × 10^−32^, and 2.43 × 10^−32^ for amps 0, 3, 4, 6, and 10, respectively). **f** Baseline-subtracted firing rates of same units in (**e**) (*p* = 1, 1.71 × 10^−^^14^, 1.56 × 10^−^^17^, 3.2 x 10^−^^23^, and 1.25 × 10^−^^16^ for amps 0, 3, 4, 6, and 10, respectively). **g** Schematic of SC_contra_ recordings during S1 inactivation. **h** Raster plots and PSTHs for an example neuron in SC. **i** Average PSTH of SC neurons across laser-OFF vs. ON_S1_ trials (*n* = 778 SC neurons); (**j**) SC_contra_ spontaneous firing rates (*p* = 3.69 × 10^−24^, *n* = 778 SC units). **k** Average sensory-evoked firing rates of whisker-responsive units (*n* = 411 SC units; *p* = 4.70 × 10^−^^16^, 8.35 × 10^−8^, 8.22 × 10^−12^, 5.39 × 10^−26^, and 9.13 × 10^−10^ for amps 0, 3, 4, 6, and 10, respectively). **l** Baseline-subtracted firing rates of the same units in (**k**) (*p* = 0.021, 0.419, 4.08 × 10^−^^4^, 5.06 × 10^−20^, and 8.66 × 10^−6^ for amps 0, 3, 4, 6, and 10, respectively). In (**b**, **c**, **h**, **i**)**:** black vertical dashed lines indicate stimulus onset; green dashed lines indicate start of response window; gray shaded boxes indicate 100 ms stimulus window. Dark lines and shaded error bars indicate mean ± s.e.m. For (**d**, **j**): median and interquartile range are shown, whiskers extend to the most extreme values within 1.5 x the interquartile range. For (**e**, **f**, **k**, **l**): data are presented as mean ± s.e.m, two-sided Wilcoxon signed-rank test, with Bonferroni correction for multiple comparisons. * *p* < 0.05, *** *p* < 0.001. Schematic in (**a**, **g**) adapted from Allen Reference Atlas – Mouse Brain, atlas.brain-map.org^72^. Source data are provided as a Source Data file.

We next asked whether S1_contra_ inactivation impairs behavior due to loss of sensory information in S1, or by disrupting activity downstream in SC_contra_. To test this, we analyzed the laser-ON trials during simultaneous recordings from S1_contra_ and SC_contra_. As previously reported^9^, optogenetic inactivation of S1_contra_ in Emx1-Halo mice was highly efficacious (Fig. 5a–f), locally suppressing 92.7% of overall baseline firing rates (Fig. 5d, OFF: 5.68 ± 0.36 Hz, ON: 0.42 ± 0.084 Hz, mean and s.e.m.), with the majority of neurons being significantly modulated (Supplementary Fig. 8a–c). Despite strong inactivation efficacy, some residual activity for high-amplitude stimuli remained (Fig. 5e, f; 15% residual activity for the strongest stimulus, amp 10).

In SC_contra_, S1_contra_ inactivation produced heterogeneous effects across single units, with some significantly decreasing activity (19% of single units), and a small proportion showing moderate increases (5% of single units, Supplementary Fig. 8d–f). Overall, there was a significant decrease in average spontaneous and stimulus-evoked activity across all stimulus amplitudes tested (Fig. 5j–l). Thus, S1_contra_ inactivation significantly decreased the baseline firing rates as well as sensory-evoked responses in SC_contra_, potentially impairing SC’s ability to mediate tactile detection behavior beyond its local effects in S1_contra_.

### Changes in SC activity, rather than S1, predict choice during S1 inactivation

**Fig. 6:**
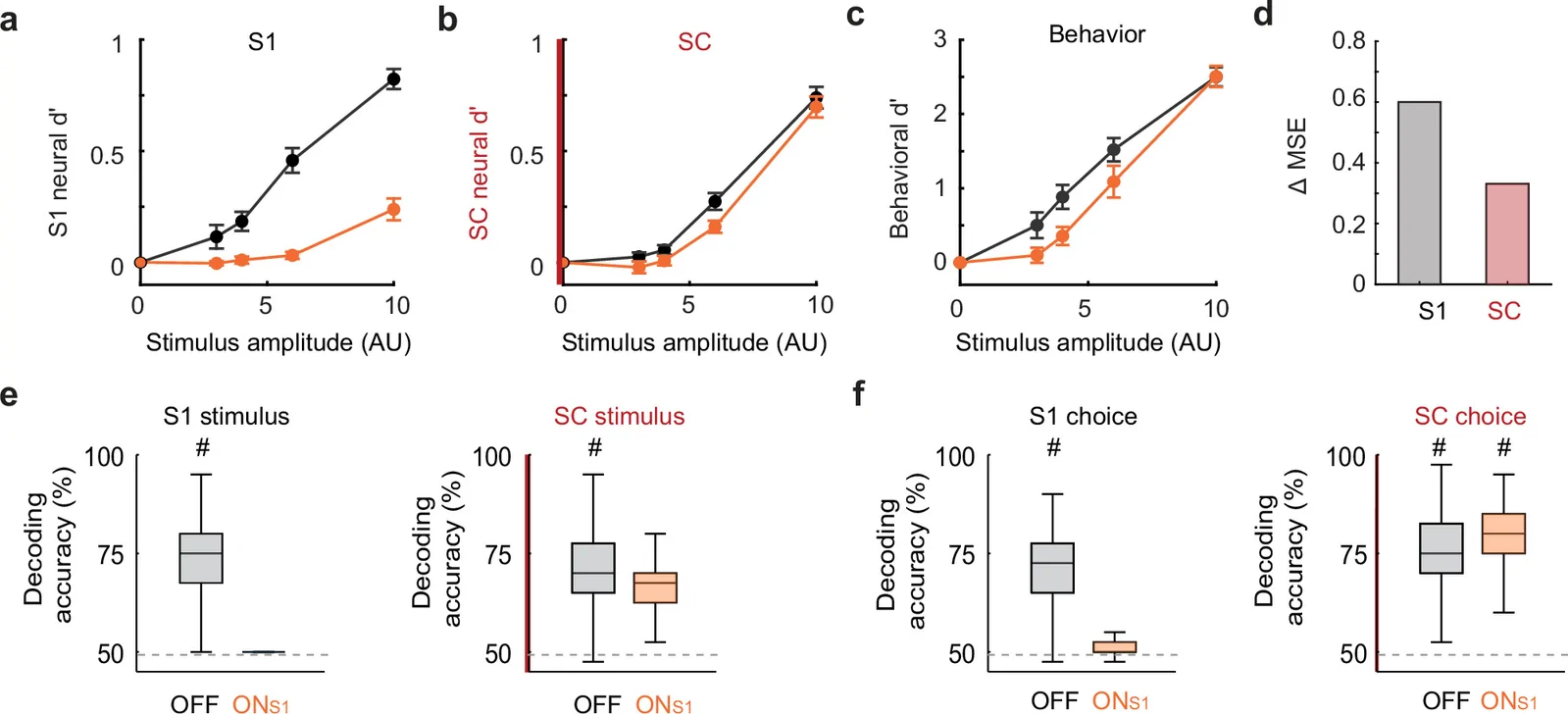
Behavioral changes with S1 inactivation are better captured by SC than S1 activity. **a** Neural discriminability (d’) for no-stimulus (amp 0) vs. each stimulus amplitude, shown for stimulus-responsive neurons from simultaneous recordings in S1 and (**b**) SC. Neural d’ was computed from AUROC values comparing stimulus vs. no-stimulus trials (S1, *n* = 202 stimulus-responsive neurons; SC, n = 433 stimulus-responsive neurons across 17 recording sessions from 6 mice). **c** Average behavioral d’ for each recording session under control (laser-OFF, black) vs. contralateral S1 inactivation (ON, orange). **d** Mean squared error (ΔMSE) between min-max normalized Δd’ curves (ON-OFF) from neural activity in S1, SC and behavior across non-zero amplitudes. A lower ΔMSE indicates a closer correspondence between the pattern of inactivation-induced change. **e**, **f** Stimulus and choice decoding accuracy in S1 and SC during laser-OFF and ON (S1_contra_ inactivation) conditions. **e** Stimulus decoding accuracy from S1 and SC population activity during the stimulus window (amp 0 vs. near-threshold amp 6, 100 ms from stimulus onset); difference from chance level for S1 OFF, *p* = 0.018; S1 ON, *p* = 0.63; for SC-OFF *p* = 0.041, SC ON, *p* = 0.051. **f** Choice decoding accuracy (Yes/No at near-threshold amp 6) for S1 and SC; difference from chance level for S1 OFF, *p* = 0.040; S1 ON, *p* = 0.35; SC, OFF: *p* = 0.019, ON: *p* = 0.015. Linear SVM classifiers were trained on laser-OFF trials and tested on held-out OFF or ON trials. Decoding used 119 units per pseudopopulation draw and 200 resampling runs for each brain area. Iterations represented repeated technical resampling of data pooled across 17 sessions in 6 mice. Error bars in (**a**–**c**)**:** mean ± s.e.m.; box plots in (**e**, **f**) indicate median and interquartile range, and whiskers extend to the most extreme values within 1.5 x the interquartile range. Dashed lines indicate chance level (50%) accuracy; # indicates accuracy significantly above the shuffled-label distribution, assessed using one-sided permutation tests. Source data are provided as a Source Data file.

Although photoinhibition of S1_contra_ substantially reduced S1_contra_ activity (Fig. 5b–f and Supplementary Fig. 8a–c), prior work suggests only a small amount of S1 activity may be needed to support stimulus detection^55,56^. We next asked whether decreased sensory-evoked responses in S1 or SC were more closely associated with behavioral impairments observed during S1_contra_ inactivation. To compare neural and behavioral effects, we quantified d’ (d-prime) across stimulus amplitudes for S1 and SC neural activity and behavioral choices. Consistent with efficient optogenetic silencing, S1_contra_ neural d’ during S1_contra_ inactivation (laser-ON trials) hovered around 0, with some residual discriminability at stronger amplitudes (Fig. 6a). In SC_contra_, S1_contra_ inactivation decreased neural d’ only for near-threshold stimulus amplitudes (Fig. 6b). Similarly, S1 inactivation decreased behavioral d’ only at near-threshold amplitudes (Fig. 6c). To compare how well S1 vs. SC captured the change in behavioral d’, we computed the difference in mean-squared error (ΔMSE) between neural and behavioral d’ (ON-OFF) profiles across stimulus amplitudes (“Methods”). A lower ΔMSE indicates a closer match in the pattern of inactivation-induced changes between neural and behavioral d’ curves, independent of absolute scale and offset. The ΔMSE was substantially lower for SC than S1 (Fig. 6d, S1: 0.60 vs. SC: 0.33), indicating that behavioral changes with S1 inactivation more closely mirrored changes in SC activity than S1.

To extend these analyses, we tested whether sensory-evoked activity in S1 or SC could reliably predict stimulus presence and behavioral choice on a trial-by-trial basis. We applied cross-condition decoding analyses using SVM classifiers trained on OFF trials and tested their performance on held-out OFF or ON trials. We restricted our analysis to the stimulus window (100 ms from stimulus onset for near-threshold amp 6) before licking or reward delivery. To further control for potential motor confounds, we matched trials by lick direction. When OFF-trained classifiers were applied to ON trials, stimulus decoding dropped to chance level in S1 (Fig. 6e; S1 OFF: mean decoding accuracy: 74.1%, 95% resampling interval [55.0, 92.5%], *p* = 0.018; ON_S1_: 50.0%, 95% resampling interval [45.0, 55.0%], *p* = 0.632; one-sided permutation tests using shuffled class labels). In SC, stimulus decoding during ON trials reached 66.31% but did not reach statistical significance (SC OFF: 70.6%, 95% resampling interval [55.0, 85.0%], *p* = 0.0410, ON_S1_: 66.3%, 95% resampling interval [51.3, 78.8%], *p* = 0.0509; one-sided permutation tests using shuffled class labels).

If residual S1 activity is responsible for the remaining ability to perform during S1 inactivation, then an OFF-trained S1 decoder should continue to predict the subject’s altered choice patterns on laser-ON trials. Instead, S1 choice decoding accuracy dropped to chance for laser-ON trials (Fig. 6f, S1, OFF: mean decoding accuracy: 71.5%, *p* = 0.040; ON _S1_: 50.8%, *p* = 0.35, one-sided permutation tests using shuffled class labels). In contrast, SC choice decoding accuracy remained robust across laser conditions (Fig. 6f, SC, OFF: 75.4%, *p* = 0.0190; ON_S1_: 79.1%, *p* = 0.0150, one-sided permutation tests using shuffled class labels), suggesting that SC population activity during the stimulus window continued to predict the subject’s choices on a trial-by-trial basis.

Together, these results suggest that behavioral impairments during S1 inactivation were more closely associated with changes in SC population activity during the stimulus window than with loss of S1 activity. S1 inactivation disrupted stimulus representations in both S1 and SC, but only SC activity reliably tracked behavioral choice across conditions, supporting a model in which SC, rather than residual activity in S1, better explains behavioral impairments observed during S1_contra_ inactivation.

### S1 inactivation-induced changes in behavior are SC-dependent

**Fig. 7:**
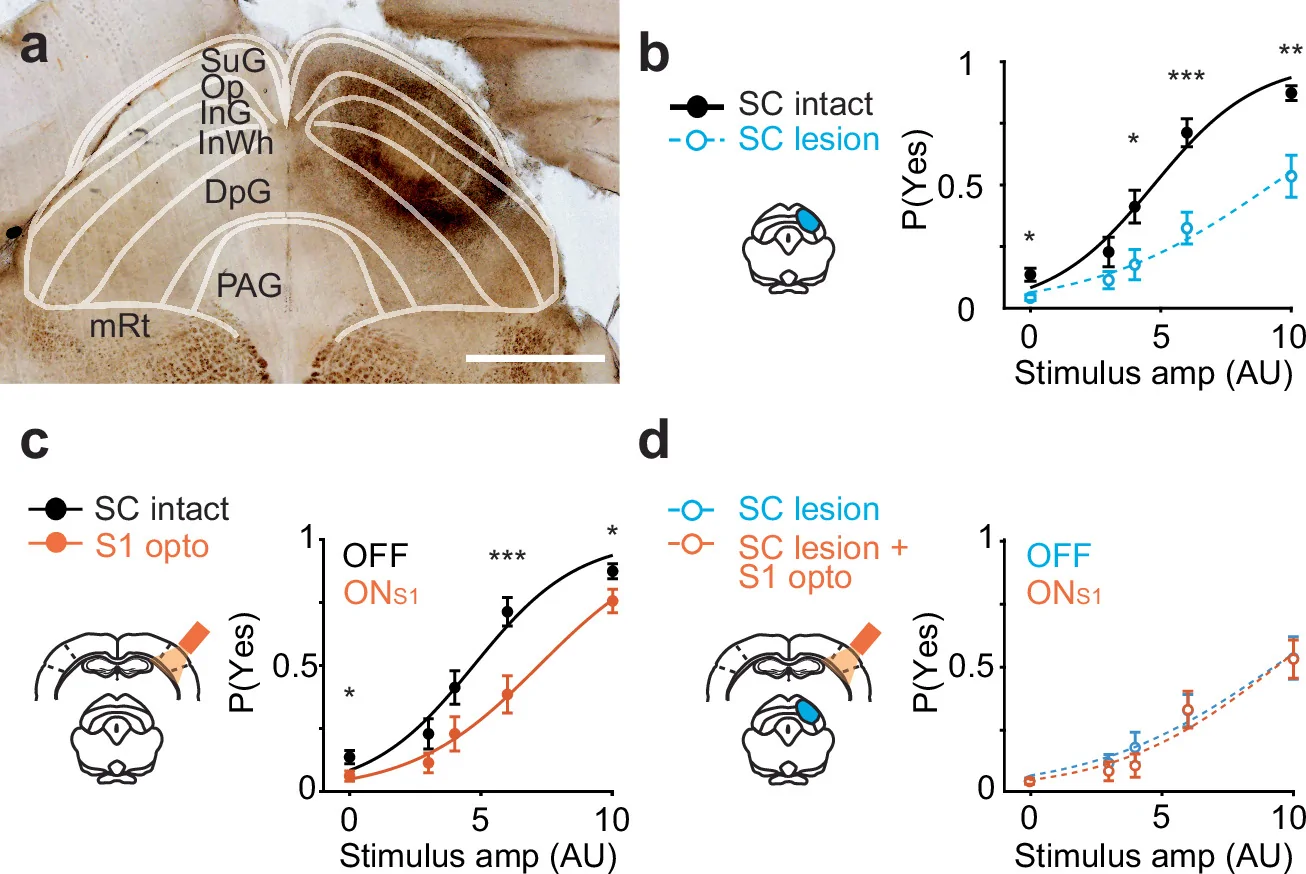
S1 inactivation has minimal behavioral effects in the absence of SC. **a** Brightfield image of an example SC lesion. Lesions were targeted by laser ablation in contralateral SC. **b** Probability of Yes response (P(Yes) before (black, filled circles; 2 days pre- lesion) and after (cyan, open circles; 2 days post-lesion) SC lesions. **c** Pre-SC lesion performance (black, same as in **b**) vs. contralateral S1 inactivation (S1opto; orange) in the intact animal. **d** Post-SC lesion performance with SC lesion (cyan; laser-OFF, same as in **b**) and during S1 inactivation in SC-lesioned animals (orange; laser-ON). All data are presented as mean ± s.e.m. across 12 sessions from 6 mice (2 days prelesion and 2 days post-lesion per mouse). Linear mixed-effects models were used, with mouse as a random effect for all comparisons; session was included as an additional random effect for within-session laser-OFF vs. ON comparisons. For (**b**) pre- vs. post-SC lesion amps 0, 3, 4, 6, and 10: *p* = 0.017, 0.43, 0.011, 4.23 × 10^−^^5^, and 0.0041, respectively; for (**c**) OFF vs. ON comparisons *p* = 0.016, 0.18, 0.070, 4.06 × 10^−^^5^, and 0.048, respectively; and in (**d**) *p *= 1, 1, 0.61, 1, and 1, respectively. *P*-values were Bonferroni-corrected across stimulus amplitudes; * *p* < 0.05, **p < 0.01, *** *p* < 0.001. Scale bar: 1 mm. SC layer reference outlines in (**a**) and schematics in (**b**, **c**, **d**) adapted from Allen Reference Atlas – Mouse Brain, atlas.brain-map.org^72^. Source data are provided as a Source Data file.

S1 projects to several downstream targets, and prior work has shown that chemogenetic silencing of S1 axon terminals in either SC, striatum, or POm impaired detection behavior^11^. While our data revealed a strong correlation between changes in SC population activity and behavioral choice, the effects of S1 inactivation could be mediated by alternative pathways independent of the SC. To directly test whether the behavioral changes during S1 inactivation are SC-dependent, we combined S1 inactivation with SC lesions. SC_contra_ lesions were performed by laser ablation targeted to the C2 whisker-responsive region (Fig. 7a and Supplementary Fig. 9a). Lesions were calibrated to target the intermediate layers of SC while avoiding lateral SC (Supplementary Fig. 9a, b; average lesion volume: 1.23 mm^3 ^± 0.095 mm^3^; mean and s.e.m.; *n* = 6 mice).

We first assessed the behavioral effect of SC lesions alone. SC_contra_ lesions strongly impaired detection performance (Fig. 7b). Notably, SC lesions produced stronger impairments compared to Pitx2-Halo-mediated SC_contra_ optogenetic suppression, likely due to the larger extent of the lesion that spanned the superficial and intermediate layers (Supplementary Fig. 9a). In a few sessions, SC lesions resulted in strong impairments, leading to unstable psychometric fits, limiting reliable interpretation of bias and sensitivity parameters. Despite this, analysis of the remaining sessions showed significantly increased thresholds and a conservative shift in choice bias (Supplementary Fig. 9c, d). No significant changes in sensitivity were detected (Supplementary Fig. 9e).

While animals were significantly impaired after SC lesions, they retained some ability to perform the detection task at strong stimulus amplitudes (Fig. 7b), which could be mediated by the intact S1 pathway^15^. We next asked how S1 inactivation influences detection in the absence of SC. As a baseline, we confirmed that S1_contra_ inactivation reliably reduced detection probability prior to SC_contra_ lesions in this cohort (Fig. 7c), consistent with earlier results. Strikingly, following SC lesions, additional inactivation of S1 did not further impair detection performance (Fig. 7d). Together, these results suggest that behavioral effects of S1 inactivation depend on SC activity, with SC serving as a key downstream pathway through which S1 modulates detection behavior.

Taken together, these results demonstrate that the behavioral impairment associated with S1 inactivation is primarily due to a shift in perceptual decision bias, which is dependent on SC activity. Our results support a model in which S1 contributes to tactile detection not only by locally encoding sensory information, but by enhancing sensory-evoked responses in SC, and modulating SC-dependent circuits that determine how tactile evidence is converted into perceptual decisions.

## Discussion

A common interpretation is that S1 inactivation impairs detection behavior due to the loss of sensory information encoded locally in S1. This is a reasonable view given that contralateral S1 is the primary cortical target for somatosensory input, and numerous studies have shown that inactivation of contralateral S1 increases the detection threshold for whisker stimuli^4,5,9,10,12,13,15^. However, several key results argue against a simple loss-of-sensory-information model. First, although contralateral S1 inactivation decreased detection probability as expected, ipsilateral S1 inactivation produced the opposite effect. Second, these opposite effects on detection probability also extended to no-stimulus trials, significantly decreasing or increasing false alarm rates depending on the hemisphere inactivated. Third, bilateral S1 inactivation led to no significant changes in detection threshold, unlike contra- or ipsilateral inactivation. Fourth, psychometric analysis revealed that these behavioral effects of S1 inactivation were more consistent with directional shifts in choice bias than with changes in sensitivity (Fig. 3). Finally, S1 inactivation decreased spontaneous and sensory-evoked neural activity in SC, and trial-by-trial choice was more reliably predicted by changes in population activity in SC than by decreased sensory responses in S1 (Fig. 6f). Thus, the behavioral effects of S1 inactivation cannot be explained simply by decreased sensory evidence encoded in S1.

An alternative possibility is that S1 and SC function as parallel sensory pathways with different neurometric thresholds. In this model, S1 serves as the default, low-threshold pathway, but when S1 is inactivated, the SC supports residual, higher-threshold detection behavior. Consistent with this view, sensory-evoked responses in S1 were larger than in SC for near-threshold stimuli (Fig. 4f, g), and prior work suggests that in the absence of S1, SC can support detection of salient, but not weak stimuli^15^. However, several key results argue against a dual-threshold model. First, SC lesions impaired detection behavior even when S1 remained intact^15^ (Fig. 7b). Second, in the absence of SC activity, additional S1 inactivation did not impair the residual detection performance (Fig. 7d). Thus, although S1 and SC both carry tactile stimulus information, the behavioral effects of S1 inactivation are more consistent with a lateralized shift in decision bias via changes in SC activity, rather than the unmasking of a backup SC pathway when S1 activity is lost.

What are the roles of S1 and SC during detection behavior? Our results are consistent with a model in which S1 contributes to tactile detection not only by enhancing sensitivity for weak stimuli, but also by modulating SC to shape action selection. At the neuronal level, S1_contra_ inactivation decreased spontaneous and sensory-evoked activity in downstream SC_contra_, potentially reducing the likelihood that sensory input reaches the threshold for a detection response.

How do changes in SC activity during S1 inactivation lead to choice bias? S1 inactivation-induced choice bias was more consistent with a perceptual bias rather than a motor bias. The reversed motor contingency experiments (Supplementary Fig. 6) suggest that unilateral S1 inactivation biased perceptual decisions (Yes/No) independent of motor action (lick right/left). In contrast, unilateral inactivation of lateral SC is known to directly impair directional licking output^21,45^, consistent with the strong directional bias we observed with Pitx2-Halo-mediated SC inactivation (Fig. 2). Given the current data, we cannot rule out whether S1 inactivation also alters premotor/motor output neurons outside of the recording site in lateral SC. Notably, whisker S1 axons project not only to the stimulus-responsive neurons in the upper region of the intermediate layers, but also the lower intermediate and deep SC layers where premotor/output neurons reside (Supplementary Fig. 3). One possibility is that, in addition to suppressing stimulus-evoked responses in SC, S1 inactivation directly or indirectly affects the premotor/output neurons to bias action selection. Thus, although the dominant effect of S1 inactivation was a shift in perceptual bias, we cannot rule out a weaker motor bias effect that may have been masked by the larger perceptual bias observed during unilateral S1 inactivation.

Although Pitx2-Halo-mediated SC inactivation experiments produced strong shifts in choice bias, we did not observe changes in sensitivity. This is in contrast to previous work in mice performing a visual change detection task, in which contralateral SC inactivation decreased sensitivity^20^. Given the incomplete overlap between Pitx2-Halo and S1 projection areas noted above, Pitx2-Halo likely labels a limited subset of excitatory neurons in the intermediate SC, excluding a large portion of tactile sensory SC neurons. Contralateral SC lesions did not reveal clear changes in sensitivity, although these effects were difficult to interpret given the severe behavioral impairment and stronger effects on choice bias. Future experiments targeting SC neurons that receive ascending trigeminal brainstem sensory input would help determine whether distinct SC neurons contribute separately to tactile sensitivity and decision bias.

In a Go/No-go detection task that does not require lateralized motor output, S1_contra_ inactivation also altered choice bias rather than sensitivity (Supplementary Fig. 6k–n). An interesting prediction is that ipsilateral S1 inactivation would also bias detection behavior in the Go/No-go paradigm. Future experiments are needed to test this possibility.

Unilateral S1 inactivation may shift choice bias by altering the interhemispheric balance downstream in SC. In support of this idea, simultaneous bilateral SC recordings in a limited dataset revealed that S1_contra_ inactivation not only decreased activity in SC_contra_, but concurrently increased baseline activity in the opposite SC_ipsi_ (Supplementary Fig. 10). This effect is consistent with models of mutual inhibition across SC^44^. Moreover, recent work in primates performing a visual attention task suggested that perceptual choices can be predicted by relative activity between the two SC hemispheres^24^. Cortical inactivation leading to an interhemispheric imbalance is reminiscent of the classic Sprague effect, in which unilateral lesions of the visual cortex led to hemineglect, which could be remediated by an additional lesion in the opposite SC hemisphere or collicular commissure^57^. Based on an interhemispheric imbalance model, we speculate that unilateral S1 inactivation may induce an offset between SC hemispheres, altering the amount of sensory evidence required to reach the threshold for a detection response.

Although our results implicate SC as essential for S1 inactivation-induced bias shifts, they do not distinguish whether S1 modulates SC via direct S1-SC projections or indirectly via higher-order sensory or motor cortical areas and subcortical areas such as the basal ganglia, thalamus, and the trigeminal brainstem nuclei. Prior work has demonstrated that suppressing activity in S1 axon terminals in either SC, POm, or dorsal striatum can impair detection behavior^11^. In addition, the cortico-basal ganglia circuits have been proposed to regulate decision thresholds by influencing SC output, providing an indirect pathway by which S1 could modulate sensory-guided reports in SC^58^.

Given the convergence of multisensory inputs and capacity for rapid sensorimotor transformations, the SC is ideally suited for the rapid detection of salient stimuli in the environment. However, its relatively limited spatiotemporal receptive fields and strong neural adaptation constrain both the spatial and temporal resolution of sensory integration^46,59,60^. Such properties make the SC ideally suited for rapid detection, but poorly suited for finer discrimination, sensory evidence accumulation, or complex perceptual judgments. In these contexts, primary and higher-order sensory cortices likely play a more indispensable role, contributing not only to bias adjustments but also to the fidelity of sensory encoding, which is essential for more demanding tasks^61^. For example, in primates, attentional modulation of V4 reflects changes in sensitivity rather than bias^61^, and recent work in primate SC similarly found that attention-related shifts in SC reflect sensitivity and motor bias, but not perceptual bias^62^.

Together, our results suggest that S1 not only enhances sensory responses in SC but also modulates SC to shape decision criteria that guide action selection. Such a mechanism could enable animals to dynamically shift biases in response to context, including reward contingencies, attentional state, and ongoing task demands. For example, adopting a liberal choice bias in response to detecting a looming predator can be life-saving by increasing detection response probability at the cost of a higher false alarm rate. These results may also help explain how acute cortical perturbations can lead to apparent sensory impairments during detection behaviors, even when the primary sensory cortex is not strictly required for simple detection behaviors^6,9,10,12,15^. Rather than merely decreasing sensory information, cortical perturbation may disrupt the balance of downstream decision circuits. Similar principles likely extend beyond the somatosensory system, representing a general mechanism in which cortical areas can flexibly bias subcortical decision circuits.

## Methods

### Animals

All animal procedures complied with the National Institutes of Health Guide for the Care and Use of Laboratory Animals and were approved by the Institutional Animal Care and Use Committee at Carnegie Mellon University. Animals were housed under a 12-h light-dark cycle at an ambient temperature of 68-79^o^ F and 30–70% humidity. For S1 inactivation experiments, Emx1-IRES-Cre^35^ knock-in mice (Jackson Laboratories, stock #005628) were crossed to Rosa-lox-stop-lox (RSL)-eNpHR3.0/eYFP mice (Ai39, JAX, stock# 006364), which express halorhodopsin (eNpHR3.0) after excision of a stop cassette by Cre recombinase (Emx1-Halo, *n* = 25 mice). Pitx2–Cre^63^ mice (Pitx2tm4(cre)Jfm, provided by J. Martin and N. Kurpios) were crossed to RSL-H2B–GFP mice^64^ (provided by J. Huang) to express GFP for visualization of Cre-positive neurons (Pitx2-GFP, *n* = 1, histology only) or with Ai39 to express halorhodopsin (Pitx2-Halo, *n* = 7 mice) in a subset of cells in the intermediate layer of SC. A total of 33 mice (21 males, 12 females, ages P90 to P330, complete list in Source Data file) were used in the study. All lines were maintained on a C57BL/6 J background. Optogenetic experiments used mice that were heterozygous for the transgenes. No significant sex differences were found in learning rate, baseline performance, or the effect of S1 inactivation between trained males and females. Data from males and females were therefore pooled for analyses. All animals used in the study are listed in the Supplementary Data 1.

### Behavioral setup

Behavioral events were controlled by a microcontroller (Arduino). Water-restricted animals were head-fixed, and a ~ 4 mm stainless steel tube (McMasterCarr #8988K79) was temporarily glued (Elmer’s) to the right C2 whisker, 4 to 8 mm from the base of the follicle. The remaining whiskers were trimmed to facilitate access to the C2 whisker. A single, Gaussian pulse was delivered to an electromagnetic coil (Erse audio) using a 300 W audio amplifier at variable amplitudes, resulting in an electromagnetic pulse that deflected the whisker downwards (~ 25 ms from baseline to peak deflection). At the start of each test session, the stimulus amplitude was calibrated with high-speed videography (Basler, 250–500 fps), where the maximum amplitude (amp) 10 was calibrated to ~ 40^o^, equivalent to an angular velocity of ~ 2100^o^ /s (Supplementary Fig. 1c, d). This maximum whisker velocity is in the range that mice used when voluntarily whisking to detect an object in a previously reported detection task^9^. Two water ports were placed ~ 0.5 cm apart, and licks were detected with an infrared diode-phototransistor (Sparkfun QRD1114). The passive whisker stimulus design enabled precise control over stimulus timing and amplitude, circumventing the confound that S1 inactivation can reduce overall whisking vigor and inadvertently alter the experienced stimulus strength^9^. We implemented a forced-choice (Yes/No) paradigm that requires an overt response on every trial, avoiding the ambiguity inherent to Go/No-go tasks, where an absence of response for ‘No-go’ trials could reflect motor or motivational deficits.

### Whisker-mediated tactile detection behavior

#### Training

Adult mice (P181 ± 47 days, mean and SD; 58% male) were implanted with custom-designed headplates with dental cement (Metabond) under isoflurane anesthesia. After at least 3 days of recovery, mice were water restricted to begin training in stages as previously described^2,3^: (1) Habitation: mice were gradually habituated to handling and head-fixation in light-sealed behavior boxes (1-2 days). (2) Dual lick-port training: head-fixed mice learned to lick the two ports equally, by alternating the rewarded port (4 d). (3) Simple task: mice then underwent stimulus association training. Trials started with a 50 ms start cue, followed by a stimulus 700 ms from trial start (amplitude 0 or 10 only), where the right port was associated with stimulation of the animal’s right C2 whisker and the left port with no stimulus (2-4 d). During training sessions, a response cue tone was delivered 300 ms from stimulus onset, after which lick responses were registered. Mice received a drop of water reward for correct responses, or a 1-s auditory white noise stimulus as punishment for incorrect responses. Learning was defined by a criterion of d’ > 1.5 for 3 consecutive sessions (~ 75% correct). Mice reached the learning criterion after a median of 9 training sessions (Supplementary Fig. 1a, b). (4) Full task: each session started with 50 warmup trials, during which alternating trials of stimulus amplitude 0 or 10 were delivered to habituate animals to the positions of the two lick ports. Once animals passed the warmup block, intermediate stimulus amplitudes were added. Each stimulus (amplitudes 0, 3, 4, 6, or 10 AU) was delivered with a probability of 0.4, 0.1, 0.1, 0.2, and 0.2, respectively. Probabilities and amplitudes were determined empirically to produce reliable threshold measurements and stable performance across sessions and animals. To prevent animals from adopting a Go/No-go strategy in which they licked only the right port, if the animal failed to respond to a single trial type for 4 consecutive trials, a correction block was triggered, in which animals were required to correctly respond for 3 consecutive trials of the non-preferred trial type before continuing the session. Mice were trained until behavioral performance stabilized before participating in experiments. Warmup and correction block trials were excluded from all analyses.

#### Full task

For the final Yes/No forced choice paradigm used for experiments, a 50 ms auditory tone signaled the start of each trial, and a stimulus (amplitude 0, 3, 4, 6, or 10 AU) was delivered at 700 ms from trial start. After a 300-ms delay period, the response window opened for 2 s, and licking the correct water port delivered a drop of water reward (Fig. 1c). The next trial began after an inter-trial interval (ITI) of 2-3 s. For behavior-only sessions, lick timing was defined as the first registered lick during the response window, and should be interpreted as the latency of the first registered lick rather than a precise measure of reaction time. For behavior during neural recordings, analog lick signals were recorded with the neural data through the OpenEphys data acquisition system, and true lick latencies were acquired. Except for the start cue tone, all auditory cues (‘go’ cue for response window and punishment sound) were removed to avoid auditory responses in SC recordings. For the Go/No-go version of the task (Supplementary Fig. 6k–n), animals were trained to lick a single lick port to report stimulus detection (Go), or do nothing if no stimulus was detected (No-go). If the animal licked within 500 ms before the trial start, the trial was aborted.

#### Intrinsic signal optical imaging to map C2 barrel in S1

Functional mapping of the C2 barrel was performed as previously described^9^. Under isoflurane anesthesia, the skull over S1 was thinned (~ 4 mm window centered around stereotactic coordinates AP −1.5, ML 3.5 from Bregma) to clearly visualize the surface vasculature, and sealed with a thin layer of cyanoacrylate. With a camera positioned above the mouse (Teledyne FLIR), a 530 nm LED (Thorlabs) was used to acquire an image of the vasculature, and a 625 nm LED was used to acquire the intrinsic signal. The C2 whisker was stimulated at 10 Hz with a piezo stimulator, and the signal was averaged over 20–30 trials. The signal was then mapped onto the vasculature image. Imaging and analyses were performed using custom-written software in MATLAB.

#### Optogenetic inactivation

S1 inactivation in Emx1-Halo mice was performed as previously described^9^. A 594 nm laser (Coherent) was delivered via a 200-um diameter optic fiber (0.39 NA, Thorlabs) positioned over a thinned skull, with the fiber tip centered at the mapped C2 barrel (35 mW from fiber tip). Based on our previous characterization of S1 photoinhibition in Emx1-Halo mice, neurons within a 1-mm radius can be effectively silenced, which fully encompasses the center C2 barrel and extends beyond the adjacent whisker barrels in S1^9^. Laser-ON trials were randomly interleaved in 50% of trials. The laser turned on 50 ms before the trial start cue to ensure maximum inactivation by trial start, and remained on for the full duration of the trial. Before the first test session, animals were habituated to the laser without inactivation, with the optic fiber positioned above the mouse’s head (> 10 cm) with blackout tape covering the skull, illuminating the behavioral setup but not affecting neural activity. This also served as a negative control to rule out changes in behavior due to visibility or startle effects during laser-ON trials (Supplementary Fig. 1e–i). For experiments directly comparing unilateral vs. bilateral S1 (*n* = 6 Emx1-Halo mice, 14 sessions) or SC inactivation (*n* = 6 Pitx2-Halo mice, 21 sessions tested), the laser beam was split and directed into 2 optic fiber ports, and calibrated to maintain equal output power (30 mW) from each fiber. S1 inactivation was targeted to the center of the mapped C2 barrel coordinates.

For SC inactivation, 593 nm laser illumination was delivered via implanted optic ferrules (AP − 3.7, ML 1.7, from Bregma, 1.6-1.9 mm depth from pial surface, inserted at a 20^o^ angle from the vertical plane). The laser output from each fiber was balanced and calibrated (30 mW output from each 200 µm optic fiber tip, 0.39 NA) to maintain balanced power levels between hemispheres and across animals and sessions. We used Pitx2-Cre knock-in mice^38,63^ to drive expression of eNpHR3.0 (Ai39 reporter mice) (Pitx2-Halo) in excitatory neurons of the SC intermediate layers (SGI). Pitx2-Cre is expressed in a functionally heterogeneous, glutamatergic population that forms modular premotor units that tile the SGI and accounts for around half of local excitatory neurons^38^. Inactivation of excitatory neurons using Pitx2-Halo was chosen rather than excitation of inhibitory neurons, which have been shown to generate contralateral choice biases via inter-collicular inhibition^44^, potentially confounding interpretations about local SC computations. Pitx2-Cre targeting avoids direct activation of long-range inhibitory neurons, many of which project to and from SC.

#### SC lesions

SC_contra_ was lesioned by laser-mediated ablation, calibrated to target the superficial to intermediate layers of SC while minimizing damage to neighboring structures. Mice were deeply anesthetized under isoflurane, and a large craniotomy was made over SC. To target contralateral SC, an optic fiber (200 µm, 0.39NA) was sequentially inserted at 3 locations (AP − 3.7; ML 1, 1.7, 2.2 mm from Bregma) at different depths (Z − 1.14, − 1.5, − 1.75 mm) from the pial surface. At each location, a 432 nm laser (Laserglow) was applied for 5–10 min at 100 mW. The craniotomy was then sealed with Vetbond (3 M) and covered with dental cement (Stoelting).

Due to variability in SC lesion sizes and locations, animals were excluded from analysis when the following histological criteria applied: (i) small lesions that did not encompass at least 300 µm of the expected C2-responsive area (Supplementary Fig. 6 and Supplementary Fig. 9); (ii) lesions that extended into lateral SC (known to control lateralized licking movements^21,43,45^); or (iii) lesions that extended beyond the ventral SC border, damaging the midbrain reticular nucleus or periaqueductal gray (PAG). In addition to histological analysis, several behavioral criteria were also used for exclusion: after SC_contra_ lesions, animals were tested for lateralized lick impairments starting 24 h post-lesion. At the start of each behavioral session, mice were required to alternate licking the right and left water ports for 10 trials to receive water rewards. If animals failed to complete this warm-up period within a few minutes, the session was aborted. Only mice that demonstrated the ability to lick in both directions were tested on the full task for 3 consecutive sessions. Immediately after surgery, some animals exhibited mild ipsiversive circling behavior, a hallmark of unilateral SC lesions, which typically subsided within 1-2 h post-surgery. In a few cases, circling behavior was severe and lasted longer, which also corresponded to larger SC lesions extending into the deep SC layers and the medial reticular nucleus. Such animals were excluded from further testing. Histology images from each lesioned animal included in the study are shown in Supplementary Fig. 9.

#### Histology

Mice were perfused with phosphate buffer (PB) followed by 4% PFA in PB. Brains were extracted and post-fixed in 4% PFA solution overnight. The tissue was coronally sectioned into 100-µm-thick slices using a vibratome (Leica) or freezing microtome (Epredia). Sections were mounted and imaged using a brightfield or epifluorescence microscope (Olympus, 4 x objective). Open-source software (github.com/petersaj/AP_histology) was used to align histology sections to the Allen Brain 3D Common Coordinate Framework^65^.

#### Electrophysiology

Topographically aligned C2-responsive areas in S1 and SC were simultaneously recorded during behavior. Under isoflurane anesthesia, a small craniotomy was made over the target area, and silicone gel (Dow) was applied to seal the craniotomy. Between recording sessions, craniotomies were additionally sealed with Kwik-Cast (World Precision Instruments) and reapplied between sessions. To confirm probe locations post-mortem, we applied lipophilic dyes (DiI or DiD, Thermo Fisher Scientific) onto the back of the probes before each recording and histologically confirmed the recording sites. Probe tracts were mapped using Neuropixels trajectory explorer (https://github.com/petersaj/neuropixels_trajectory_explorer) and reconstructed into a 3D model for visualization (Supplementary Fig. 6).

For S1, a linear electrode array (Cambridge Neurotech, 64 channels, 1250 µm span, single shank) was inserted into the functionally mapped C2 barrel (see intrinsic optical signal imaging), at a 30^o^ angle to a depth of 1.2 mm from the pial surface to span the full cortical depth. For SC, a two-shank silicon array probe (Cambridge Neurotech 64 channels, 750 µm vertical span, 250 µm separation between 2 shanks) was inserted at a 20^o^ angle. On the first day of recording, probes were targeted to 3.7 mm posterior, 1.7 mm lateral from Bregma; 2.4-2.5 mm from the pial surface. The recording target was identified by marching the insertion location of the electrode until robust responses to C2-whisker stimulation were identified in both shanks. Acute recordings were acquired over 3-4 consecutive days. Data were sampled at 30 kHz (OpenEphys acquisition board and software) and band-pass filtered (300–6000 Hz). Spikes were sorted offline with Kilosort 2.5 or 3.0^66^ and single units were manually curated using the open-source Python library Phy (https://github.com/cortex-lab/phy.git). For each unit, spike trains were aligned to trial onset and binned into 1 ms bins. Only well-isolated single units were used for analysis. Sessions with fewer than 10 units in either area were excluded from analysis. Experiments with no significant stimulus-responsive units in either area were deemed to be mis-targeted and were excluded. For all quantification and statistical analyses, firing rates were analyzed without temporal filtering. For visualization of peri-stimulus time histograms (PSTHs), spike data were smoothed with a Gaussian kernel (sigma = 3 ms). A total of 423 S1 single units and 778 units were recorded across 17 sessions from 6 mice. Unit identities, recording sessions, animal IDs, and the number of units recorded in each session are provided in Supplementary Data 2.

#### Whisker tracking

Whisker angles were quantified using high-speed videography (Basler, 250–500 fps) under infrared illumination for each session. DeepLabCut^67^ was used to track whisker movements for each session. The maximum angle deflection during the stimulus window (100 ms after stimulus onset) was determined for each trial, and did not differ between laser-OFF and ON trials (Supplementary Fig. 1c, d).

### Quantification and statistics

#### Behavioral analysis

The probability of a Yes response (P(Yes)) was calculated for each stimulus amplitude (total Yes responses/total valid trials at each amplitude). ‘False alarm’ rate is the probability of a Yes response to no stimulus (amplitude = 0) trials. ‘Hit rate’ is the probability of a Yes response for all trials with stimulus > 0. The percent correct was [hit rate + (1- false alarm rate)]/2, equally weighing stimulus and no-stimulus trials. For figure display only, P(Yes) plots were fitted with logistic fits of the average probability of a Yes response at each stimulus amplitude.

To quantify psychometric performance, we used logistic regression analysis:

1 $${{{\rm{logit}}}}({{{\rm{P}}}}({{{\rm{Yes}}}}))={\beta }_{0}+{\beta }_{1}\,{{{\rm{s}}}}$$

where P(Yes) is the probability of a Yes response; *β*_0_ is the intercept (bias); *β*_1_ is the slope (sensitivity) and ***s*** is the stimulus amplitude. A decrease in bias indicates a more conservative response strategy (less likely to respond Yes), while an increase reflects a more liberal decision strategy (more likely to respond Yes), regardless of the stimulus strength. Detection threshold is equivalent to the stimulus amplitude needed to respond Yes 50% of the time (-*β*_0_/*β*_1_). Bias measurements were closely correlated with the false alarm rates and consistently reflected behavioral shifts induced by inactivation. In contrast, the sensitivity parameter *β*_1_ was variable and inconsistent, as impaired performance can be accompanied by increases or decreases in slope (simulated in Supplementary Fig. 2 for clarification). This made sensitivity estimates unreliable as a measure of perceptual sensitivity. To address this, we complemented logistic model analysis with model-free measurements of performance, including the raw probability of detection values by stimulus amplitude (p(Yes) curves), which explicitly incorporates the observed false alarm rate (p(Yes | amp 0)). We also derived thresholds from the raw data by interpolation, quantifying the amplitude at which the detection probability crossed 0.5. These interpolated thresholds were highly consistent with model-derived thresholds (-*β*_0_/*β*_1_), and provided confirmation that threshold measurements were reliable. Since both increases and decreases in slope can reflect impaired performance (Supplementary Fig. 2), average sensitivity measurements were difficult to interpret. We therefore focused analyses on detection threshold and bias shifts, which offered more robust and interpretable measures of behavioral change. Thus, we emphasized bias, false-alarm rates, and interpolated thresholds as stable measurements of decision criteria, while treating the model-derived sensitivity estimates with caution.

For each session, warmup trials, correction trials, and trials without a response were omitted from analysis. In addition, sessions were excluded if any stimulus amplitude contained no responded trials or if the full session had fewer than 100 responded trials, often due to early satiation. To ensure good performance, only sessions where OFF-trial performance d-prime (between amplitudes 0 vs. 10) > 1.5 were included (d’ = Z(Hit rate) – Z (False alarm rate), where Z is the inverse of the standard normal cumulative distribution function). Sessions that failed basic fit criteria (*p* ≥ 0.5 for logistic model fit) were omitted from model-based summaries (threshold, bias (intercept, β_o_), and sensitivity (slope, β_1_)), but were included in model-independent raw values (p(Yes), % correct, hit rate, false alarm rate, interpolated threshold).

#### Behavioral statistics

Analyses were performed using custom MATLAB scripts. Normality was tested using the Shapiro-Wilk test. Statistical significance is represented as *p < 0.05, **p < 0.01, ***p < 0.001, or n.s. (not significant, *p* ≥ 0.05). Bonferroni correction was applied for multiple comparisons. Unless otherwise noted, paired ON-OFF comparisons (Figs. 1–3) were performed using two-sided Wilcoxon signed-rank tests on session-level data. Because each subject contributed multiple sessions, these results were additionally confirmed using mixed-effects models that included laser (OFF/ON) as a fixed effect and random intercepts for mouse and session. For psychometric analyses, we first fit a two-parameter logistic function (Eq. (1)) by session and laser condition. We then compared session-level changes (ON-OFF) in bias (*β*₀), sensitivity (*β*_1_), and threshold (-*β*₀/ *β*_1_) using linear mixed-effects models with a random intercept for animal to account for multiple sessions within subject. Confirmatory mixed-effects model results are provided in the Source Data file.

#### Whisker stimulus-responsive units

Single units were classified as whisker stimulus-responsive if the average activity during the stimulus window (100 ms from stimulus onset) for amp 10 trials was significantly different (*p* < 0.05, two-sided paired *t* test) from the baseline activity during a 500 ms window before stimulus onset. While classification of excitatory (regular spiking, RS) and inhibitory (fast-spiking) cells can be performed by standard clustering analysis of spike waveforms in S1, SC neurons are not classifiable using established methods^68^. Thus, for comparing S1 and SC responses, we did not distinguish between RS and FS cells in either area.

#### Neural d-prime (d’)

To compare neural and behavioral discriminability, we first computed an ideal observer measure based on the area under the receiver operating characteristic (AUROC) for classifying amp 0 vs. each stimulus amplitude^69^. For neural data, sensory-evoked spike rates of stimulus-responsive neurons were quantified as mean firing rate in the 100 ms stimulus window, baseline-subtracted using the average firing rate during the 500 ms pre-stimulus window for each trial. For each unit and laser condition, AUROC was only computed when at least 5 trials were available for both amp 0 and each stimulus amplitude. After applying these criteria, neural d’ was calculated from 202 stimulus-responsive S1 neurons and 433 stimulus-responsive SC neurons across 17 recording sessions from 6 mice. To control for class-size imbalances, neural AUROC used balanced class counts between amp 0 and each stimulus by random subsampling, averaged over 50 resamples. AUROC values were then converted to d’ via the signal detection mapping:

2 $${{{{\rm{d}}}}}_{{\mbox{AUROC}}}^{{\prime} }=\sqrt{2}{\Phi }^{-1}\left({\mbox{AUROC}}\right)$$

where ${\Phi }^{-1}$ is the inverse cumulative distribution function of the standard normal distribution^70^. For neural data, the sign of d’ was set by the sign of the evoked-rate difference (amp s – amp 0). For behavioral data, trials without licking were excluded. Behavioral *d’* was calculated for each recording session (*n* = 17 sessions) from the hit and false alarm rates: ${{{{\rm{d}}}}}_{{\mbox{behavior}}}^{{\prime} }={\Phi }^{-1}\left({\mbox{pHit}}\right)-{\Phi }^{-1}\left({\mbox{pFA}}\right)$. Behavioral AUROC was then obtained from d’ as AUROC = $\Phi (\frac{{d}^{{\prime} }}{\sqrt{2}})$. Behavioral d’ values were averaged across 17 behavioral sessions corresponding to the neural recording sessions.

#### Mean squared error (ΔMSE)

To quantify how well the behavioral change produced by S1 inactivation was captured by the sensory-evoked neural activity in each area, we calculated the AUROC-derived Δd’ = d’_ON_ – d’_OFF_ at each amplitude for each behavioral session. For S1, SC, and behavior, Δd’ was calculated from the corresponding across-session mean AUROC-derived d’ curves for each area (*n* = 17 recorded sessions from 6 mice). To focus on the shape of the inactivation-induced change across stimulus amplitudes rather than the overall gain or offset, each Δd’ curve was min-max normalized across stimulus amplitudes:

3 $$\widetilde{\Delta {d}^{{\prime} }}\left(k\right)=\frac{{\Delta d}^{{\prime} }\left(k\right)-{\min }_{{{{\rm{j}}}}}\Delta {d}^{{\prime} }\left(j\right)}{{\max }_{j}\,\Delta {d}^{{\prime} }\left({{{\rm{j}}}}\right)-{\min }_{{{{\rm{j}}}}}\,\Delta {d}^{{\prime} }\left(j\right)}\,$$

We then quantified the shape mismatch between neural and behavioral Δd’ curves by the mean squared error across amplitudes:

4 $$\Delta {{{\rm{MSE}}}}=\frac{1}{K}{\sum}_{k=1}^{K}{\left(\widetilde{\Delta {d}_{{\mbox{neural}}}^{{\prime} }}\left({{{\rm{k}}}}\right)-\widetilde{\Delta {d}_{{\mbox{behavior}}}^{{\prime} }}\left(k\right)\right)}^{2}$$

where Κ is the number of non-zero stimulus amplitudes included. A lower ΔMSE indicates a closer match in the pattern of inactivation-induced changes between neural and behavioral d’ curves, independent of absolute scale and offset.

#### Optogenetic modulation index (OMI)

For each single unit, the average firing rate within the stimulus window (100 ms window after stimulus onset) at near-threshold amplitude 6 was compared for laser-OFF vs. ON conditions. For the OMI of spontaneous activity, a 500 ms window before stimulus onset was used. The optogenetic modulation index was calculated as the difference divided by the sum of the firing rates for OFF vs. ON trials.

5 $${{{\rm{OMI}}}}=({{{{\rm{FR}}}}}_{{{{\rm{ON}}}}}-{{{{\rm{FR}}}}}_{{{{\rm{OFF}}}}})/({{{{\rm{FR}}}}}_{{{{\rm{ON}}}}}+{{{{\rm{FR}}}}}_{{{{\rm{OFF}}}}})$$

where FR is firing rate. Positive values indicate increased activity, while negative values indicate decreased activity. For each unit, significant modulation was determined using a two-sided unpaired *t* test across trials, and *p* < 0.05 was used to identify units with significant modulation.

#### Population decoding analysis

Decoding analyses were performed using custom MATLAB scripts with Statistics and Machine Learning Toolbox and Neural Decoding Toolbox^71^. Linear support vector machines (SVM) were trained to classify stimulus presence vs. absence of stimulus, or behavioral choice (lick right vs. lick left to stimulus). For both stimulus and choice decoding, amplitude 6 was chosen because it represented the near-threshold stimulus where lick right vs. left trials were most balanced. Recordings were performed simultaneously in S1 and SC per session, but decoding was performed using pseudopopulations pooled within each area across recording sessions because the number of eligible simultaneously recorded units was limited and highly variable across individual sessions, particularly for laser-ON conditions. Neural data were binned in 50 ms sliding windows, stepped by 25 ms. To minimize activity from licking or motor preparation, analysis was restricted to a 100 ms window starting at stimulus onset. Units were eligible if they had at least 5 trials per class. For each analysis, the number of units per resample run was downsampled to the minimum number of eligible units across S1 and SC. To facilitate comparison across decoding analyses, the number of units was matched across stimulus and choice decoding analyses (*n* = 282 for OFF only analyses (Fig. 4h, i); *n* = 119 for OFF vs. ON comparisons (Fig. 6e, f)). For cross-condition generalized decoding analysis across OFF and ON trials, classifiers were trained on OFF trials and tested on held-out OFF or ON trials. Stimulus decoding was balanced by lick direction. Units were sampled without replacement per resample run, and trials were sampled without replacement per unit and class. Decoding was repeated over 200 Monte Carlo resampling runs. Neural activity was z-scored, and decoding accuracy was evaluated with 5-fold cross-validation. To assess significance above chance, 1000 shuffle-label permutations were performed to generate a null distribution of decoding accuracies for each time bin. Empirical p-values were calculated by comparing observed accuracy with the shuffled-label distribution and corrected across time bins using the Benjamini-Hochberg false discovery rate (FDR, indicated as # *p* < 0.05; ## *p* < 0.01; ### *p* < 0.001 in Fig. 4h, i, Fig. 6e, f). For comparing decoding accuracies between areas or laser conditions, two-sided session block permutation tests were used. For S1 vs. SC comparisons, area labels were exchanged within matched simultaneous recording sessions; for OFF vs. ON, condition labels were exchanged within session for each area. The difference in mean decoding accuracy across the analysis window was compared with the permutation distribution. Tests used 1000 permutations and 20 pseudopopulation resampling runs per permutation. For time-resolved comparisons, bin-wise *p*-values were corrected using the Benjamini–Hochberg FDR.

### Reporting summary

Further information on research design is available in the Nature Portfolio Reporting Summary linked to this article.

## Supplementary information

Supplementary Information

Description of Additional Supplementary Files

Supplementary Data 1

Supplementary Data 2

Reporting Summary

Transparent Peer Review file

## Source data

Source data

## Data Availability

Behavioral and electrophysiology data generated in this study have been deposited in the Zenodo database (https://doi.org/10.5281/zenodo.22119217). Source data are provided in this paper.

## Code availability

Custom MATLAB scripts used for analysis in this study have been deposited in the Zenodo database (https://doi.org/10.5281/zenodo.22117824).
