# Supplementary material for: Somatosensory cortex shapes perceptual decision bias via the superior colliculus
Source: Nat Commun. 2026 Oct 10;17:11515. doi: 10.1038/s41467-026-78331-0 (PMC13745596; doi:10.1038/s41467-026-78331-0)
Supplement: Supplementary file 1 [file 41467_2026_78331_MOESM1_ESM.pdf]

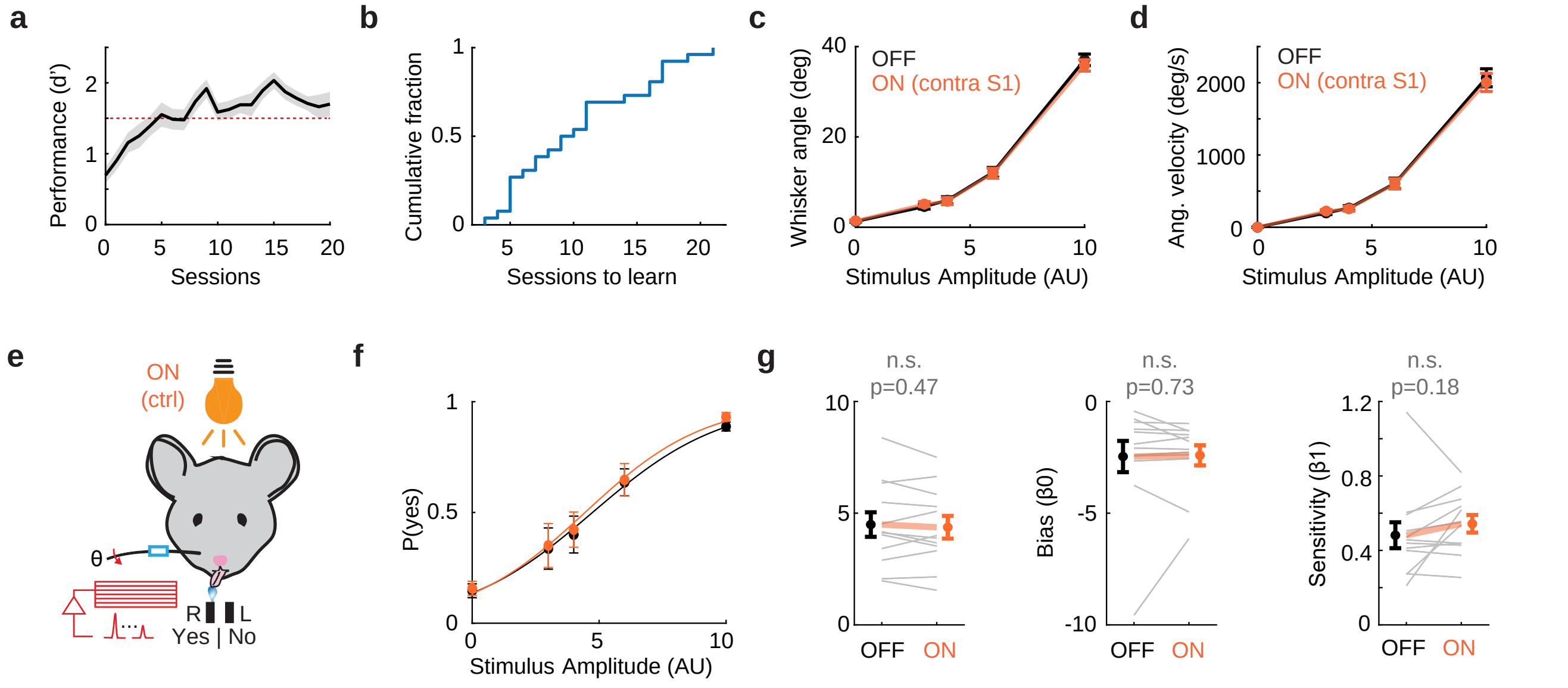

Supplementary Fig. 1 | Learning, whisker stimulus calibration, and negative controls.

a Performance measured by d-prime (d', amp 0 vs. amp 10) over learning sessions for animals used in this study (n=26 mice). Learning criterion was defined as d' >1.5 (red dotted line) over 3 consecutive sessions. **b** Cumulative plot of data in **a**. **c** Whisker deflection angles and **d**, equivalent angular velocities across stimulus amplitudes experiments in Fig.1 for laser OFF (black) vs. ON (orange) trials (n=22 sessions, 6 mice from Fig. 1). **e-i**, Laser-only negative controls for S1 inactivation experiments; **e** schematic of control experiments; 593-nm laser positioned above the animal so that light visible but the brain was not illuminated. **f**, Probability of detection, P(Yes), by stimulus amplitude. Solid lines are logistic fits to average responses at each amplitude for OFF vs. ON trials; n=11 mice. **g** Detection threshold; and **h** bias; **i** sensitivity for n=12 mice (n=6 mice from Fig. 1; n=6 mice from Fig.3. Error bars indicate mean ± s.e.m.; n.s., not significant; two-sided Wilcoxon signed rank test; Source data are provided as a Source Data file.

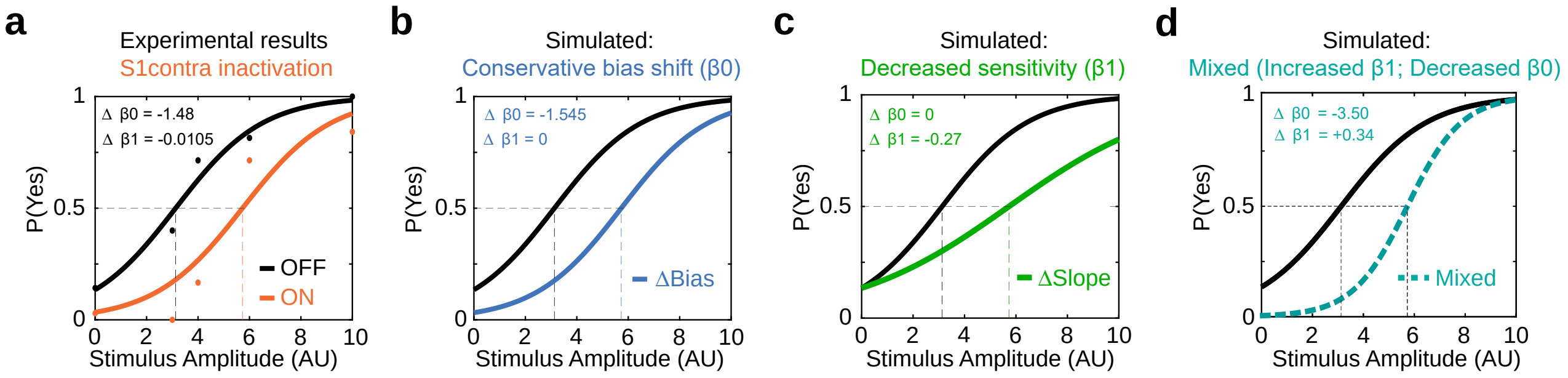

$$\mathrm{logit}(P(\mathrm{Yes})) = \beta_0 + \beta_1 \times \boldsymbol{s}$$

Supplementary Fig. 2 | Illustration of threshold changes due to sensitivity vs. bias.

Psychometric functions across stimulus amplitudes (***s***) were modeled using logistic regression to illustrate how bias (β_0) and sensitivity (β_1) parameters influence detection threshold. **a**, Example session from Fig. 1h showing empirical data (emp, filled dots) and logistic regression fits for laser OFF (black) and ON (orange) trials. OFF-trial empirical example data was used as baseline for all panels **a-d**. Threshold (dashed lines) is equivalent to the stimulus amplitude needed for a 50% 'yes' response rate (P(Yes) = 0.5, given by $-\beta_0/\beta_1$). **b**, Simulation of only changing the bias ($\Delta\beta_0$) to produce the same shift in detection threshold, which shifts the curve laterally without altering slope (blue). **c**, Simulation of a sensitivity change ($\Delta\beta_1$)) without bias change, which decreases the slope to reach the same threshold (green). **d**, Simulation showing how increases in slope (combined with bias shift) can also produce an increased threshold and impaired performance (dashed teal line). These examples illustrate how either bias or sensitivity can alter detection threshold; the empirical effect of $S1_{contra}$ inactivation is more consistent with a change in bias.

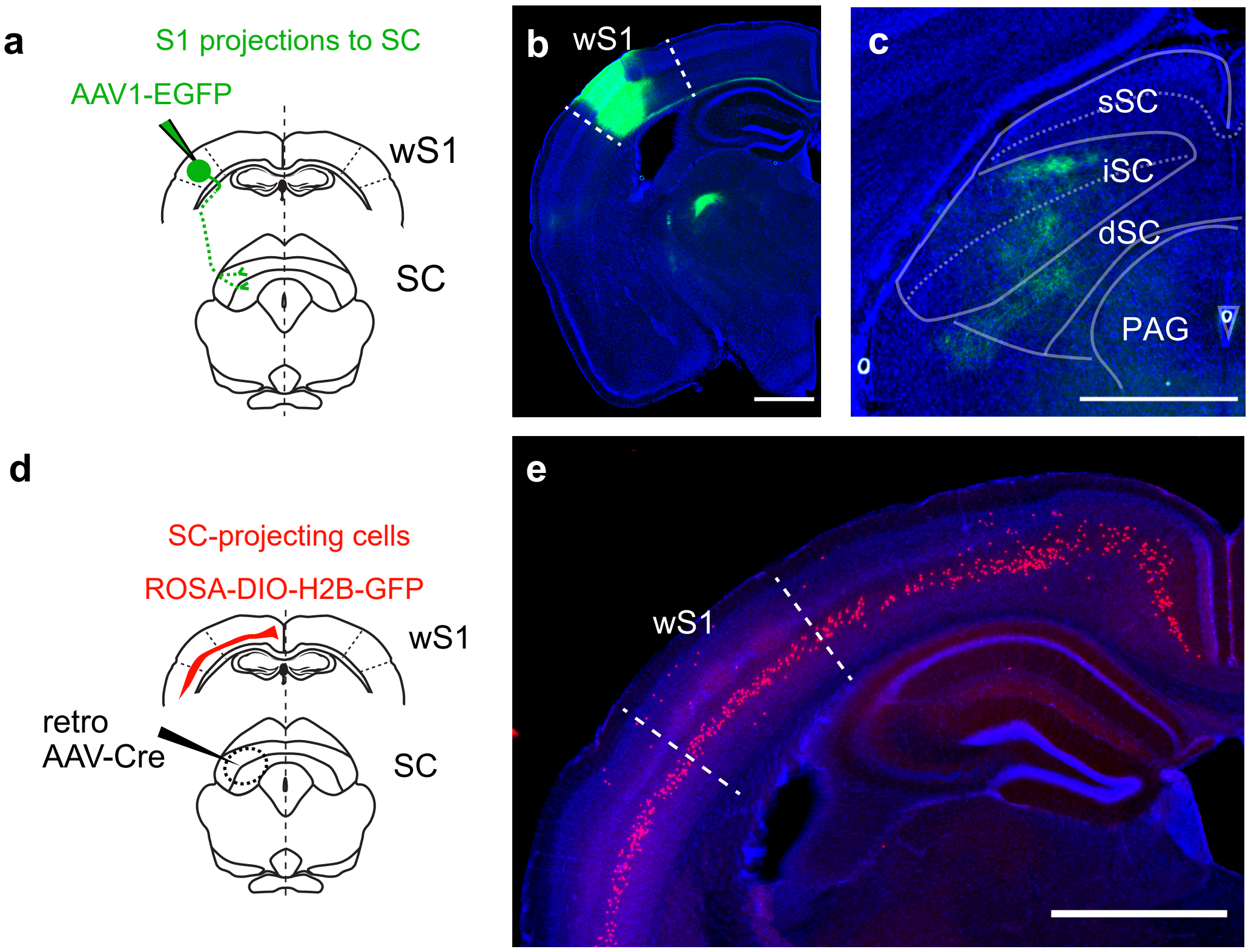

Supplementary Fig. 3 | Whisker S1 projects to intermediate and deep SC.

a Schematic of injection; **b** AAV1-EGFP was injected into the C2 barrel in whisker S1 (wS1) to visualize S1 axon projections to SC. **c** S1 axons are found in the upper and lower patches within the intermediate SC layers (iSC), as well as deep SC (dSC). No axons were found in the contralateral SC. **d** Schematic of retrograde AAV-Cre injection into SC to label S1 neuronal cell bodies that project to SC (H2B-GFP reporter mouse). **e** Retrogradely labeled cortical neurons are found primarily in layer 5b in wS1 and broadly across cortex (red). wS1: whisker S1; sSC: superficial SC; iSC: intermediate SC; dSC: deep SC layers. Scale bars: 1mm. Schematics of brains in **a** and **d** were adapted from Allen Reference Atlas – Mouse Brain, atlas.brain-map.org.

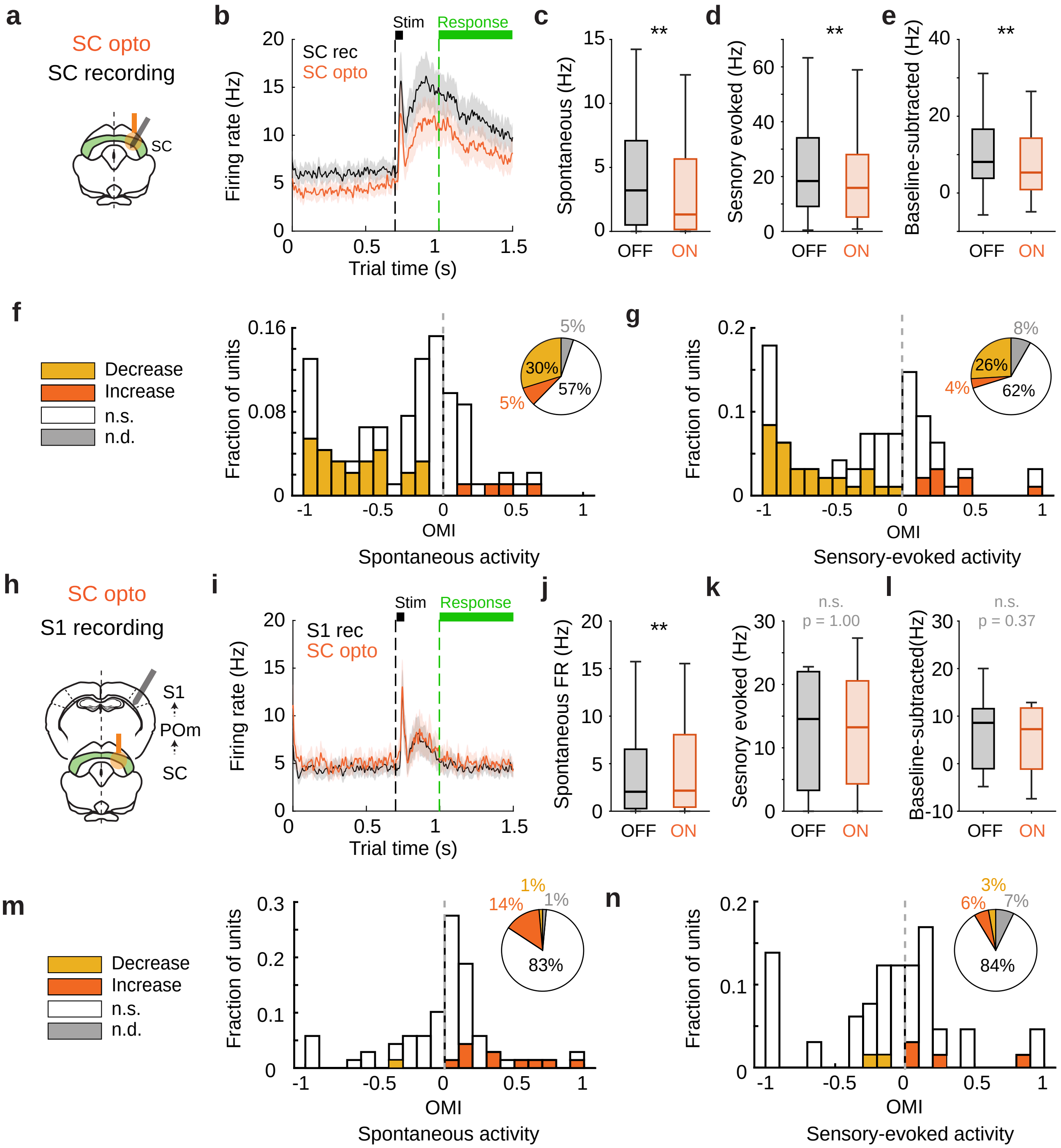

Supplementary Fig. 4 | SC and S1 recordings during inactivation of intermediate SC in Pitx2-Halo mice.

a-**g** SC recording during SC inactivation. **b** Peristimulus time histograms (PSTHs) for laser OFF (black) vs. laser ON trials (orange; n=100 SC units, 3 sessions, 2 Pitx2-Halo mice, mean ± s.e.m.). **c** Spontaneous firing rate (p=0.0052; n=100 units). **d** Sensory-evoked firing rate for whisker-responsive neurons at stimulus amp10 (p=0.00034; n=30 stimulus-responsive SC units); **e** baseline-subtracted sensory-evoked activity for same units in **d** (p=0.00015). **f** Population histogram of optogenetic modulation indices (OMI) for spontaneous activity (n=100 units) and **g** activity during the 100-ms stimulus window (n=100 units). **h-**, Same as in **a-g**, but S1 recordings during SC inactivation (2 sessions from 1 Pitx2-Halo mouse; **j**: spontaneous activity of all S1 units (p = 0.0080, n=70 S1 units); **k,** sensory-evoked responses from whisker responsive S1 units (p=1.00, n=18 units); **l**: baseline-subtracted responses from same units in **k** (p=0.37). **m** Population histogram of OMI for S1 spontaneous activity (n = 70 S1 units) and **n** stimulus-evoked responses (n=70 S1 units). **f, g, m, n**: units with significant modulation are indicated in solid orange colors (p<0.05, two-sided paired t-test). n.s., not significant; n.d., not defined (units with no spikes during analysis window, not included in histogram, but shown in pie charts). **c-d** and **j-l**: **p < 0.01; two-sided Wilcoxon signed rank test, box and whisker plots show median and interquartile ranges; Schematics in **a, h** were adapted from Allen Reference Atlas – Mouse Brain, atlas.brain-map.org. Source data are provided as a Source Data file.

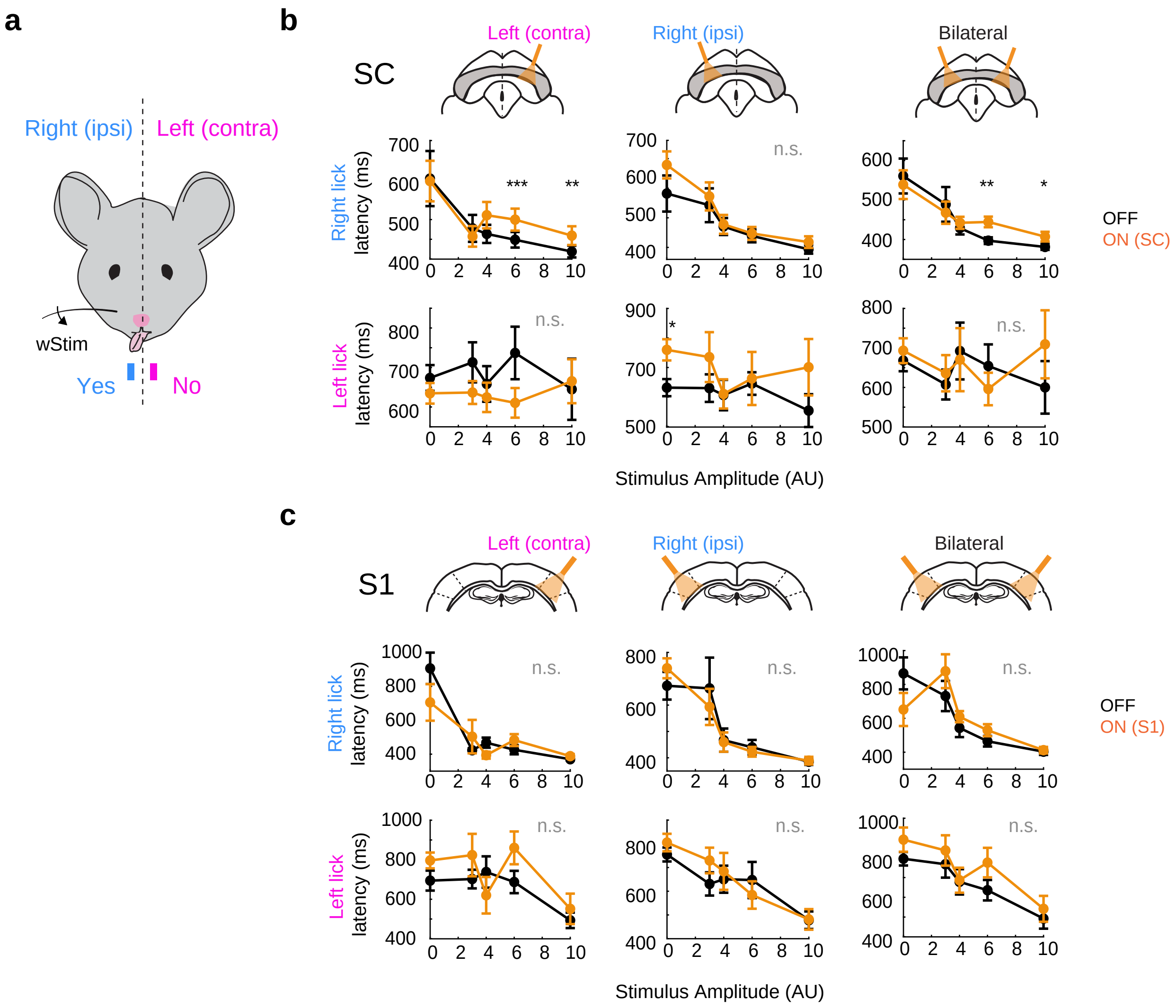

Supplementary Fig. 5 | Effects of unilateral and bilateral S1 or SC inactivation on lick response timing.

a Schematic of the forced-choice, Yes/No tactile detection task. Mice reported stimulus detection by licking right (Yes) and stimulus absence by licking left (No). Ipsi- and contralateral sides are defined here relative the whisker stimulus: animal's right (ipsi, cyan) and left (contra, magenta) sides are indicated. **b** Average lick latencies for the same experiments shown in Fig. 2, SC inactivation in Pitx2-Halo mice in contralateral (left), ipsilateral (right), or bilateral SC. **c** Same as in **b**, but for Emx1-Halo mice with contra, ipsi, or bilateral S1 inactivation from experiments in Fig. 3. Shaded boxes indicate high-confidence responses (ipsi/right licks for amplitude 10, contra/left licks for amp 0); n.s., not significant ($p > 0.05$) for all for OFF vs ON paired values across all amplitudes; * $p < 0.05$, ** $p<0.01$, ***$p<0.001$, Wilcoxon rank-sum test with Bonferroni correction. Schematics in b, c adapted from Allen Reference Atlas – Mouse Brain, atlas.brain-map.org. Source data are provided as a Source Data file.

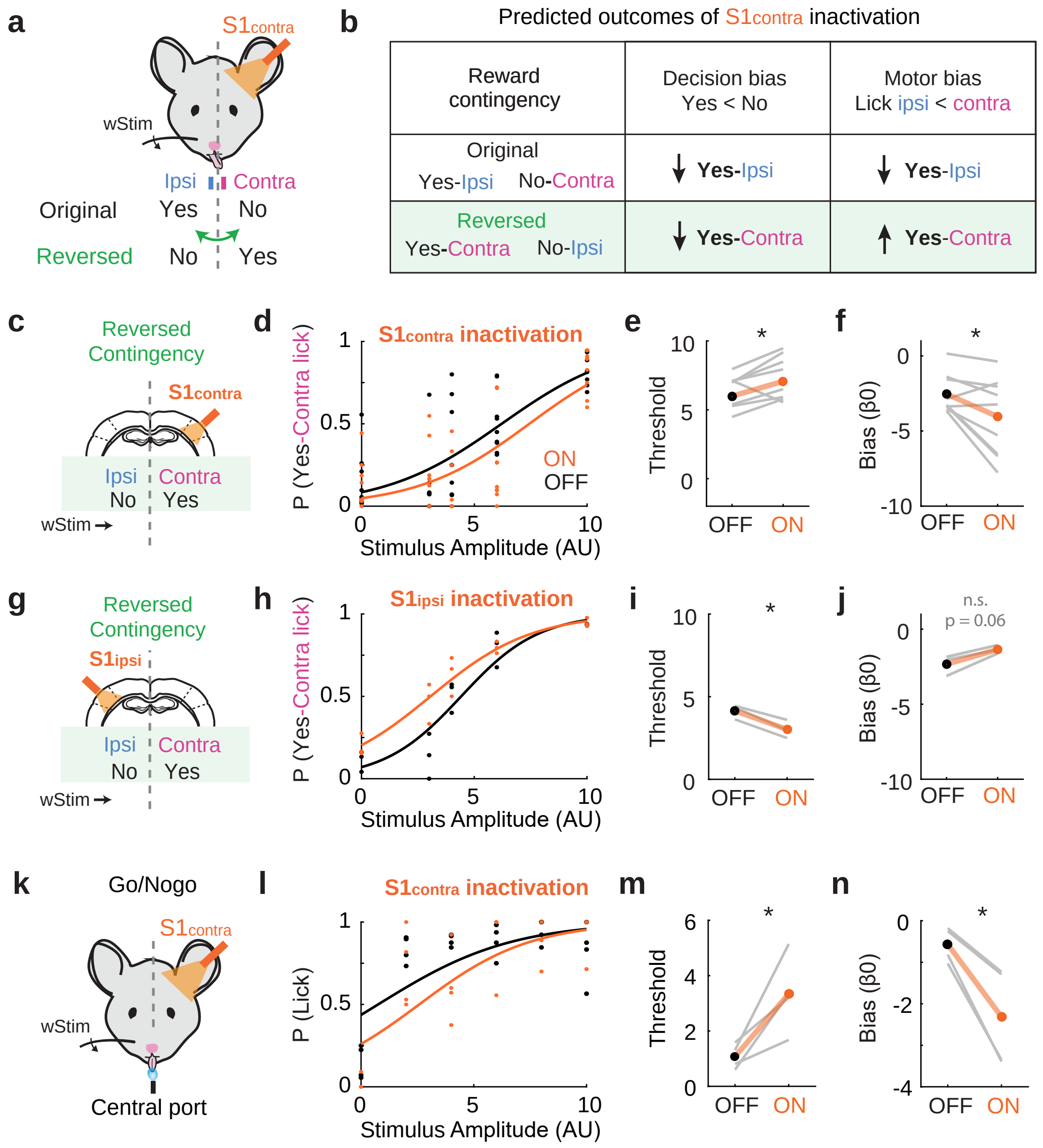

Supplementary Fig. 6 | S1 inactivation induces a decision bias rather than motor bias.

a Original paradigm: S1 inactivation decreased 'Yes' (ipsilateral lick) responses, which could be due to a perceptual bias or motor bias. To distinguish between these, we trained mice on the reversed reward contingency; subjects were trained to lick contralateral to the whisker stimulus for 'Yes' (Yes-contra) or ipsilaterally for a 'No' response-opposite of the original paradigm. **b** Expected outcomes for S1contra inactivation-induced perceptual bias vs. motor biasnder the reversed contingency motor mapping, **c** S1contra inactivation leads to **d**, decreased tendency to respond 'Yes-contra'; **e** increased detection thresholds and **f**, conservative bias (n=3 mice, 9 sessions). **g** S1ipsi inactivation leads to **h**, an increase in 'Yes-contra' responses; **i** decreased threshold and j, liberal bias (n=3 sessions from 1 mouse). **k** S1contra inactivation in animals trained on a Go/No-go version of the task with a single, central port. S1contra inactivation **l**, decreased the probability of lick (P(Lick)); **m**, increased detection threshold and; **n**, conservative decision bias, despite no requirement for directional licking movements (n=5 sessions from 2 mice). These results suggest that bias shifts during S1 inactivation reflect a perceptual, rather than motor bias. Error bars: mean ± s.e.m.; two-sided paired t-tests. Brain schematics in **c, g, k** adapted from Allen Reference Atlas – Mouse Brain, atlas.brain-map.org. Source data are provided as a Source Data file.

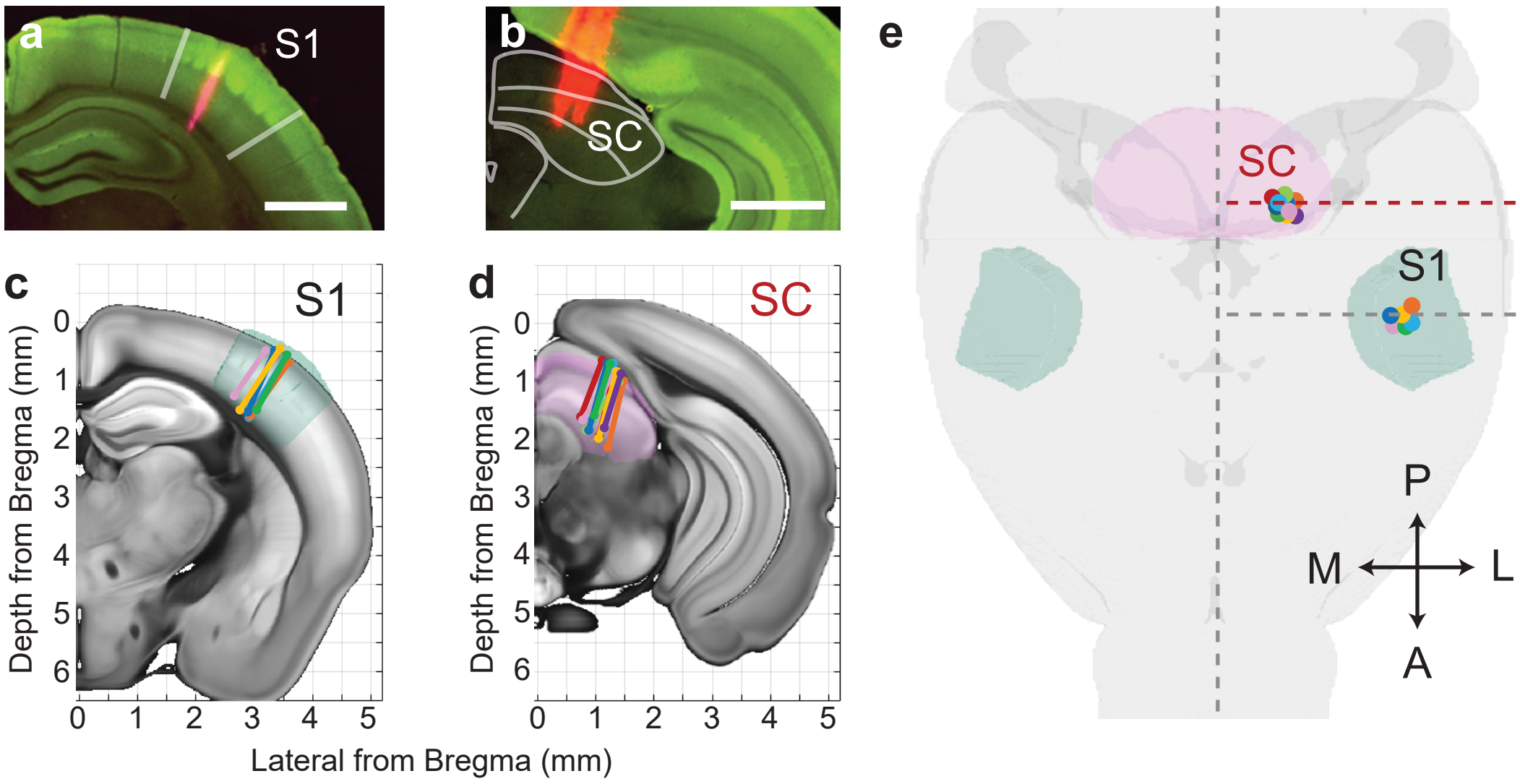

Supplementary Fig. 7 | Recording locations for C2-whisker responsive areas in S1 and SC.

a, Example histology image showing dye-coated probe tracts in S1 and **b**, SC in an Emx1-Halo mouse (n=1 mouse). Confirmed probe locations were overlaid on coronal atlas references from the Neuropixels trajectory explorer software showing targeting relative to Bregma for **c**, S1 (30-degree insertion angle, AP -1.6, ML 3.5, ventral -1.3-mm); and **d**, SC (20-degree insertion angle; AP -3.7, ML 1.7, ventral: -2.5). **e** Top-down view of reconstructed probe locations, overlaid with a mouse brain image from the Neuropixels trajectory explorer software (n= 6 mice). Scale bars in **a**, **b**, 1mm. Brain images in **c**, **d**, adapted from the Allen Common Coordinate Framework using AP-histology software (RRID:SCR_023847); atlas images were adapted from the Allen Reference Atlas – Mouse Brain, atlas.brain-map.org. 3D brain image in **e** was adapted from the Allen Common Coordinate Framework/Allen Reference Atlas – Mouse Brain.

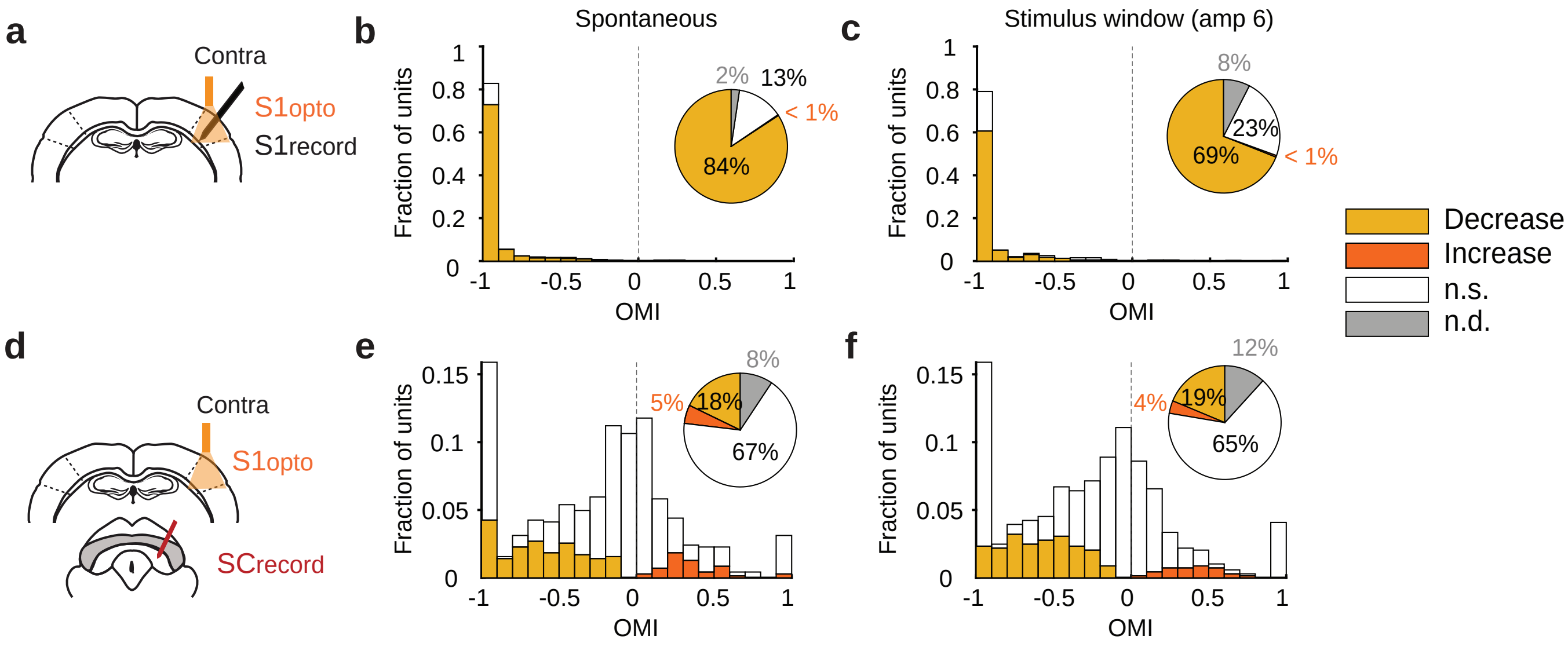

Supplementary Fig. 8 | Optogenetic modulation of S1contra and SCcontra neurons during S1contra inactivation.

a S1contra recordings during optogenetic S1contra inactivation (S1opto, orange). **b** Optogenetic modulation index (OMI = (ON-OFF)/(ON+OFF)) for spontaneous activity and **c** stimulus-evoked activity for near-threshold amp 6 (n=423 S1 units). **d**-**f**, Same as in **a**-**c**, but for SCcontra (n=778 SC units) during S1contra inactivation. Dark orange indicates significantly increased; light orange shows significantly decreased activity with S1 inactivation ($p < 0.05$, two-sided paired t-test). n.s., not significant, n.d., not defined (units with low firing rates that did not spike during the analysis window; gray in pie chart); n = 17 sessions from 6 mice. Brain schematics in **a**, **d** adapted from Allen Reference Atlas – Mouse Brain, atlas.brain-map.org. Source data are provided as a Source Data file.

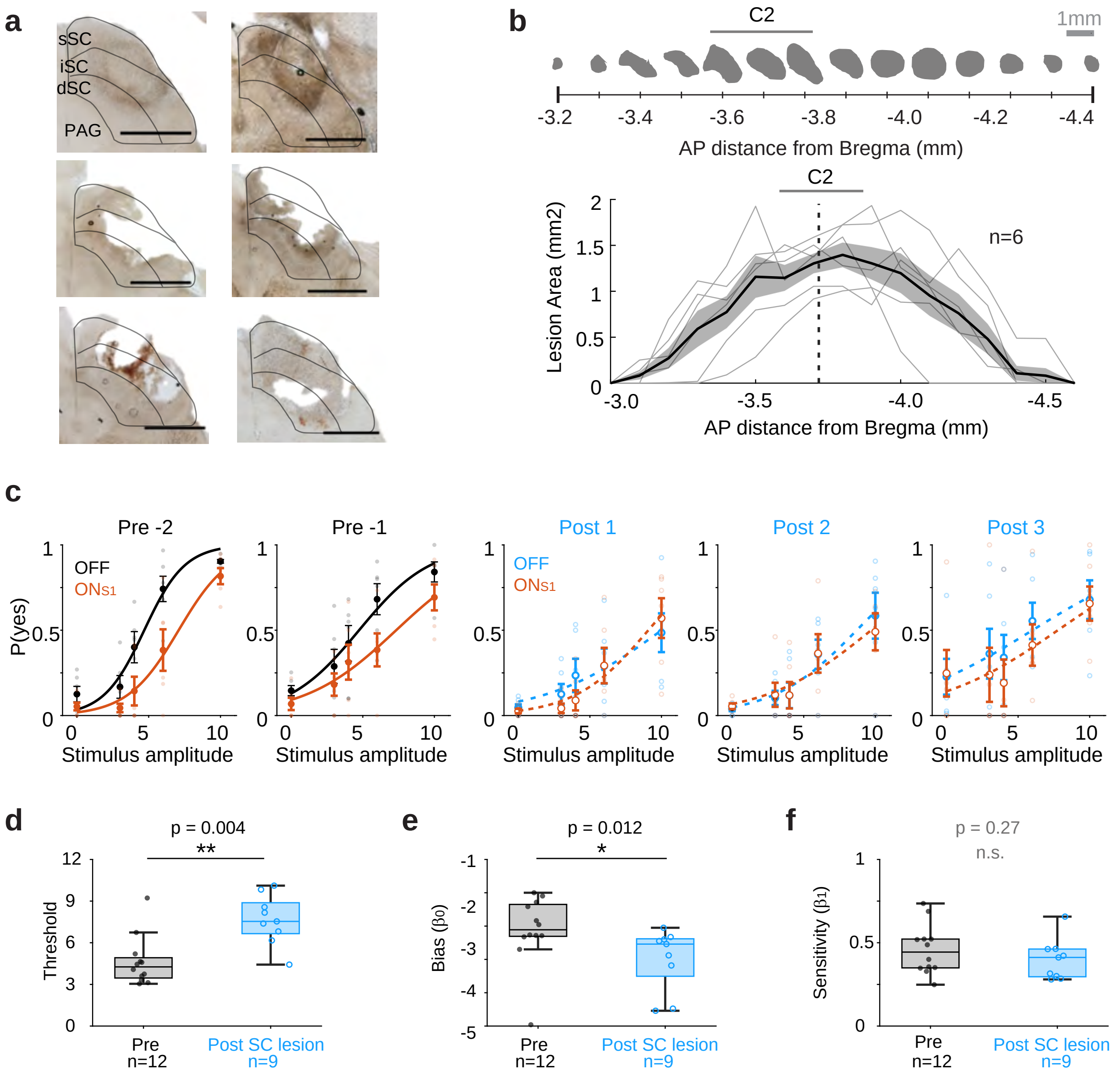

Supplementary Fig. 9 | Superior colliculus lesions and performance across sessions.

a Representative histological sections for SC_{contra} lesions (n=6 mice from Fig. 7). Lesions were targeted to the intermediate SC layers while maximally avoiding deep and lateral SC motor layers. Lesions extending below the SC were excluded from the study. **b** Top: lesion extent across coronal sections in an example mouse. Approximate location of C2-whisker responsive area in SC was identified by dye-labeled probe tracks from recordings (-3.6 to -3.8mm from Bregma). Bottom: quantification of lesion area across each coronal section and approximate AP location from Bregma (n=6 mice, 100-um thick sections per mouse). Shaded error bar: mean and s.e.m. **c** Probability of detection across days (2 days pre- (black) and 3 days post (blue) SC lesion) for laser OFF (black/blue) vs. ON (orange) trials. Dots indicate single animals, error bars indicate mean ± s.e.m. **d-f** Effects of SC lesions on psychometric performance for OFF trials only; **d** Detection threshold (**p = 0.0040); **e,** decision bias (intercept, *p = 0.011); **f,** sensitivity (slope, p = 0.27); two-sided Wilcoxon rank-sum test, n = 12 pre (black, 2 days/mouse), n=9 post (blue, 2 days/mouse); SC lesion sessions before (black) and after SC lesions (blue); circles represent individual sessions. Only sessions with reliable logistic fits (see Methods) were included in these analyses, resulting in variable sample sizes across metrics. Abbreviations: n.s., not significant. SC, superior colliculus; sSC, superficial; iSC, intermediate; and dSC, deep SC layers; PAG, periaqueductal gray. Scale bars: 1mm.

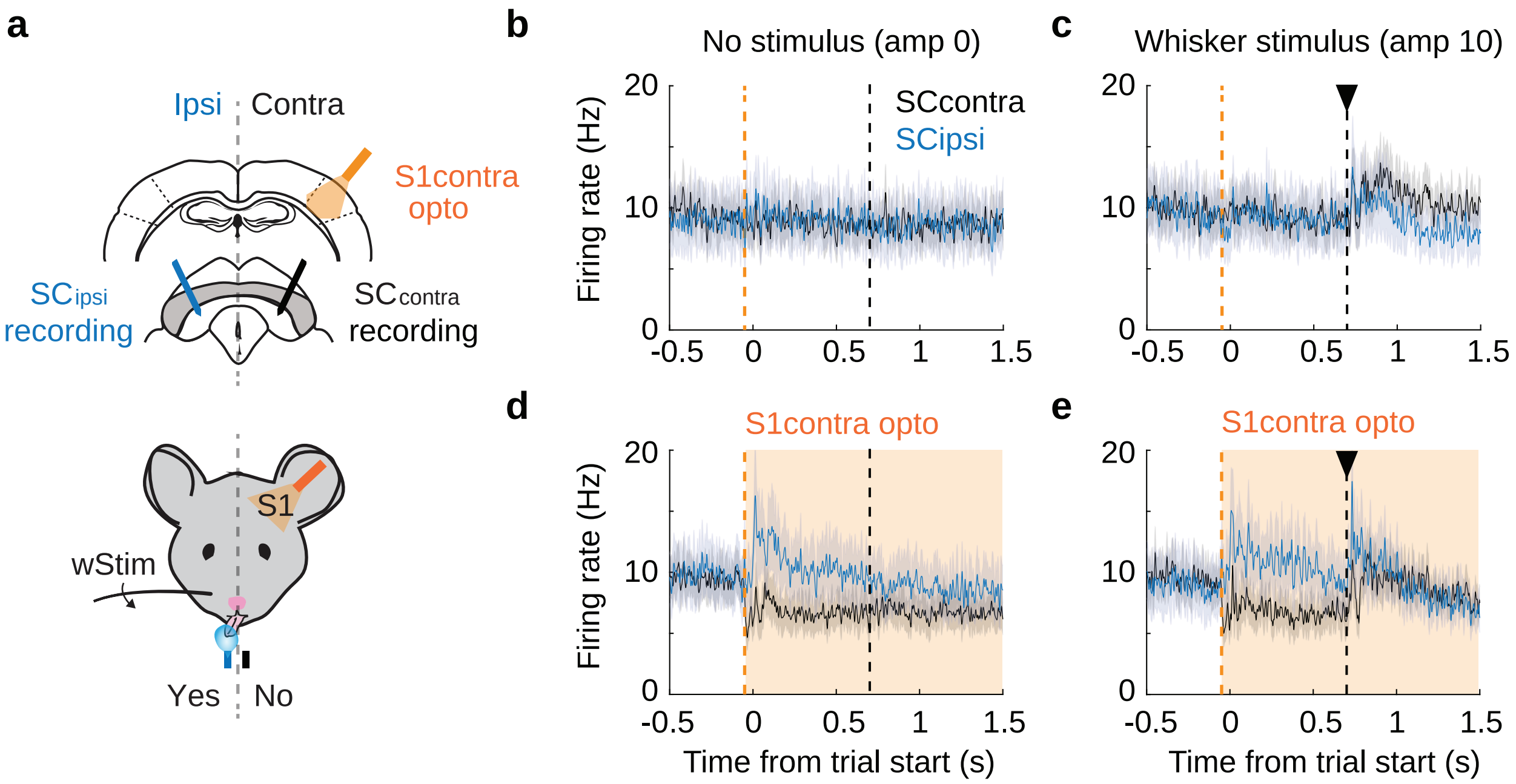

Supplementary Fig. 10 | Unilateral S1 inactivation induces an interhemispheric imbalance in SC activity.

a Schematic of experiment. Ipsilateral (ipsi) and contralateral (contra) sides are defined relative to the whisker stimulus. S1 contralateral to the whisker stimulus ($S1_{contra}$) was optogenetically inactivated during simultaneous bilateral recordings in ipsilateral (SC_{ipsi}) and contralateral SC (SC_{contra}) during the Yes/No detection task. **b**, **c**, Average firing rates during laser-OFF trials for **b**, no-stimulus trials and **c**, high-amplitude whisker stimulus trials. **d**, **e**, Average firing rates during S1contra-inactivation trials ($S1_{contra}$ opto) for **d**, no-stimulus trials and **e**, high-amplitude stimulus trials. During S1 inactivation, activity decreased in SC_{contra} and increased in SC_{ipsi}, consistent with an interhemispheric imbalance in SC activity. Orange shading indicates period of laser illumination; orange dashed lines indicate laser onset (50 ms before trial start); black triangles and dashed lines indicate stimulus onset. SC_{contra} recordings shown in black; SC_{ipsi} in blue. Solid lines and shaded regions indicate mean and s.e.m. across units. n=1 session, SC_{contra}: n = 48 single units, SC_{ipsi}: n = 33 single units. Brain schematics adapted from Allen Reference Atlas – Mouse Brain, atlas.brain-map.org.
