# Supplementary material for: Somatosensory cortex shapes perceptual decision bias via the superior colliculus
Source: Nat Commun. 2026 Oct 10;17:11515. doi: 10.1038/s41467-026-78331-0 (PMC13745596; doi:10.1038/s41467-026-78331-0)
Supplement: Supplementary file 2 [file 41467_2026_78331_MOESM2_ESM.pdf]

Description of Additional Supplementary Files

File Name: Supplementary Data 1

Description: Animal information for subjects across experiments.

The dataset lists all animals included in the study referenced for each figure panel. Animal information, including name, sex, genotype, and age at the end of the experiment are listed.

File Name: Supplementary Data 2

Description: Reference information for electrophysiological units analyzed in Figs. 4-6.

The reference table lists the S1 and SC units included for all analyses. Units from each session and animal are indicated. A session-level summary indicates the animal and number of S1 and SC units recorded for each session.
