# Supplementary material for: Somatosensory cortex shapes perceptual decision bias via the superior colliculus
Source: Nat Commun. 2026 Oct 10;17:11515. doi: 10.1038/s41467-026-78331-0 (PMC13745596; doi:10.1038/s41467-026-78331-0)
Supplement: Supplementary file 3 [file 41467_2026_78331_MOESM5_ESM.pdf]

nature portfolio

Corresponding author(s): Y. Kate Hong

Last updated by author(s): 8/28/2026

Reporting Summary

Nature Portfolio wishes to improve the reproducibility of the work that we publish. This form provides structure for consistency and transparency in reporting. For further information on Nature Portfolio policies, see our Editorial Policies and the Editorial Policy Checklist.

Statistics

For all statistical analyses, confirm that the following items are present in the figure legend, table legend, main text, or Methods section.

n/a	Confirmed	
☐	☒	The exact sample size (*n*) for each experimental group/condition, given as a discrete number and unit of measurement
☐	☒	A statement on whether measurements were taken from distinct samples or whether the same sample was measured repeatedly
☐	☒	The statistical test(s) used AND whether they are one- or two-sided<br>*Only common tests should be described solely by name; describe more complex techniques in the Methods section.*
☒	☐	A description of all covariates tested
☐	☒	A description of any assumptions or corrections, such as tests of normality and adjustment for multiple comparisons
☐	☒	A full description of the statistical parameters including central tendency (e.g. means) or other basic estimates (e.g. regression coefficient) AND variation (e.g. standard deviation) or associated estimates of uncertainty (e.g. confidence intervals)
☐	☒	For null hypothesis testing, the test statistic (e.g. *F*, *t*, *r*) with confidence intervals, effect sizes, degrees of freedom and *P* value noted<br>*Give P values as exact values whenever suitable.*
☒	☐	For Bayesian analysis, information on the choice of priors and Markov chain Monte Carlo settings
☐	☒	For hierarchical and complex designs, identification of the appropriate level for tests and full reporting of outcomes
☒	☐	Estimates of effect sizes (e.g. Cohen's *d*, Pearson's *r*), indicating how they were calculated

Our web collection on statistics for biologists contains articles on many of the points above.

Software and code

Policy information about availability of computer code

Data collection	Electrophysiology data was collected using open-source software (OpenEphys v5). Behavioral data was collected using serial output from custom written scripts in open-source Arduino IDE software. Image analysis was performed using open-source software ImageJ v1.52n. High-speed video acquisition was done using freely available Basler software, and whisker tracking was performed using open-source software (DeepLabCut v2.3.8 , https://github.com/DeepLabCut).
Data analysis	Spike sorting was done using open source software Kilosort 2.5 and 3.0 and Phy2. All other analyses were performed using custom-written scripts in MATLAB. Custom MATLAB scripts are publicly available at Zenodo (Nam, A., Shin, J., Tenney, M., Zhang, B. (Lily) ., & Hong, K. (2026). Somatosensory cortex shapes perceptual decisions via the superior colliculus (analysis code) [Computer software]. Zenodo. https://doi.org/10.5281/zenodo.22117824)

For manuscripts utilizing custom algorithms or software that are central to the research but not yet described in published literature, software must be made available to editors and reviewers. We strongly encourage code deposition in a community repository (e.g. GitHub). See the Nature Portfolio guidelines for submitting code & software for further information.

Data

Policy information about availability of data

All manuscripts must include a data availability statement. This statement should provide the following information, where applicable:

- Accession codes, unique identifiers, or web links for publicly available datasets
- A description of any restrictions on data availability
- For clinical datasets or third party data, please ensure that the statement adheres to our policy

All data used to generate results, including behavioral and electrophysiological recordings, are publicly available at Zenodo (Nam, A., Shin, J., Tenney, M., Zhang, B. (Lily) ., & Hong, K. (2026). Somatosensory cortex shapes perceptual decisions via the superior colliculus (source data) [Dataset]. Zenodo. https://doi.org/10.5281/zenodo.22119217)

Research involving human participants, their data, or biological material

Policy information about studies with human participants or human data. See also policy information about sex, gender (identity/presentation), and sexual orientation and race, ethnicity and racism.

Reporting on sex and gender	N/A
Reporting on race, ethnicity, or other socially relevant groupings	N/A
Population characteristics	N/A
Recruitment	N/A
Ethics oversight	N/A

Note that full information on the approval of the study protocol must also be provided in the manuscript.

Field-specific reporting

Please select the one below that is the best fit for your research. If you are not sure, read the appropriate sections before making your selection.

[x] Life sciences [ ] Behavioural & social sciences [ ] Ecological, evolutionary & environmental sciences

For a reference copy of the document with all sections, see nature.com/documents/nr-reporting-summary-flat.pdf

Life sciences study design

All studies must disclose on these points even when the disclosure is negative.

Sample size	No statistical method was used to predetermine sample size. Sample sizes were chosen based on prior studies using a similar whisker-detection paradigm and optogenetic perturbation done in previous work (Hong et al., 2018). The sample sizes were sufficient to provide repeated measurements across animals and recording sessions. Exact numbers of animals, sessions, and units are reported in the figure legends, Methods, and Source Data.
Data exclusions	Exclusion criteria was based on animals' baseline behavioral performance and electrophysiology recording quality. For electrophysiology experiments, if the animals responded to fewer than 100 trials due to early satiation, sessions were not included. For SC lesion experiments: animals were excluded from analysis using histological criteria outlined in the methods (target size and location); Electrophysiological recording sessions were excluded if fewer than 10 single units were isolated, or if no stimulus-responsive units were found (indication of missed target).
Replication	All attempts at replication were successful. Behavioral experiments were repeated across different cohorts for S1 contralateral inactivation, and SC perturbation experiments were performed using multiple methods (optogenetic silencing and lesions).
Randomization	Trial types (stimulus amplitudes and optogenetic inactivation) were randomly interleaved during behavioral sessions. Male and female mice were randomly assigned across cohorts.
Blinding	Scoring lesions for inclusion criteria (location and size) were done blind to animal identity and experimental outcomes.

Reporting for specific materials, systems and methods

We require information from authors about some types of materials, experimental systems and methods used in many studies. Here, indicate whether each material, system or method listed is relevant to your study. If you are not sure if a list item applies to your research, read the appropriate section before selecting a response.

Materials & experimental systems

n/a	Involved in the study
☒	☐ Antibodies
☒	☐ Eukaryotic cell lines
☒	☐ Palaeontology and archaeology
☐	☒ Animals and other organisms
☒	☐ Clinical data
☒	☐ Dual use research of concern
☒	☐ Plants

Methods

n/a	Involved in the study
☒	☐ ChIP-seq
☒	☐ Flow cytometry
☒	☐ MRI-based neuroimaging

Animals and other research organisms

Policy information about studies involving animals; ARRIVE guidelines recommended for reporting animal research, and Sex and Gender in Research

Laboratory animals	Adult mice (P181 +/- 47 days, mean and SD) were used across all genotypes. All mice were maintained on a C57/BL6J background. Emx1-IRES-Cre or Pitx2-Cre mice were crossed with Ai39 (Rosa-flex-eNpHR3.0) or ROSA-H2B-GFP mice.
Wild animals	N/A
Reporting on sex	Both male and females were used in roughly equal numbers (58% male)
Field-collected samples	N/A
Ethics oversight	All animal procedures were performed in accordance with protocols approved by the Institutional Animal Care and Use Committee at Carnegie Mellon University

Note that full information on the approval of the study protocol must also be provided in the manuscript.

Plants

Seed stocks	N/A
Novel plant genotypes	N/A
Authentication	N/A
