# Supplementary material for: Somatosensory cortex shapes perceptual decision bias via the superior colliculus
Source: Nat Commun. 2026 Oct 10;17:11515. doi: 10.1038/s41467-026-78331-0 (PMC13745596; doi:10.1038/s41467-026-78331-0)
Supplement: Supplementary file 4 [file 41467_2026_78331_MOESM6_ESM.pdf]

nature portfolio

Peer Review File

Somatosensory cortex shapes perceptual decision bias via the superior colliculus

Corresponding Author: Professor Youn-Young Hong

This file contains all reviewer reports in order by version, followed by all author rebuttals in order by version.

Version 0:

Reviewer comments:

Reviewer #1

(Remarks to the Author)
This paper reports interactions between primary somatosensory cortex (S1) and superior colliculus (SC) that together guide perceptual decision-making in mice. In a whisker detection task, unilateral S1 inhibition increases detection threshold without strongly affecting sensitivity, whereas inhibition of the ipsilateral hemisphere unexpectedly decreases threshold, consistent with shifts in detection bias. SC inhibition also bidirectionally shifts detection bias, consistent with interhemispheric competition that biases motor choice. Neural recordings reveal stimulus and choice signals in both regions, with S1 showing stronger sensory encoding and SC showing stronger choice representations. Simultaneous recordings during S1 inactivation show that SC activity tracks the pattern of behavioral choice, and SC lesions demonstrate that S1's effect on bias depends on SC.

Overall, this is an interesting and carefully executed study that dissects cortex–SC interactions and reveals a significant role for SC in tactile perceptual decisions. The interhemispheric interaction in cortex is interesting, and the finding that they depend on SC is significant. The conclusion that primary sensory areas can shape perceptual decisions by modulating SC is timely and of broad interest to the Nature Communications audience. The study represents a large amount of work that is carefully done. I have a few suggestions that I hope will help the authors strengthen the clarity of their mechanistic interpretation and better support their core claims.

Major:
1) The central conclusion is that "S1 shapes behavioral bias via its downstream modulation of SC." The manuscript clearly demonstrates that S1 affects SC activity and that SC is necessary for S1-induced behavioral bias. However, two mechanistic links are not yet clear and would benefit from further articulation: (1) how S1 influences choice-related activity in SC, and (2) how changes in SC activity during S1 silencing give rise to the observed decision bias. For example, how does S1 silencing affect SC neurons encoding choice? Does this change SC activity in a way that alters interhemispheric competition? To be clear, I'm not asking for new experiments. Rather, additional clarification using existing data or analyses would help strengthen the mechanistic insight.

2) Related to the question above, analysis of the existing SC recordings during S1 inhibition might offer additional insights. The key supporting analysis in Fig. 6 is based on trial-averages, which makes it difficult to see how shifts in SC activity translates into behavioral bias on individual trials. At the same time, current decoding analysis uses separate decoders for control and perturbation trials, precluding examination of activity change across these conditions and how it might manifest in altered choice. It would be interesting to know how a choice decoder trained on control trials generalizes to perturbation trials.

3) The contingency reversal experiment in Extended Data Fig 4 is particularly interesting, and clarifying the following would help strengthen mechanistic interpretation. Across both sensorimotor contingencies, S1 inhibition increases detection threshold. In the reversed contingency, this produces an increased contralateral licking bias. However, this would appear opposite to the direction expected from unilateral SC inhibition, which typically biases choices ipsilaterally. If these effects indeed diverge, it may suggest that S1 influences SC in a more complex manner than a simple unidirectional modulation. Without adding new experiments, could the authors expand on their conceptual model to clarify how S1–SC interactions give rise to bias across contingencies?

Minor:
1) Do SC neurons exhibit tunings for both ipsilateral and contralateral choice? Is there a bias in their proportion that is congruent with the lateralized perturbation effects shown in Fig 2?

2) Figs. 2o and 3n: Is there an increase in reaction time during bilateral S1 or SC silencing?

3) Fig. 6f: The increase in SC decoding accuracy after S1 silencing is interesting. Can the authors provide further insights into what is driving this increase? Is this increase driven by hit trials, correct reject trials, or both?

4) Extended Data Fig. 6: Please indicate the neurons that are significantly modulated. Many SC neurons show disinhibition (OMI > 0). Are these changes statistically significant?

5) Line 137: "…express inhibitory halorhodopsin in ~50% of excitatory neurons in the intermediate SC…" Is the 50% estimate based on cell counting in the current dataset or from a previous reference?

6) Fig 7: Do the dots represent individual mice?

7) Line 356: "…S1 provides flexible control over decision-bias". Perhaps consider removing the word "flexible" since this was not explicitly tested.

8) Line 368-371: The sentence stating that S1's "major influence" is on decision bias rather than sensory encoding could be clarified. The data clearly shows that S1 affects decision bias via SC, but this does not preclude additional sensory role of S1, since bilateral S1 silencing did reduce sensitivity.

9) In the method section (line 689,690), why are different number of units used for stimulus and choice decoding?

10) A few typos:
Line 83: the figure reference should probably be Fig. 1a-b.
Line 118, "overall changes in sensitivity did not differ". Perhaps the authors mean that overall sensitivity did not differ, or changes in sensitivity were small or inconsistent.
Line 146: typo, "(Fig. d-g)" should probably be "(Fig. 2d-g)".
Line 252: figure references should probably be Fig. 4d or 4e.
Line 288 is missing a figure number.
Line 507: typo, repetition of "turned on."
Line 538, the reference to Extended Data Fig. 6 should probably be removed.
Tick labels are misaligned in Fig 3d-e, i-j, n-o
Figure 2i–m legend, the description may be missing the phrase "SC ips" before "inactivation."

Nuo Li

Reviewer #2

(Remarks to the Author)
This fascinating and rigorous study by Nam et al. investigates the coordination between sensory cortex and superior colliculus in the context of stimulus detection. Their conclusions are founded on psychometric evaluations of a Yes/No whisker detection task, paired with neuronal recordings and perturbations. The manuscript makes the following novel conclusions which contrast with traditional views of sensory cortex contributions to sensory detection: 1) sensory cortex causally contributes mostly to bias rather than sensitivity and 2) this function is dependent on the superior colliculus. The data, controls, and analyses appear to be of high quality. My most substantial concerns are with some of the interpretations, which can be addressed with additional analyses and discussion.

1. I'm stuck on other possible interpretations of a rightward shift of the psychometric curve. While I agree that 'bias' is the most straightforward interpretation, it may not be the only one. Consider two parallel sensory pathways with similar neurometric sensitivities but different thresholds. The low threshold system would normally be driving detection decisions as it would be activated by lower stimulus intensities. However, if removed, then the high threshold system would take over. This would appear as a shift in bias. Furthermore, this may map on to the lemniscal (low threshold, requiring S1) and paralemniscal (high threshold, possibly bypassing S1) whisker pathways. Can the authors rule out this organization?

2. SC as the 'bottleneck'. The results displayed in Fig 2n-o are extremely interesting, and suggest active competition between SC hemispheres in setting the bias. However, the finding that bilateral suppression causes no change in the psychometric function would seem to undermine the statement in the abstract of "SC as the critical bottleneck in perceptual decisions" (as well as similar statements in the Results and Discussion). From my understanding of a bottleneck, I would expect suppression to block perceptual decisions (reduce response rates). Please clarify.

3. The 'unmasking' of an S1 contribution to sensitivity is very interesting (Fig 3m-q). Do you suspect that it is the same population of S1 neurons that contribute to both bias and sensitivity?

4. An important missing reference is Luo and Maunsell 2015. By manipulating reward, the authors were able to behaviorally dissociate changes in bias vs sensitivity, and showed that changes in V4 visual cortex of monkeys track changes in

sensitivity only. The conclusions of the Luo and Maunsell work conflict with the conclusions of the current study. Please address in the Discussion.

5. The Introduction provides a very superficial overview of the relevant SC, sensory cortex, and barrel cortex literature related to sensory detection as well as computational and neural studies regarding the establishment of bias. More in-depth treatments of these topics would help contextualize the current work. The final two paragraphs (and especially the final paragraph) related to the current study can be significantly shortened if needed. [On further reading, some of the relevant background information is contained in the Results, which can be moved to the Introduction.]

6. It would be very interesting to know whether inhibition of S1ipsi in the Go/Nogo context also causes a leftward shift in the psychometric curve (Extended Data Fig. 4k-n). If so, it would generalize the bidirectional control beyond the forced choice task design. If the data is not already in-hand, this could be a Discussion point.

7. The SC lesion data (Fig 7b) appears markedly different from the SC opto-suppression data (Fig 2d-h). It would be helpful to include similar quantifications for the SC lesion data. If SC lesions are indeed reducing sensitivity, how would this impact the conclusions of the study?

8. I agree that the current study provides a compelling hypothesis regarding the paradox of acute vs chronic causality. However, to truly 'resolve' the paradox, one would need to actually demonstrate the proposed mechanism of recovery.

9. Entirely up to the authors: it may be useful to include the finding of S1 modulating bias in the title.

Reviewer #3

(Remarks to the Author)
Nam et al. provide compelling evidence through an impressive array of rigorous experiments to show that SC activity controls the decision-making threshold. Moreover, they show that S1's role in decision-making is mediated through SC. This work provides a critical and long-overdue understanding of the cortical contribution to behaviors not involving the discrimination of fine-scale stimulus features. Therefore, this work will have a long-lasting impact on the field by improving the way people design experiments and interpret results. Below are some relatively minor comments that may help improve the manuscript. I have no major concerns.

1. In this manuscript, the authors focus on response probability as the decision outcome. Is there a shift in response latency that is consistent with the observed shift in decision bias? For instance, when you silence ipsilateral SC, does response latency shorten for the lower amplitude stimuli?

2. They show that "SC inactivation" does not alter S1 activity. However, their data show that SC inactivation is quite modest. Might this explain their result? It would be useful to show the SC deactivation results in the format of extended data fig. 6.

3. Did you trying restricting your analysis to L5 neurons in S1 during SC silencing?

4. On line 191, they authors state that ascending whisker inputs are entirely lateralized. While technically true, ipsilateral responses do occur in S1 and are quite prevalent for the A-row barrels. However, these ipsilateral responses originate from the contralateral S1, which is not really considered the ascending pathway. So, this is a minor detail.

5. on Line 689 they state the use of 423 neurons for stimulus decoding and 282 neurons for choice decoding. It is not clear why there are a different number of neurons used.

6. The authors use the Pitx2-Cre line to silence SC neurons. Might there be non-Pitx2 neurons that are more specialized for sensory coding and could affect stimulus sensitivity?

7. The authors show that bilateral S1 silencing reduces sensitivity. What S1-->target circuit could be contributing to the reduction in response probability at near-threshold stimulus intensities?

8. Seems worth citing James Sprague: https://www.science.org/doi/10.1126/science.153.3743.1544

Version 1:

Reviewer comments:

Reviewer #1

(Remarks to the Author)
The authors have carefully and thoroughly addressed my comments with new analyses, additional experiments, and substantial revisions to the text. The revised manuscript is significantly improved, and the authors have strengthened the mechanistic interpretation of how S1 influences perceptual decisions through SC. This paper provides compelling evidence that primary sensory areas can shape perceptual decisions by modulating SC, and the findings are likely to make an important contribution to our understanding of how brain-wide circuits support perceptual decision-making. I am satisfied

with the revision and strongly support publication.

Reviewer #2

(Remarks to the Author)
Thank you for the comprehensive responses, my concerns have been addressed.

Reviewer #3

(Remarks to the Author)
The authors have addressed all of my concerns. Excellent manuscript.

Open Access This Peer Review File is licensed under a Creative Commons Attribution 4.0 International License, which permits use, sharing, adaptation, distribution and reproduction in any medium or format, as long as you give appropriate credit to the original author(s) and the source, provide a link to the Creative Commons license, and indicate if changes were made.
In cases where reviewers are anonymous, credit should be given to 'Anonymous Referee' and the source.
The images or other third party material in this Peer Review File are included in the article's Creative Commons license, unless indicated otherwise in a credit line to the material. If material is not included in the article's Creative Commons license and your intended use is not permitted by statutory regulation or exceeds the permitted use, you will need to obtain permission directly from the copyright holder.
To view a copy of this license, visit https://creativecommons.org/licenses/by/4.0/

SUMMARY
We thank the reviewers for their strong enthusiasm and helpful suggestions for the manuscript.

We were encouraged that the reviewers recognized the significance and broad interest of the study. Reviewer 1 (R1) noted that our "conclusion that primary sensory areas can shape perceptual decisions by modulating SC is timely and of broad interest to the Nature Communications audience." Reviewer 2 (R2) described the work as a "fascinating and rigorous study," and Reviewer 3 (R3) comments that the work provides "compelling evidence through an impressive array of rigorous experiments" and "will have a long-lasting impact on the field."

Based on reviewers' insightful comments, we have extensively revised the manuscript by adding new analyses, rewriting introduction for a more in-depth literature review, and added new discussion points in the Discussion. We address each reviewers' points below, which we believe has significantly strengthened the manuscript.

[R1] Reviewer #1 (Remarks to the Author):

This paper reports interactions between primary somatosensory cortex (S1) and superior colliculus (SC) that together guide perceptual decision-making in mice. In a whisker detection task, unilateral S1 inhibition increases detection threshold without strongly affecting sensitivity, whereas inhibition of the ipsilateral hemisphere unexpectedly decreases threshold, consistent with shifts in detection bias. SC inhibition also bidirectionally shifts detection bias, consistent with interhemispheric competition that biases motor choice. Neural recordings reveal stimulus and choice signals in both regions, with S1 showing stronger sensory encoding and SC showing stronger choice representations. Simultaneous recordings during S1 inactivation show that SC activity tracks the pattern of behavioral choice, and SC lesions demonstrate that S1's effect on bias depends on SC.

Overall, this is an interesting and carefully executed study that dissects cortex–SC interactions and reveals a significant role for SC in tactile perceptual decisions. The interhemispheric interaction in cortex is interesting, and the finding that they depend on SC is significant. The conclusion that primary sensory areas can shape perceptual decisions by modulating SC is timely and of broad interest to the Nature Communications audience. The study represents a large amount of work that is carefully done. I have a few suggestions that I hope will help the authors strengthen the clarity of their mechanistic interpretation and better support their core claims.

We thank R1 for recognizing the broad impact and rigor of our work. We address each of the comments below.

Major:
Major 1) The central conclusion is that "S1 shapes behavioral bias via its downstream modulation of SC." The manuscript clearly demonstrates that S1 affects SC activity and that SC is necessary for S1-induced behavioral bias. However, two mechanistic links are not yet clear and would benefit from further articulation: (1) how S1 influences choice-related activity in SC, and (2) how changes in SC activity during S1 silencing give rise to the observed decision bias. For example, how does S1 silencing affect SC neurons encoding choice? Does this change SC activity in a way that alters interhemispheric competition? To be clear, I'm not asking for new experiments. Rather, additional clarification using existing data or analyses would help strengthen the mechanistic insight.

We thank the reviewer for the probing questions, and for encouraging us to expand on how S1-dependent modulation of SC could give rise to behavioral bias. Our main conclusion is that S1 inactivation impairs tactile detection not simply due to decreasing sensory information in S1, but by altering SC-dependent processes that bias behavioral choice. While our goal was not to define the mechanism by which SC induces bias formation, we provide our hypothesis based on the current findings and grounded in prior literature, which is outlined below. The hypothesized mechanism is now incorporated into the revised Discussion**:**

We speculate that S1 modulates the gain and baseline excitability of SC neurons such that sensory-driven activity is more or less likely to reach the decision threshold required to trigger the mapped behavioral response. Several key results are consistent with this model:

(i) $S1_{contra}$ inactivation decreases stimulus-evoked responses in SC (Fig. 5 k, l), which may decrease the probability that tactile input reaches the threshold needed for a 'yes' response. However, we note that decreased sensory drive alone doesn't explain the bias across all amplitudes, also observed for no-stimulus trials. The behavioral change was better explained by a shift in bias, rather than decreased sensitivity (Fig 1j, k).

(ii) $S1_{contra}$ inactivation decreased baseline activity in SC_{contra}. Unilateral changes in SC activity can lead to an interhemispheric imbalance across SC hemispheres (Sprague 1986, Essig et al., 2021). To further support this idea, we have added data from a bilateral SC recording session (**newly added Extended Data Fig. 10**), which suggest that acute $S1_{contra}$ inactivation not only decreases SC_{contra} baseline activity, but transiently increases SC_{ipsi} activity, further contributing to the interhemispheric imbalance. Consistent with models of interhemispheric competition (Herman et al., 2018), this may induce an offset that changes how much sensory evidence is needed to reach the threshold for a 'yes' response. This model is consistent with the overall bias shifts we observe, including changes in false alarm rates.

(iii) Our choice decoding results (**Fig. 6f**) reflect population activity during the early stimulus window (100 ms from whisker stimulus onset), before licking or reward. We therefore do not interpret this choice-related activity as a simple motor representation of lick direction, but that SC population activity during the stimulus period contains information that is most predictive of subsequent choice.

(iv) The reversed-contingency experiments (now Extended Data Fig. 5) strongly argue against a simple motor bias effect, since $S1_{contra}$ inactivation effects followed the perceptual choice (yes/no) independent of directional motor output (lick right/left). Thus, the dominant effect of S1 inactivation is a shift in perceptual bias, but we cannot rule out whether there is a weaker effect on motor bias that was masked by the larger perceptual bias.

From our current data, we cannot determine whether inactivation also alters the premotor or motor output neurons that predict lick direction located in lateral and deep SC. Notably, S1 axons project to not only to the stimulus-responsive neurons in the intermediate layers, but also intermediate and deep SC layers where premotor and output neurons reside (**newly added Extended Data Fig. 3**). One possibility is that S1 inactivation additionally affects premotor/output neurons. Alternatively, sensory-responsive SC neurons may indirectly bias these premotor populations through interlaminar SC or downstream brain circuits. Future studies are needed to test this possibility.

Thus, while we do not purport to have resolved how SC mediates perceptual decisions, we believe this

model is consistent with existing literature and interpretation the current data. We have extensively revised the Discussion to explicitly include this working model on how S1 alters behavior via changes in SC (lines 421-25, 445-456): S1 inactivation does not simply remove sensory information from S1, but alters the excitability and interhemispheric balance of SC circuits, biasing how sensory evidence is transformed into behavioral choice.

Major 2) Related to the question above, analysis of the existing SC recordings during S1 inhibition might offer additional insights. The key supporting analysis in Fig. 6 is based on trial-averages, which makes it difficult to see how shifts in SC activity translates into behavioral bias on individual trials. At the same time, current decoding analysis uses separate decoders for control and perturbation trials, precluding examination of activity change across these conditions and how it might manifest in altered choice. It would be interesting to know how a choice decoder trained on control trials generalizes to perturbation trials.

This is an excellent suggestion. Our original decoding analysis was performed separately for OFF vs. ON conditions, which does not address whether the population code is generalizable across conditions. We agree that determining whether the population code trained in the normal, intact (OFF) condition remains valid during S1 silencing (ON) would provide a more meaningful analysis.

As suggested, we now perform cross-condition generalization decoding analysis. We trained linear SVM decoders on OFF trials and tested either on held-out OFF trials or ON trials, using the same stimulus window as the original analysis. Across decoders, we balanced the number of eligible units across trial condition (OFF vs. ON) as well as lick direction ('yes' = right; 'no' = left) to control for potential motor effects. In addition, the number of units are now matched between stimulus and choice decoding analyses (in response to R1 comment #9 and R3 comment #5 below).

The updated cross-generalization analyses suggest that SC contains a stable decision axis that is robust to cortical perturbation, supporting our original conclusions. Specifically, for stimulus decoding, OFF-trained decoders poorly generalized to ON trials in both S1 and SC (**Fig. 6e**, updated), suggesting that S1 inactivation altered the stimulus-evoked population representation in both areas. In contrast, for choice decoding, the decoder failed to generalize in S1, but remained robust in SC (**Fig. 6f,** updated**)**, suggesting that while S1 inactivation disrupts the stability of S1 choice representations, SC maintains a choice-predictive code that remains stable across OFF and ON conditions. Importantly, the reanalysis supports our original interpretation that $S1_{contra}$ inactivation shifts decision bias through downstream effects on SC, rather than simply decreasing sensory information from S1.
We have updated **Fig. 6e-f** accordingly, and edited the Results (lines 226-344) and Methods (lines 763-768) to reflect the new analysis.

Major 3) The contingency reversal experiment in Extended Data Fig 4 is particularly interesting, and clarifying the following would help strengthen mechanistic interpretation. Across both sensorimotor contingencies, S1 inhibition increases detection threshold. In the reversed contingency, this produces an increased contralateral licking bias. However, this would appear opposite to the direction expected from unilateral SC inhibition, which typically biases choices ipsilaterally. If these effects indeed diverge, it may suggest that S1 influences SC in a more complex manner than a simple unidirectional modulation. *Without adding new experiments, could the authors expand on their conceptual model to clarify how S1–SC interactions give rise to bias across contingencies?*

We thank the reviewer for this insight, and agree that the different outcomes between unilateral S1 vs. SC inactivation warrants further clarification. We also acknowledge that the effects of direct SC inactivation are not the same as S1 inactivation effects.

As R1 notes above, direct SC inactivation strongly disrupts the motor output of the circuit (**Fig. 2**), consistent with a motor bias observed with direct SC inactivation in previous work by Thomas et al., 2023. In contrast, S1 inactivation reduced local SC sensory encoding and produced a *perceptual bias* independent of the motor contingency (now **Extended Data Fig. 6**).

To more clearly address these differences, the revised text is more deliberate in the use of the term 'bias'. We use "choice bias" to refer to a general, systematic shift in the probability of a yes/no response. This could arise from a "perceptual bias (shift in the criterion for reporting stimulus presence), which is distinct from "motor bias," or favoring one lick direction, regardless of sensory evidence.

Based on our working model outlined in 'R1-major #'1 above, SC contributes to both sensory evidence evaluation and action selection. The population activity within the early stimulus window was most predictive of eventual choice, suggesting that the state of SC at the time of the stimulus can explain the overall choice bias.

However, whisker S1 projects to non-sensory SC cells including the premotor/output neurons (Masullo et al. 2019, , Martín-Cortecero et al., 2023), and we cannot rule out whether S1 activity can directly modulate premotor licking/output neurons in lateral SC neurons outside of our recording site in central SC. However, our behavioral results of the reversed contingency experiments suggests that if there was a motor bias effect, these effects were minor compared to the perceptual bias induced by S1 inactivation.

We have revised the Results (lines 178-197) and Discussion (lines 426-440).

Minor:

Minor 1) Do SC neurons exhibit tunings for both ipsilateral and contralateral choice? Is there a bias in their proportion that is congruent with the lateralized perturbation effects shown in Fig 2?

We agree this is an interesting question. In the manuscript, for choice decoding analysis we used the early stimulus window (100ms from stimulus onset), before the response window opens (300ms after stimulus onset) to minimize confounds from licking, reward delivery, and movement vigor. These results show that the activity in whisker stimulus-responsive SC predicts the animals upcoming choices that remain robust with S1 inactivation. However, this early window does not allow us to determine whether the neurons are selectively tuned for ipsi vs. contralateral choice.

We attempted to quantify ipsi/contra selectivity by aligning SC activity to the first lick response. However, our task structure was not optimized for isolating this form of choice tuning due to the narrow window between the stimulus and the response window (300-ms from stimulus onset, stimulus lasts ~50ms). This obscured the boundaries between activity related to the stimulus, preparatory/motor activity, and reward. In addition, correct and error trials for the same stimulus differed in reward and licking vigor, further confounding estimates of choice selectivity. Given these limitations, we did not interpret the lick-aligned analyses as a reliable reflection of the prevalence of ipsi/contra selective neurons. Introducing a longer delay period would be better suited to directly address this question, analogous to those used by Thomas et al. 2023.

It is also worth noting that our SC recordings were targeted to the C2-whisker responsive region in intermediate SC. This area likely has distinct response properties compared to lateral SC where licking movement neurons have been described (Thomas et al., 2023, Ito et al., 2025, Lee and Sabatini, 2021). Consistent with this, our lick-aligned exploratory analysis did not reveal consistent bias in the proportion of ipsi:contra selective units, nor did we observe a reliable change between OFF/ON trials.

Thus, we cannot rule out the possibility that neurons in the lateral SC are also affected by S1 inactivation and may be disproportionately affected by S1 inactivation. Future experiments using recordings in a broader SC area, delayed response window, and cell-type specific recordings would be needed to help clarify this possibility.

Minor 2) Figs. 2o and 3n: Is there an increase in reaction time during bilateral S1 or SC silencing?

Given this role of SC in premotor licking control, we agree that reaction time would be a useful control to rule out global motor impairments elicited by silencing. This is an important point, as direct SC inactivation of lateral SC resulted in significant motor impairments: unilateral SC photoinhibition inhibited contralateral licking, and bilateral SC inactivation suppressed licking altogether (Thomas et al., 2023). It is thus possible that bilateral S1 or SC inactivation in our study may have had a global effect on licking motor output.

If bilateral inactivation primarily impaired sensory representation, we would expect latencies to increase during ON trials for near-threshold stimuli, with relatively little effect on no-stimulus or strong stimulus (amp 10) trials. In contrast, a global motor impairment would be expected to increase response latencies across all stimulus amplitudes, for both lick directions.

To address whether S1/SC inactivation produced global changes in response timing, we analyzed the timing of the first detected lick during the stimulus window (300-ms from stimulus onset, 2-s duration). However, there are a few important details about the lick latency measurements to consider given our task structure, which was not optimized as a reaction-time task.

For behavior-only experiments without neural recordings (all bilateral experiments), the data acquisition system (Arduino microcontroller) only captured first licks within the response window to register responses. This lick timing should be interpreted as the latency of the first *registered* lick rather than a precise measure of reaction time. Moreover, animals were trained with a 'go' response cue tone 300-ms after onset, and mice learned to respond after this window, which may obscure reaction times mice may be capable of for the shortest lick latencies (e.g., for the strongest stimulus amplitude amp 10). A second point is that for no-stimulus trials, there is no temporal cue to indicate when the stimulus would have occurred, leading to long and variable latencies that make left lick reaction times difficult to interpret.

Despite these limitations, the lick timing analysis provides a useful control for whether S1/SC inactivation produced broad delays in responding. We add these new analyses (**new Extended Data Fig 5**) of lick timing for S1 and SC inactivation corresponding to experiments shown in Fig. 2 and Fig. 3, respectively. During bilateral S1 inactivation, we did not detect significant changes in lick timing in either lick direction, or across stimulus amplitudes. Thus, the behavioral effects of bilateral S1 inactivation are unlikely to reflect a significant global impairment in licking.

For bilateral SC inactivation in Pitx2-Halo mice, the effect was more nuanced. Although bilateral SC inactivation had no discernible effect on task performance (**Fig. 2o-r**), we observed increased lick delays in right licks for higher stimulus amplitudes only, while no significant differences in left licks were detected (**Extended Data Fig. 6b**). Thus, bilateral SC inactivation did *not* produce a global motor impairment.

Together, these results suggest that bilateral inactivation of S1 or SC did not result in global impairments in licking motor output. It is possible that subtler latency effects are masked by large variability across subjects, or are confounded by mixed effects of sensory, decision-related, or motor preparatory changes. However, the present data do not suggest a broad inability to initiate licking.

As noted above, our optogenetic targeting was to the same C2-responsive region in SC as described for recordings (**Extended Data Fig. 7b**), medial to the lateral SC regions previously implicated in motor movements. In addition, Pitx2-Halo is also only expressed in a subset of intermediate SC neurons. The anatomical and cell type differences may explain the modest effects we observed during bilateral SC inactivation rather than severe licking impairments reported for bilateral SC inhibition (Thomas et al., 2023).

Because lick latency can reflect sensory evidence, decision bias, and motor preparation and output, we treated this analysis as a control for global motor deficits rather than as a direct readout for perceptual processing. We have revised the manuscript to include the registered lick timing data (**new Extended Data Fig. 5**) and added relevant text to the Results (lines 183-197) and Methods (lines 554-559).

Minor 3) Fig. 6f: The increase in SC decoding accuracy after S1 silencing is interesting. Can the authors provide further insights into what is driving this increase? Is this increase driven by hit trials, correct reject trials, or both?

In the revised, cross-generalized choice decoding analysis, decoders were trained on OFF trials and tested on held-out OFF or ON trials as suggested (R1, major # 2). Choice decoding remained significantly higher during S1 inactivation (**76.3** +/- 0.6 OFF vs. **79.9** +/- 0.5 for ON, $p = 3.5 \times 10^{-6}$), but the magnitude of this increase was modest (3.6 percentage points). We therefore avoid strong mechanistic interpretation.

Our decoding analysis was restricted to the early stimulus period (100-ms window from stimulus onset), before the earliest reward delivery. Thus, this choice-predictive activity reflects population activity during the stimulus period, before outcome-related activity (hit/misses). Furthermore, trial numbers were balanced across lick direction (right vs. left lick), which is yoked with reward outcome (lick right = hit, lick left = miss). Thus, the increase in decoding accuracy cannot be explained by a difference in the proportion of trial outcomes. We have revised the results section to clarify this, and include the effect sizes with revised cross-generalization analyses (lines 349-350).

Minor 4) Extended Data Fig. 6: Please indicate the neurons that are significantly modulated. Many SC neurons show disinhibition (OMI > 0). Are these changes statistically significant?

Thank you for the suggestion – we have reanalyzed the data and updated the figure accordingly, which we agree is a clearer representation of S1 inactivation-induced modulation. With S1 inactivation, only 4-5%

of SC neurons showed moderately increased activity (OMI > 0), while 18-19% were negatively modulated. We have revised the figure (now **Extended Data Fig. 8),** which clearly depicts significantly modulated units in colored bars. We have also modified SC inactivation controls (now **Extended Data Fig. 4**) with the same analysis for OMI in S1 vs. SC during Pitx2-Halo mediated SC inactivation.

Minor 5) Line 137: "…express inhibitory halorhodopsin in ~50% of excitatory neurons in the intermediate SC…" Is the 50% estimate based on cell counting in the current dataset or from a previous reference?

Thank you for pointing this out - the missing citation has been added (line 140, Masullo et al., 2019).

Minor 6) Fig 7: Do the dots represent individual mice?

Yes – in the original Fig. 7, the dots represented individual mice, but they were not clearly visible and obscured the average plots. For visual clarity, we have removed the dots from the main Fig. 7, but still show individual subject performance in **Extended Data Fig. 9.**

Minor 7) Line 356: "…S1 provides flexible control over decision-bias". Perhaps consider removing the word "flexible" since this was not explicitly tested.

Done.

Minor 8) Line 368-371: The sentence stating that S1's "major influence" is on decision bias rather than sensory encoding could be clarified. The data clearly shows that S1 affects decision bias via SC, but this does not preclude additional sensory role of S1, since bilateral S1 silencing did reduce sensitivity.

We completely agree -- we did not intend to rule out a sensory role for S1. The original sentence was:

> "*…S1's major influence on detection behavior is downstream modulation of decision bias, rather than limited to sensory encoding*"

which was meant to suggest that beyond its established role in sensory encoding, S1 exerts a major influence on decision bias. To avoid ambiguity, we revised the text to better clarify this distinction. The new related sentence reads (lines 485-6):

> *"S1 not only enhances sensory responses in SC but also modulates SC to shape decision criteria that guide action selection..*

Minor 9) In the method section (line 689,690), why are different number of units used for stimulus and choice decoding?

In the original manuscript, we did not match the number of units between stimulus and choice decoding analyses because we never directly compared stimulus vs. choice decoding accuracy. However, both reviewers 1 and 3 raised this concern, so we re-analyzed all decoding analyses to match unit numbers. The revised decoding balances (i) trials by lick direction (left/right); number of OFF vs. ON trials; (ii) the number of units across brain areas, and now (iii) across stimulus and choice decoding, by down-sampling

to the smaller eligible pool. This reduced statistical power, but did not change the general conclusions. The new analysis was applied to all decoding analyses and adjusted in **Fig. 4h-i** and **Fig. 5e-f**, and the Methods (lines 772-776) is now revised to reflect this.

Minor ***10)*** *A few typos:*
[x] Line 83: the figure reference should probably be Fig. 1a-b.
[x] Line 118, "overall changes in sensitivity did not differ". Perhaps the authors mean that overall sensitivity did not differ, or changes in sensitivity were small or inconsistent.
[x] Line 146: typo, "(Fig. d-g)" should probably be "(Fig. 2d-g)".
[x] Line 252: figure references should probably be Fig. 4d or 4e.
[x] Line 288 is missing a figure number.
[x] Line 507: typo, repetition of "turned on."
[x] Line 538, the reference to Extended Data Fig. 6 should probably be removed. → *should be EDF 6*
[x] Tick labels are misaligned in Fig 3d-e, i-j, n-o
[x] Figure 2i–m legend, the description may be missing the phrase "SC ips" before "inactivation."

We thank R1 for the careful reading and corrections--all items listed above have been corrected.

Reviewer #2 (Remarks to the Author):

This fascinating and rigorous study by Nam et al. investigates the coordination between sensory cortex and superior colliculus in the context of stimulus detection. Their conclusions are founded on psychometric evaluations of a Yes/No whisker detection task, paired with neuronal recordings and perturbations. The manuscript makes the following novel conclusions which contrast with traditional views of sensory cortex contributions to sensory detection: 1) sensory cortex causally contributes mostly to bias rather than sensitivity and 2) this function is dependent on the superior colliculus. The data, controls, and analyses appear to be of high quality. My most substantial concerns are with some of the interpretations, which can be addressed with additional analyses and discussion.

1. I'm stuck on other possible interpretations of a rightward shift of the psychometric curve. While I agree that 'bias' is the most straightforward interpretation, it may not be the only one. Consider two parallel sensory pathways with similar neurometric sensitivities but different thresholds. The low threshold system would normally be driving detection decisions as it would be activated by lower stimulus intensities. However, if removed, then the high threshold system would take over. This would appear as a shift in bias. Furthermore, this may map on to the lemniscal (low threshold, requiring S1) and paralemniscal (high threshold, possibly bypassing S1) whisker pathways. Can the authors rule out this organization?

We thank the reviewer for this thoughtful question. We agree that parallel pathways (thalamocortical (primarily leminiscal) vs. trigemino-collicular (primarily paralemniscal) would be consistent with the rightward shift in the psychometric curve during contralateral S1 inactivation, that could appear as a bias shift. S1 indeed encodes weaker stimuli more strongly than SC (**Fig. 4f, g**), consistent with such a model. However, several key results are inconsistent with a dual threshold model:

(1) The suggested dual-threshold model would predict that $S1_{contra}$ inactivation would selectively impair low-stimulus amplitudes, while minimally affecting false-alarm trials (yes/right licks to no-stimulus trials). However, we observe that the dominant effect of contralateral S1 inactivation is a global decrease in 'yes' responses, including no-stimulus trials (decreased false alarm rates, **Fig1. d, g**).

(2) Most importantly, the direction of threshold shift depended on which S1 hemisphere was inactivated – $S1_{ipsi}$ inactivation decreased detection threshold, which seems inconsistent with a simple loss of the low-threshold S1 pathway (previous studies indicate that acute inactivation of ipsilateral S1 does not significantly alter whisker stimulus responses in contralateral S1 relative to the stimulus (Shuler et al., 2001, Reig and Silberberg, 2016)).

(3) If S1 is the low-threshold, default pathway, SC lesions should minimally alter behavior. However, when SC was lesioned but S1 remained intact, performance was significantly impaired. Even the residual performance after SC lesions did not seem to be dependent on S1, since S1 inactivation in SC lesioned animals had minimal effect on the residual performance abilities. This further suggests that SC is an essential node for detection behavior in this task, rather than a parallel pathway that is normally bypassed when S1 is present.

Together, our experimental results seem more consistent with the interpretation that S1 modulates the baseline and sensory gain in SC, which itself is involved in action selection, rather than S1 acting as a default low-threshold pathway that is replaced by a high-threshold (SC) pathway during S1 inactivation; our working model is that S1 inactivation causes an interhemispheric balance that mediates the observed bias shift (described in detail in the above response to R1, major #1, and now in the Discussion section).

We agree that the suggested parallel/dual threshold model should be discussed, and likely the most common assumption in the field. We have therefore revised the Discussion to explicitly highlight this model (lines 410-420).

2. SC as the 'bottleneck'. The results displayed in Fig 2n-o are extremely interesting, and suggest active competition between SC hemispheres in setting the bias. However, the finding that bilateral suppression causes no change in the psychometric function would seem to undermine the statement in the abstract of "SC as the critical bottleneck in perceptual decisions" (as well as similar statements in the Results and Discussion). From my understanding of a bottleneck, I would expect suppression to block perceptual decisions (reduce response rates). Please clarify.

Our intended use of term "bottleneck" was to express that SC is the downstream node required for the expression of S1-induced bias shifts, including the possibility that other pathways to SC could mediate the S1-induced bias effect we observed (e.g., higher order sensory/motor cortex, basal ganglia). However, we see that the term "bottleneck" implies different claim than intended. We have revised the text throughout to remove the term throughout the text.

3. The 'unmasking' of an S1 contribution to sensitivity is very interesting (Fig 3m-q). Do you suspect that it is the same population of S1 neurons that contribute to both bias and sensitivity?

This is an important point. We do not currently know whether the same S1 neurons mediate both effects.

We interpret behavioral bias during unilateral S1 inactivation as a consequence of removing excitatory drive to downstream SC, rather than as evidence that bias is explicitly encoded in S1.

S1 projects to SC primarily via layer 5b neurons (newly added **Extended Data Fig. 3d, e)**, whereas stimulus amplitude is represented broadly across all layers in S1. The decrease in behavioral sensitivity during bilateral S1 inactivation may reflect disrupted sensory representation in S1, but is also consistent with decreased sensory representation in SC (**Fig. 6, a-d**). Thus, bias and sensitivity effects may arise from partially overlapping but mechanistically distinct contributions from S1.

4. An important missing reference is Luo and Maunsell 2015. By manipulating reward, the authors were able to behaviorally dissociate changes in bias vs sensitivity, and showed that changes in V4 visual cortex of monkeys track changes in sensitivity only. The conclusions of the Luo and Maunsell work conflict with the conclusions of the current study. Please address in the Discussion.

We thank the reviewer for this reference, which we have incorporated in the discussion (Line 473). Luo and Maunsell (2015) showed that attention-modulated changes in V4 activity corresponded to changes in sensitivity, and not changes in criterion, suggesting bias is encoded downstream of V4. In addition, since our manuscript submission, a new study by the same group (Ghosh & Maunsell, Feb 2026, Nature Communications) found that for a primate visual attention task, attentional modulation related changes in SC neurons reflect sensitivity and motor bias, but not perceptual bias.

We do not interpret our results as conflicting with these models. Importantly, our main claim is not that S1 neurons encode bias, but that unilateral S1 inactivation can bias perceptual choices, likely by altering downstream activity in SC.

In addition, several key differences are notable. In both primate studies, they examined endogenous attention-related modulation of V4 or SC, while our study uses causal perturbations of S1 to determine how this alters tactile detection via downstream circuits. Our data suggests that unilateral S1 perturbation shifts behavioral bias and alters SC activity, and that SC activity reliably predicts trial-by-trial choice during near-threshold detection. However, we do not interpret these findings to suggest that S1 or SC explicitly encodes bias as an abstract variable. Rather, we propose that unilateral S1 inactivation alters the balance of activity in downstream SC-dependent pathways, thereby changing the probability that sensory evidence is converted into a 'yes' report.

One difference is perhaps whether S1 activity tracks changes in sensitivity similar to V4 in the primate study. In our experiments, while unilateral S1 perturbations did not reveal significant changes in sensitivity, bilateral S1 inactivation resulted in a modest but significant decrease in sensitivity. The relatively small effect size we observed is likely task-dependent; numerous studies, including our prior work, indicate that S1 is not essential for simple tactile detection (Hutson and Masterton, 1986, Hong et al., 2018, Park et al., 2020, Waiblinger et al., 2022, Ryan et al., 2022), but is required for just about any discrimination task beyond detection. SC itself can encode the tactile stimuli used in our task, and are more likely to contribute to the sensory and decision process. Thus, cortical modulation of behavior may depend strongly on the downstream circuit being engaged. We have added both of these references and extend our discussion accordingly (lines 479-484).

5. The Introduction provides a very superficial overview of the relevant SC, sensory cortex, and barrel cortex literature related to sensory detection as well as computational and neural studies regarding the

establishment of bias. More in-depth treatments of these topics would help contextualize the current work. The final two paragraphs (and especially the final paragraph) related to the current study can be significantly shortened if needed. [On further reading, some of the relevant background information is contained in the Results, which can be moved to the Introduction.]

We agree that the original introduction did not provide sufficient contextual information to properly motivate the current work. We have completely revised the Introduction accordingly (lines 43-80).

6. It would be very interesting to know whether inhibition of S1ipsi in the Go/Nogo context also causes a leftward shift in the psychometric curve (Extended Data Fig. 4k-n). If so, it would generalize the bidirectional control beyond the forced choice task design. If the data is not already in-hand, this could be a discussion point.

We agree that this would be an interesting and informative experiment, but these data are not currently in-hand. To our knowledge, prior work has not tested whether acute ipsilateral inactivation alters bias in a go/nogo detection task, as most studies focus on contralateral S1 manipulations (notably, even for negative controls of acute inactivation experiments, most studies use visual cortex, rather than ipsi-S1).

Our working model is that S1 inactivation alters SC's baseline excitability, gain, and interhemispheric balance that transform sensory inputs into behavioral action. Our reversed contingency experiments are consistent with contralateral S1 inactivation inducing a perceptual bias rather than motor bias (although we cannot rule out weak motor bias effects that may be masked by the stronger effect of perceptual bias, see response to R1, question 1-iv).

This model predicts that ipsilateral S1 inactivation would have minimal effect on the sensory encoding in S1 or SC. However, ipsilateral S1 inactivation should shift the balance between the SC hemispheres (similar to added **Extended Data Fig. 10**), and behaviorally this would result in a perceptual bias that increases both hit and false alarm rates without altering sensitivity, similar to the yes/no task. We have revised the Discussion to propose this working model prediction as suggested (Lines 450 - 453).

7. The SC lesion data (Fig 7b) appears markedly different from the SC opto-suppression data (Fig 2d-h). It would be helpful to include similar quantifications for the SC lesion data. If SC lesions are indeed reducing sensitivity, how would this impact the conclusions of the study?

We thank the reviewer for raising this important point. We agree than the effects of SC lesions are different from acute optogenetic suppression (Fig. 2d-h). We now clarify in the Results that lesions produce broader and more variable impairments, likely because they disrupt a larger and more diverse SC population than Pitx2-Halo mediated optogenetic suppression. Importantly, this difference does not alter our main conclusion that SC is required for expression of the S1-induced behavioral effect.

We performed the same psychometric analysis on the SC lesion data to quantify sensitivity (slope, b_1) and bias (intercept, b_0). However, SC lesions produced more severe behavioral impairments than optogenetic silencing. As a result, for several post-lesion sessions (3/12 sessions), psychometric fits were not significant ($p \gg 0.05$), preventing reliable estimation of bias and sensitivity. In addition, with long-term SC lesions, animals may gradually adjust their behavioral strategy across sessions, further complicating interpretation of these measures.

We therefore presented performance as the probability of detection (P(yes) across stimulus amplitudes, a model-free measure that incorporates all of the sessions. To address the reviewer's request, we additionally analyzed slope and bias for sessions that could be reliably estimated (9/12) and have added them to the Results section. We note, however, that this exclusion likely underestimates the full effect of lesions, as the most severely impaired sessions are disproportionately omitted from this analysis. These analyses have been added to **Extended Data Fig. 9**, and Results (lines 370-377)

8. I agree that the current study provides a compelling hypothesis regarding the paradox of acute vs chronic causality. However, to truly 'resolve' the paradox, one would need to actually demonstrate the proposed mechanism of recovery.

This is a fair point. We revised the text to state that our findings "may also help explain how acute cortical perturbations can lead to apparent sensory impairments during detection behaviors, even when the primary sensory cortex is not strictly required for simple detection behaviors " (lines 490-492).

9. Entirely up to the authors: it may be useful to include the finding of S1 modulating bias in the title.

We thank the reviewer for this suggestion. We revised the title to: "Somatosensory cortex shapes perceptual ~~decisions~~ bias via the superior colliculus"

Reviewer #3 (Remarks to the Author):

Nam et al. provide compelling evidence through an impressive array of rigorous experiments to show that SC activity controls the decision-making threshold. Moreover, they show that S1's role in decision-making is mediated through SC. This work provides a critical and long-overdue understanding of the cortical contribution to behaviors not involving the discrimination of fine-scale stimulus features. Therefore, this work will have a long-lasting impact on the field by improving the way people design experiments and interpret results. Below are some relatively minor comments that may help improve the manuscript. I have no major concerns.

1. In this manuscript, the authors focus on response probability as the decision outcome. Is there a shift in response latency that is consistent with the observed shift in decision bias? For instance, when you silence ipsilateral SC, does response latency shorten for the lower amplitude stimuli?

[This question relates to R1 minor #2, please also see response above] We agree that response latencies are an important parameter. We have added lick timing data (new **Extended Data Fig.5**). However, changes in response latency are not only affected by decision bias, but strongly influenced by the strength of sensory evidence and motor processes (e.g. lick initiation, response vigor, etc). Furthermore, in our yes/no detection task, correct trials for lick right vs. left were not equivalent. For example, the whisker stimulus can immediately elicit a lick-right response, while no-stimulus trials did not indicate when the stimulus would have occurred. Animals typically waited longer (500-600 ms) and with larger variability to lick left to report the absence of a stimulus.

Due to these multiple variables that affect lick latency and task structure, our response latency results were not easily interpretable. S1 inactivation did not produce significant changes in lick latency for any of the perturbation experiments (contra-, ipsi-, or bilateral inactivation). Both SC_{contra} (left) and bilateral SC inactivation increased right lick latencies for near-threshold stimulus amplitudes as well as strong amplitudes, but no changes in weaker amplitudes were detected. SC_{ipsi} (right) inactivation increased left lick latencies only for amp 0. Lick latency measurements have been added as a new figure **(Extended Data Fig. 5**) and added relevant text to the Results (lines 183-197) and Methods (lines 562-567).

2. They show that "SC inactivation" does not alter S1 activity. However, their data show that SC inactivation is quite modest. Might this explain their result? It would be useful to show the SC dC1873eactivation results in the format of extended data fig. 6.

Pitx2-Halo-mediated SC inactivation is indeed quite modest (now **Extended Data Fig. 4**). Despite the modest changes in SC activity, the behavioral effects are highly significant (**Fig. 2**). The control experiments in Extended Data Fig. 4 suggest that this level of perturbation alters behavior similar to S1 inactivation, and importantly, that this cannot be explained by an indirect suppression of S1 activity. Pitx2 only labels around half of all excitatory neurons restricted to the intermediate layers (Masullo et al., 2019), and only subset of these neurons responds to whisker stimuli. It is quite possible that stronger, more comprehensive SC inactivation can alter S1 activity (e.g., via the SC-POm-S1 pathway described in Gharaei et al.), but does not alter the conclusions of the current study.

We agree with the reviewer that showing the histograms for **Extended Data Figure 4** would be helpful. We have added these panels (**EDF3 f, g, m, n**) and in keeping with Reviewer 1's suggestions, have additionally indicated the units that were significantly modulated.

3. Did you trying restricting your analysis to L5 neurons in S1 during SC silencing?

Our original figure showed that on average*,* Pitx2-Halo mediated inactivation of SC had no significant effect in S1. S1 projects directly to SC primarily via L5b (Extended Data Fig. , so it is possible that averaging across layers could mask the most relevant changes occurring in L5b. This is a fair point—we determined whether restricting analysis to L5 neurons in S1 would reveal SC-dependent modulation. We have now analyzed the S1 recordings by cortical depth, but find no evidence for systematic modulation in any layer (see plot below).

Across all S1 units recorded during SC, average firing rates did not significantly change with SC inactivation (Extended Data Fig.4 j-l). Only 6/70 units across all cortical layers showed significant optogenetic modulation (OMI, $p<0.05$). Within L5b, only 1/8 (2/15 in L5a+Lb combined) were significantly modulated, and showed an increase, rather than decreased activity.

Thus, across all layers, we did not observe a consistent suppression of S1 activity during SC inactivation. These results argue against the possibility that the behavioral effects of SC inactivation are mediated by indirect suppression of S1. Given the relatively small sample size when divided across cortical layers, we provide the layer-specific analysis here but did not include it in the main manuscript.

Cortical layers were approximated based on distance of the tip of the laminar electrode array (single shank, 64 channels, 1250um span) from pial surface, as previously shown in Hong et al., 2018, and Rodgers et al., 2021, based on cortical depth measurements in Hooks et al., 2011).

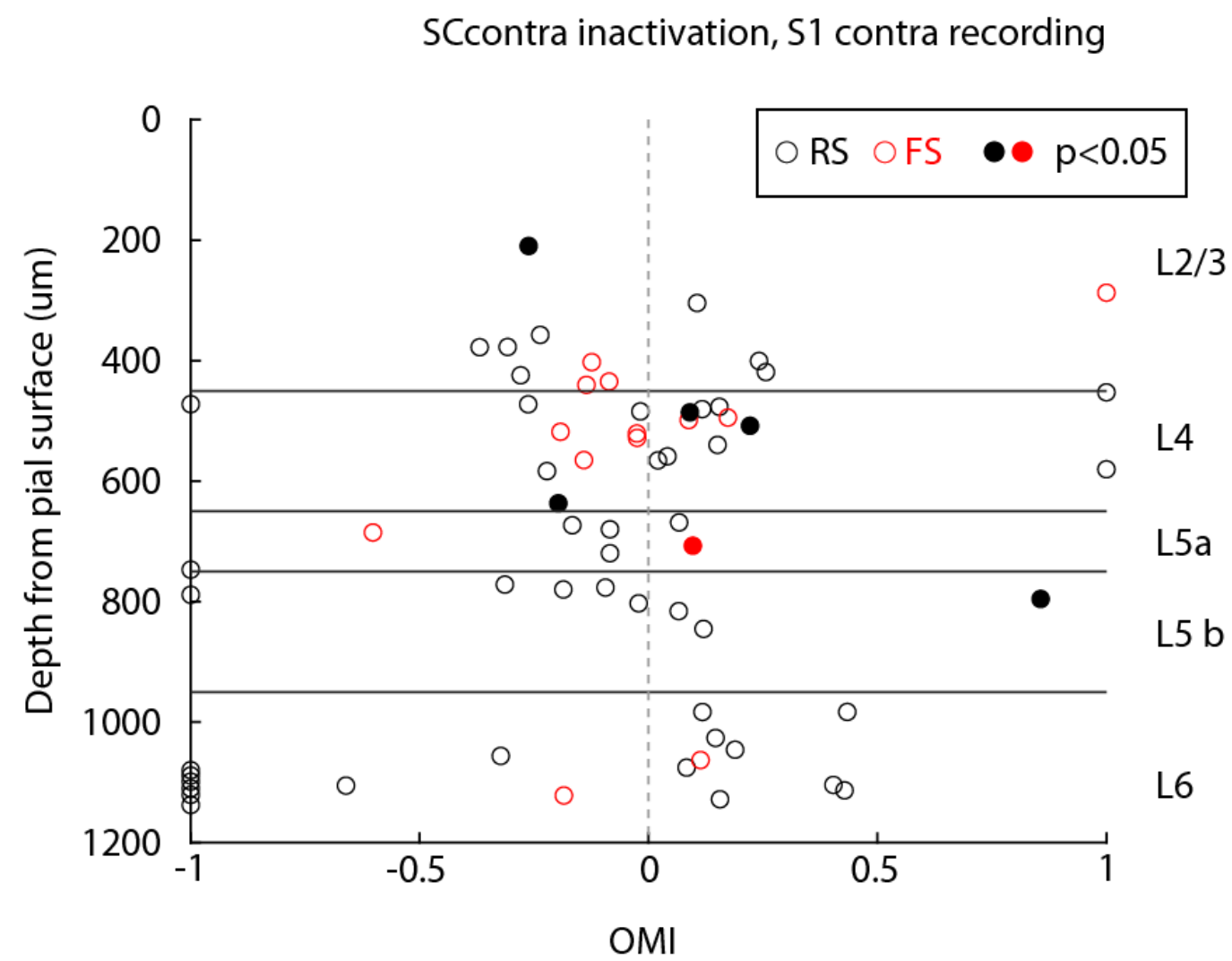

Figure. SC inactivation does not significantly alter S1 activity across layers. Black circles indicate putative excitatory neurons (RS), and putative inhibitory neurons (FS) are in red. Units with significant OMI modulation (n = 4RS, 1FS, $p<0.05$) are indicated as filled circles.

4. On line 191, they authors state that ascending whisker inputs are entirely lateralized. While technically true, ipsilateral responses do occur in S1 and are quite prevalent for the A-row barrels. However, these ipsilateral responses originate from the contralateral S1, which is not really considered the ascending pathway. So, this is a minor detail.

We agree that ipsilateral whisker responses are detected in S1, and prior work indicates that these originate from cross-callosal inputs from the opposite S1 hemisphere (e.g., Reig and Silberberg, 2016), and therefore do not reflect ascending sensory input. To clarify this distinction, we have added a parenthetical note in the main text (lines 210-212):

> *In the sensory evidence model, because ascending whisker inputs are lateralized, originating from the contralateral whiskers,* $S1_{ipsi}$ *inactivation should minimally affect sensory responses in* $S1_{contra}$[11,41–44] *and would have minimal impact on behavior (although S1 neurons can respond to ipsilateral whisker stimuli, this is thought to originate from cross-callosal inputs from S1 in the opposite hemisphere rather than uncrossed ascending input (Shuler et al., 2001; Reig and Silberberg, 2016).*

5. on Line 689 they state the use of 423 neurons for stimulus decoding and 282 neurons for choice decoding. It is not clear why there are a different number of neurons used.

R1 had similar concerns (See response to R1-minor #9), and we have now performed new analyses to match units across stimulus and choice decoding analyses. Adjustments have been made accordingly to

Fig. 4h-i and **Fig. 5e-f**, and the Methods (lines 772-776).

6. The authors use the Pitx2-Cre line to silence SC neurons. Might there be non-Pitx2 neurons that are more specialized for sensory coding and could affect stimulus sensitivity?

We agree that non-labeled neurons in the Pitx2-Halo experiments could contribute to tactile sensory encoding and affect sensitivity. As noted above, Pitx2 only labels around half of all excitatory neurons restricted to the intermediate layers (Masullo et al., 2019), and only subset of these neurons are whisker responsive. In addition, our anatomical data show that S1 projects extend across upper and lower portions intermediate SC, as well as deep SC (**new Extended Data Fit. 3**), whereas Pitx2-Halo appears to label mostly the lower portion (**Fig. 2c**). Thus, our Pitx2-Halo manipulation did not silence all sensory-responsive SC neurons.

We have revised the Discussion (lines 441-449) to note that our data support a role for Pitx2-halo neurons mediating choice bias (likely motor bias based on new latency data, new Extended Data Fig. 5), but do not exclude the possibility that other SC populations contribute to tactile sensitivity. We also note that SC lesions did not produce a clear change in sensitivity (newly added **Extended Data Fig. 9 panels e, d, f,** in response to R2 #7), but this was difficult to interpret because in a few cases, SC lesions caused strong behavioral impairments dominated by changes in choice bias. Future experiments using broader SC manipulations, (or SC cells that receive trigeminal sensory input) will be needed to determine whether sensory encoding and decision bias are mediated by distinct SC neurons.

7. The authors show that bilateral S1 silencing reduces sensitivity. What S1-->target circuit could be contributing to the reduction in response probability at near-threshold stimulus intensities?

We thank the reviewer for this important question. The modest reduction in sensitivity during bilateral S1 silencing suggests that S1 contributes to the detection of near-threshold stimuli. One possibility is that the strong bias shift produced by unilateral S1 inactivation masks a weaker contribution of S1 to perceptual sensitivity, which becomes apparent only when both hemispheres are silenced and the lateralized bias is neutralized.

While our results do not identify a definitive downstream circuit, several key results suggest a plausible mechanism via SC. First, S1 neurons show a trend toward stronger responses than SC for near-threshold stimulus amplitudes (Fig. 4f), consistent with S1's role in enhancing weak sensory signals. Second, S1 inactivation reduces stimulus-evoked activity in SC, particularly for near-threshold stimuli (Fig. 5l, Fig. 6b) and decoding analyses indicate that these changes in SC sensory responses strongly predict changes in the detection probability.

Together, these findings suggest that S1 may facilitate detection of weak stimuli, at least in part by amplifying sensory representations in SC. However, S1 also projects directly to the striatum, thalamus and the trigeminal brainstem—S1 inactivation may alter ascending sensory inputs to decrease ascending sensory responses, or basal ganglia to alter motor output.

We add discussion of these alternative pathways to the Discussion (lines 466 – 473).

8. Seems worth citing James Sprague:

Thank you for catching this -- this was an oversight. We have included the Sprague effect in our discussion and reference accordingly (line 461).

Additional revisions:

We previously used a gaussian kernel to smooth the data for analysis, which underestimated the stimulus-evoked firing rates, particularly considering the initial sharp stimulus-evoked response to whisker deflection. We now perform all neural data quantification and statistics on un-smoothed spike data, with gaussian smoothing only used for visualization of PSTHs in **Fig. 4** d,e and 5e, f, h, i). This has been clarified in the Methods (~line 667). Accordingly, we updated the plots in **Fig. 4 f, g**, which show significant differences between S1 and SC (original data showed the same trends but did not reach significance levels). This did not apply to other neural data analyses (including decoding analysis), which used the original unsmoothed spike data, and remain unchanged.
